# Structural tuning of tetrazole-BODIPY Ag(i) coordination compounds *via* co-ligand addition and counterion variation†

**DOI:** 10.1039/d5ce00197h

**Published:** 2025-03-20

**Authors:** Matthias Schöbinger, Martin Huber, Berthold Stöger, Christian Hametner, Peter Weinberger

**Affiliations:** a Institute of Applied Synthetic Chemistry, TU Wien Getreidemarkt 9 1060 Vienna Austria matthias.schoebinger@tuwien.ac.at peter.e163.weinberger@tuwien.ac.at; b X-Ray Center, TU Wien Getreidemarkt 9/164 1060 Vienna Austria

## Abstract

The coordination properties of a previously described fluorescence active ligand (**L**), consisting of a coordinating unit (1*H*-tetrazol-1-yl) and a fluorophore (4,4-difluoro-4-bora-3*a*,4*a*-diaza-*s*-indacene (BODIPY) derivative) towards Ag(i) were investigated. Additionally, the influence of different anions (BF_4_^−^, PF_6_^−^, PF_2_O_2_^−^, ClO_4_^−^, ReO_4_^−^ and NO_3_^−^) and a co-ligand (CH_3_CN) on the crystal structure formation and intramolecular interactions of the Ag(i) coordination compounds was studied. Beside structural investigations *via* single crystal X-ray diffraction, bulk characterization of the coordination compounds was conducted in both solution and solid-state, including NMR (^1^H, ^11^B, ^19^F, ^31^P and ^13^C), ATR-IR, UV-vis and photoluminescence spectroscopy as well as PXRD. Eleven distinct coordination compounds are reported, each falling into one of four classes: the first group (I) comprises of a mononuclear complex, whereas group (II) consists of dinuclear complexes with ligand bridged metal centers (Ag(i)) and weak intermetallic interactions (∼4 Å). Group (III) likewise includes dinuclear complexes, but the bridging mode was prevented and the Ag–Ag distance was reduced (∼3.2 Å) upon the addition of a co-ligand. Group (IV), a structurally diverse category consists of coordination polymers, which in some cases show even shorter intermetallic contacts (<3.1 Å). All investigated coordination compounds exhibit photoluminescence in the solid state, with structurally dependent emission maxima distinct from those of the ligand.

## Abstract 2

Structural properties – particularly Ag–Ag interactions – of coordination compounds comprising a tetrazole-BODIPY ligand and Ag(i) were tuned by adding a co-ligand and varying the counterions, resulting in distinct emission maxima.

## Graphical abstract

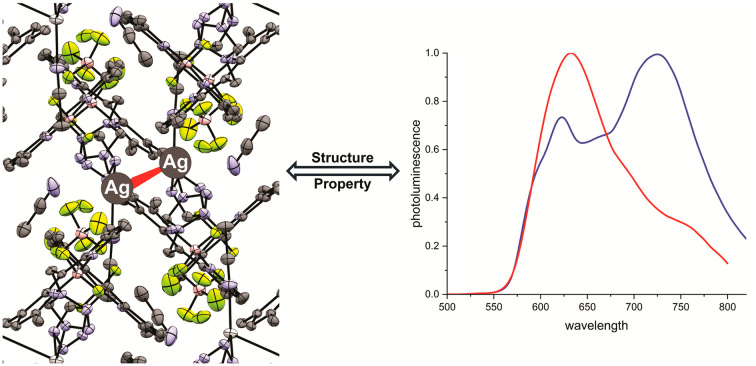

## Introduction

Silver (Ag), one of the three coinage metals (Cu, Ag, Au), exhibits diverse coordination behavior in its singly charged cationic state (Ag(i)), enabled by its filled-shell d^10^ electron configuration. The absence of any crystal field stabilization energy does not lead to a preferred coordination geometry,^1^ enabling coordination numbers from two to nine.^2^ Additionally, Ag may establish coordination bonds to N, S, and O based versatile mono- or multitopic ligand systems.^3^ If such multitopic ligands and/or counterions are used, not only classical (0D) coordination compounds can be formed, but also coordination polymers (CPs) like chains (1D), sheets (2D) and 3D networks can be built.^4^

Further, Ag(i) exhibits the ability to form intermetallic (argentophilic, Ag(i)–Ag(i)) interactions at sub-van der Waals distances (∑*r*_vdW_(Ag,Ag) = 3.44 Å), either in a ligand-supported^2,5,6^ or unsupported^7–9^ manner. These interactions are comparable to hydrogen bonding in terms of binding energy (5–15 kcal mol^−1^), the ability to form multiple interactions and lack of strict directionality.^10^ The primary impact of Ag(i)–Ag(i) interactions is structural, facilitating cross-linking between monomeric units or sheets, which promotes the formation of CPs,^2,7^ and polynuclear metal clusters.^2,10^ Additionally, these interactions significantly influence the physicochemical properties of a compound or material. In particular, they can alter fluorescence emission behavior, which may even be quenched upon the insertion of additional molecules, enabling sensor applications.^10,11^ In some cases, partially covalent interactions can form between Ag(i) (acceptor) and an adjacent H–C (donor).^12,13^ Depending on the distance between donor and acceptor one can differentiate between agostic (1.8–2.3 Å) and anagostic (2.3–2.9 Å) interactions.^14^

Ag(i) CPs are increasingly being investigated due to their potential applications *e.g.*, as antimicrobial agents,^15–17^ as novel materials,^18^ in catalysis,^19,20^ in photoluminescence (PL) applications,^21–23^ as sensors^11,24,25^ and as anion exchangers.^20^

Ligand systems consisting of heterocyclic aromatic azoles are frequently used when designing multidimensional CPs.^13^ Besides diazoles^26–28^ and triazoles,^29^ particularly tetrazoles^3,7,9,30^ have the ability to function as a bridging ligand through multiple N-donors. Tetrazole (tz) exists in three isomeric forms (1*H*-tz, 2*H*-tz, 5*H*-tz), each capable of mono- or di-functionalization (1,5-; 2,5- and 5,5-disubstituted derivatives).^31^ The most frequently used tz are C- or 1,5-substituted, due to their application in medicine.^32^ However, also as ligands for Ag(i) coordination compounds C-substituted tz^2,3,9,33,34^ are more often used than N-substituted tz.^15,35,36^ Keeping in mind that four different nitrogen atoms can independently function as a donor for the complex bond depending on the substitution pattern, tz derivatives represent a multifaceted ligand system. This versatility was first shown by Bodner^37^ in 1972 and Carlucci^30^ in 1999 by crystallizing tz bridged dimeric and 3D coordination compounds, respectively. In addition to multitopic ligands, counterions with multiple donor atoms can facilitate the formation of Ag(i) CPs (*vide supra*).^15,16,38,39^ The structural influence of the counterion on the CP is mainly dependent on its geometry and its donor properties as shown in literature.^39–41^

**Chart 1:**
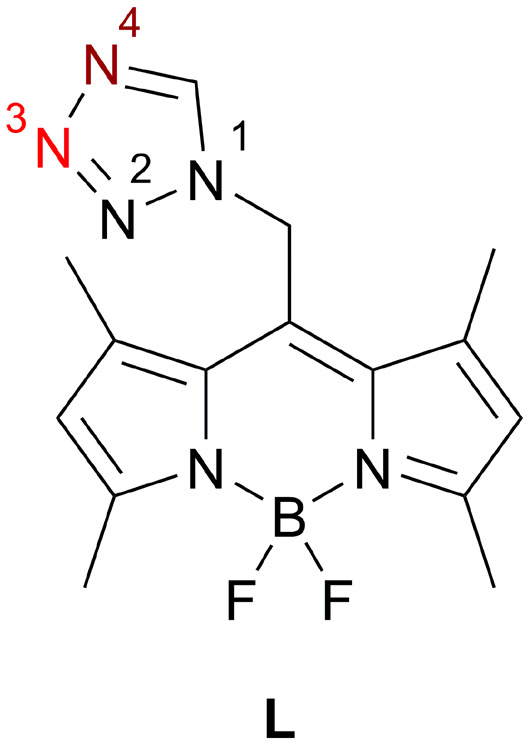
Photoluminescence active ligand (**L**), observed coordination site for monocoordinating mode dark red (N4); for bridging mode dark (N4) and light red (N3).

In this contribution, we report on the structural and PL properties of a novel family of Ag(i) coordination compounds formed with a recently disclosed tz-based ligand (4,4-difluoro-1,3,5,7-tetramethyl-8-[(1*H*-tetrazol-1-yl)methyl]-4-bora-3*a*,4*a*-diaza-*s*-indacene, **L**, Chart 1).^42^ The ligand carries a BODIPY (4,4-difluoro-4-bora-3*a*,4*a*-diaza-*s*-indacene) moiety as the photoactive component, chosen for its remarkable PL characteristics, including high fluorescence quantum yields,^43^ strong absorption,^44^ excellent thermal and photochemical stability,^45^ and sharp fluorescence emission bands spanning the visible to near-infrared (NIR) spectrum.^46^

In order to gain greater insight into the coordination behavior of these compounds, variations of the geometry and the donor properties of the counterion (BF_4_^−^, PF_6_^−^, PF_2_O_2_^−^, ClO_4_^−^, ReO_4_^−^ and NO_3_^−^) as well as the addition of a co-ligand (CH_3_CN) were performed and the resulting impact on crystal structure, intramolecular bonding (Ag(i)–Ag(i), C–H⋯Ag) and PL properties investigated.

## Results and discussion

### Synthesis

Ligand **L** was synthesized following a protocol previously established by our group.^42^ The synthesis of the coordination compounds 1–4, 6–8, 10 and 11 was straight forward by mixing **L** with the Ag(i) salt of different anions (BF_4_^−^, PF_6_^−^, PF_2_O_2_^−^, ClO_4_^−^, ReO_4_^−^ and NO_3_^−^) in acetone (ace) or acetonitrile (CH_3_CN) and stirring at elevated temperature (40 °C) over night. The coordination compounds were precipitated and washed with diethyl ether (Et_2_O). The molar ratio **L** : Ag(i) was fixed at 2 : 1 for all syntheses. However, in the case of 4, 6, 10 and 11 excess ligand was removed during the workup process, since less equivalents of the ligand were needed to build these structures (Table 1). It has to be noted that unlike in 1, 3, 7 and 10 in the case of 11 the use of CH_3_CN as solvent did not lead to a coordination of CH_3_CN as co-ligand, as has already been reported in the literature.^39^ All syntheses were monitored by IR and NMR spectroscopy as well as powder X-ray diffraction (PXRD), whereby the shift in the tetrazolic CH signal in ^1^H-NMR and IR spectroscopy was used as a quick indicator of successful complexation. 5 was found as a side product during the crystallization of 4 and 6, while 9 was found as a side product during the crystallization of 8. Therefore, 5 and 9 could not be characterized using bulk analysis methods. Moreover, 6 was also found during crystallization of 4 in ace due to solvolysis of the PF_6_^−^ anion.

**Table 1:** Composition and basic structural characteristics of coordination compound 1–11

Counterion	Compound	Formula	Nuclearity or dimensionality	Crystallographically independent Ag-atoms	Coordination number	Coordination geometry of Ag(i)	Ag environment
BF_4_^−^	1·2CH_3_CN	[Ag_2_**L**_4_(CH_3_CN)_2_](BF_4_)_2_·2CH_3_CN	Dinuclear	1	4	Pseudo tetrahedral	Ag, 2 × ***N****4* tz, **N**CCH_3_
	2·Et_2_O**·**ace	[Ag_2_**L**_4_](BF_4_)_2_·Et_2_O·ace	Dinuclear	1	3	Trigonal planar	2 × ***N****4* tz, ***N****3* tz
PF_6_^−^	3·2CH_3_CN	[Ag_2_**L**_4_(CH_3_CN)_2_](PF_6_)_2_·2CH_3_CN	Dinuclear	1	4	Pseudo tetrahedral	Ag, 2 × ***N****4* tz, **N**CCH_3_
	4	[Ag**L**_3_](PF_6_)	Mononuclear	1	3	Trigonal planar	3 × ***N****4* tz
PF_2_O_2_^−^	5	[Ag_2_**L**_4_(PF_2_O_2_)_2_]	Dinuclear	1	4	Pseudo tetrahedral	2 × ***N****4* tz, ***N****3* tz, **O**PF_2_O
	6	{[Ag_5_**L**_4_(H_2_O)(PF_2_O_2_)_5_]}_∞_	1D	5	3, 4, 4, 5, 6	—a	—a
ClO_4_^−^	7·2CH_3_CN	[Ag_2_**L**_4_(CH_3_CN)_2_](ClO_4_)_2_·2CH_3_CN	Dinuclear	1	4	Pseudo tetrahedral	Ag, 2 × ***N****4* tz, **N**CCH_3_
	8	[Ag_2_**L**_4_(ClO_4_)_2_]	Dinuclear	1	4	Trigonal pyramidal	2 × ***N****4* tz, ***N****3* tz, **O**ClO_3_
	9	{[Ag_5_**L**_4_(H_2_O)(ClO_4_)_5_]}_∞_	1D	5	3, 4, 4, 5, 6	—a	—a
ReO_4_^−^	10·CH_3_CN	{[Ag**L**_2_(CH_3_CN)(ReO_4_)_2_]·CH_3_CN}_∞_	1D	1	5	Distorted square pyramidal	2 × ***N****4* tz, **N**CCH_3_, **O**ReO_3_
NO_3_^−^	11	{[Ag_2_**L**_2_(NO_3_)_2_]}_∞_	1D	2 (4)	4, 5, 5, 6	—a	Ag, ***N****4* tz, ***N****3* tz, **O**NO_3_, **H**C

a — multiple – for details, see section Crystal structures.

### Bulk characterization

The analytical data of bulk samples in solution (NMR (^1^H, ^11^B, ^19^F, ^31^P and ^13^C)) and solid state (ATR-IR, PXRD, UV-vis and PL) for the coordination compounds 1–4, 6–8, 10 and 11 are shown in the ESI.† According to PXRD, the bulk powders of the coordination compounds 1–4, 6–8, 10 and 11 are single-phase. The coordination compounds 1–3, 7 and 10 crystallize as solvates (*vide infra*). For these compounds, the solvate molecules are also present in the bulk. To enhance readability in this chapter, the compound numbers for these five coordination compounds will refer to their solvates (*e.g.*: 1 ≙ 1·2CH_3_CN).

The main difference between the IR spectra of the uncoordinated ligand **L** and its Ag(i) coordination compounds is observed in the position of the *ν*_CH(tz)_ band. The coordination compounds 1, 2, 7 and 10 show the biggest shift from 3133 cm^−1^ in **L**^42^ to 3147 cm^−1^ after complexation. Smaller shifts of the aforementioned band to higher wavenumbers are shown by 3 and 4 (3144 cm^−1^), 8 (3137 cm^−1^) and 11 (3141 cm^−1^). For 6, the *ν*_CH(tz)_ band shift is not clear due to low intensities in the spectral region of interest. Additionally, the IR spectra of the coordination compounds show characteristic bands of each anion. For 1 and 2 only the asymmetric deformation mode of the BF_4_^−^ anion is visible at 520 cm^−1^.^47^ The stretching mode of the PF_6_^−^ anion in 3 and 4 appears as broad bands at 836 cm^−1^ and 829 cm^−1^, respectively. However, the bending mode of the same anion appears as sharp bands at 558 cm^−1^ and 556 cm^−1^, respectively.^48^ The IR spectrum of 6 displays the P–F stretching mode of the PF_2_O_2_^−^ anion at 839 cm^−1^.^49^ The symmetric stretching mode of the ClO_4_^−^ anion in 7 and 8 appears as a band at 460 cm^−1^ with very low intensity. Moreover, in 7 the degenerated asymmetric bending mode of the non-coordinating anion is visible at 621 cm^−1^. However, in 8 this mode splits up due to reduction in symmetry from *T*_d_ to *C*_3V_ geometry after coordination of the ClO_4_^−^ anion and therefore two bands appear at 618 cm^−1^ and 624 cm^−1^.^50–52^ In 10, the ReO_4_^−^ anion is also coordinated to an Ag-atom and therefore its *ν*_3(Re–O)_ vibrational mode is split into two bands which appear at 910 cm^−1^ and 887 cm^−1^.^41,53^ For 11, the various coordination modes of the NO_3_^−^ anion and the disorder of the metal centers do not allow a precise assignment of the observed bands at 703 cm^−1^ and 794 cm^−1^ to specific vibrational modes.

Nitrobenzene-d_5_ was used as NMR solvent, because it showed the best solvation ability for the coordination compounds and no coordination to Ag(i) was observed. Repeated ^1^H-NMR measurements showed good stability of the coordination compounds in solution over 48 h. The most prominent difference between the ^1^H-NMR spectra of **L** and the described coordination compounds is a downfield shift of the tetrazolic H-atom singlet, which has been previously reported in literature.^15^1 and 2 exhibit a difference of 0.63 ppm from 9.19 ppm in **L** to 9.82 ppm after complexation. However, 3 and 4 show a smaller downfield shift of 0.53 ppm in the tetrazolic H-signal. 6, 10 and 11 show even smaller shifts in comparison to **L** of 0.39 ppm, 0.16 ppm and 0.11 ppm, respectively. Interestingly, the coordination compounds with ClO_4_^−^ (7 and 8) as the anion exhibit different shifts, which contrasts with the compounds containing BF_4_^−^ (1 and 2) and PF_6_^−^ (3 and 4), where the shifts are identical for each pair. 7 exhibits a chemical shift of 9.83 ppm, but the tetrazolic H-signal of 8 is even more downfield shifted to 9.92 ppm. We attribute this to the fact that the ClO_4_^−^ anion is coordinated to Ag(i) in 8. Additionally, all investigated coordination compounds show small downfield shifts in comparison to **L** of the bridging –CH_2_– group between the BODIPY core and the tz moiety. Moreover, the co-ligand (CH_3_CN) is also visible in ^1^H-NMR for 1, 3, 7 and 10 at around 2.10 ppm.

As expected, the PF_6_^−^ anion of 3 and 4 shows as a septet at around −143 ppm in the ^31^P-NMR spectra and the PF_2_O_2_^−^ anion of 6 shows as triplet at around −14 ppm in the ^31^P-NMR spectra. The ^11^B-NMR spectra of **L** and all coordination compounds exhibit a triplet at around 0.60 ppm originating from the BODIPY core in **L**. Additionally, the BF_4_^−^ anion of 1 and 2 is visible as a singlet at around 0.37 ppm in ^11^B-NMR spectra. Since the anions of 1–4 and 6 contain F-atoms, the ^19^F-NMR spectra exhibit characteristic signals for BF_4_^−^, PF_6_^−^ and PF_2_O_2_^−^ beside the quartet around 145 ppm which originates from the BODIPY core of **L**.

The solid-state UV-vis spectra of the coordination compounds 1–4, 6–8, 10 and 11 closely resemble those of ligand **L**. The spectra exhibit a high absorption plateau between 250 nm and 580 nm, featuring several local maxima and minima, as well as a global maximum that varies only slightly for each coordination compound (Fig. S28†). Above 580 nm, the absorption decreases to a low level without any specific features. At 424 nm, **L** and 1–4, 6–8, 10 and 11 share a common local minimum at a high absorption level which was chosen as excitation wavelength for the PL studies. Due to high absorption levels from the ligand at wavelengths <580 nm the Ag(i)–Ag(i) interaction at around 260 nm could not be observed.^35^

**Fig. 1:**
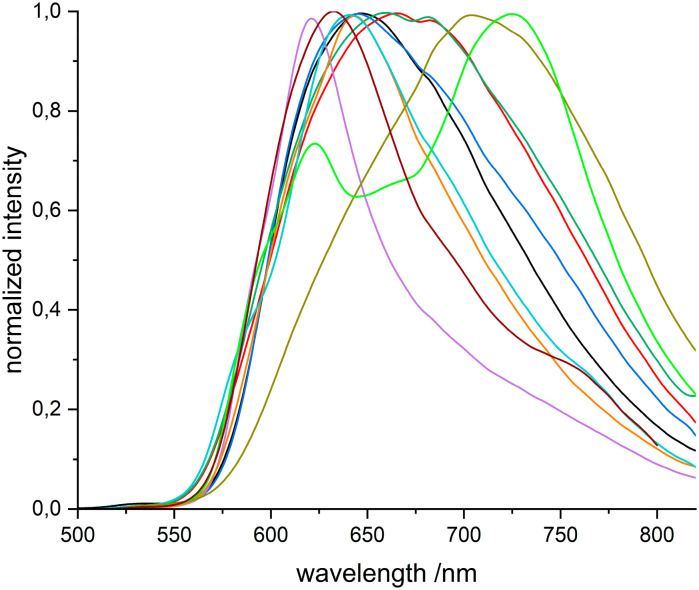
Normalized emission spectra of 1 (black), 2 (red), 3 (blue), 4 (dark green), 6 (purple), 7 (orange), 8 (turquoise), 10 (olive), 11 (light green), **L** (brown) as powder sample. *λ*_exc._ = 424 nm.

The solid-state PL spectra of coordination compounds 1–4, 7, 8, 10 and 11 exhibit a bathochromic shift of the emission maxima to varying extents compared to the uncoordinated ligand **L** (Fig. 1) at room temperature. 1, 3, 7 and 8 show the smallest bathochromic shift of their emission maxima from 632 nm (uncoordinated **L**) to 647 nm, 645 nm, 643 nm and 641 nm, respectively. Bigger shifts of the emission maxima are displayed by 2, 4 and 10 with emission maxima of 665 nm, 659 nm and 704 nm, respectively. Besides the global emission maximum at 725 nm, 11 exhibits also a local maximum at 623 nm which is slightly hypsochromically shifted compared to the uncoordinated ligand **L**. 6 also shows a hypsochromic shift of its emission maximum at 621 nm (Fig. 1).

### Crystal structures

All single crystals (SC) were obtained by vapor diffusion of an antisolvent (Et_2_O or 2-methoxy-2-methylpropane) into saturated solutions of the coordination compounds 1–4, 6–8, 10 and 11 in CH_3_CN (1, 3, 7, 10 and 11) or ace (2, 4, 6 and 8) at room temperature (RT).

**Fig. 2:**
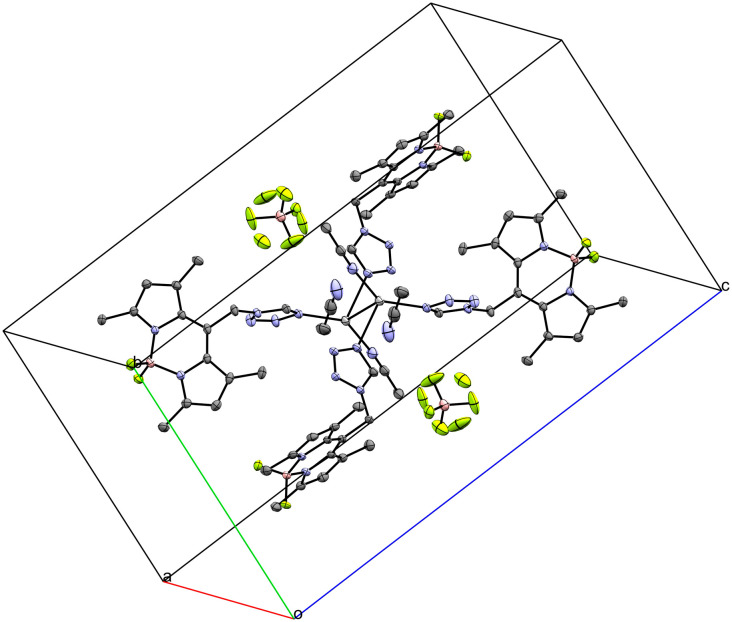
Structure of the coordination compound 1 (ellipsoids: 50% probability level; atom color code: grey – C, blue – N, light green – F, pink – B, light grey – Ag; H-atoms are omitted for clarity) with Ag–Ag interaction, disordered anion and crystal solvate (CH_3_CN).

Coordination compound 1 (Fig. 2) crystallizes in the monoclinic *P*2_1_/*n* space group, with one crystallographically unique dinuclear complex located on a center of inversion. Two CH_3_CN molecules and two non-coordinating counterions (BF_4_^−^) per complex are located on the general position. Moreover, the BF_4_^−^ anion is disordered about two positions with an occupancy ratio of 86.8 : 13.2(3), which can be interconverted by rotation along the B3–F5 axis. Each central atom is coordinated by three N-atoms, two tetrazolic *N4*-atoms (the italicized numbered N-atoms correspond to the tetrazole numbering system shown in Chart 1) and once by CH_3_CN (Fig. S1†). The bond distances for N13–Ag1, N12–Ag1 and N6–Ag1 are 2.184(2), 2.389(2) and 2.243(2) Å, respectively. The tetrazolic coordination bond distances are in line with corresponding literature examples (2.1842(16);^35^ 2.328(5) and 2.501(6);^36^ 2.241(5) and 2.264(5) Å^15^). The distance between the two Ag-atoms of the complex, which are related by inversion, is 3.2427(4) Å. This sub-van der Waals contact indicates ligand-unsupported argentophilic interactions,^10^ which are consistent with a comparable literature example (3.215–3.242 Å).^7^ Our preceding structural investigations on **L** have shown that the tz moiety can rotate almost freely around the N1–CH_2_ bond and therefore enables different conformers.^42^ The two crystallographically independent molecules of **L** in 1 are such conformers mainly differing in the orientation of the tz ring towards the FBF plane (22.9(2)° *vs.* 39.0(2)°).

**Fig. 3:**
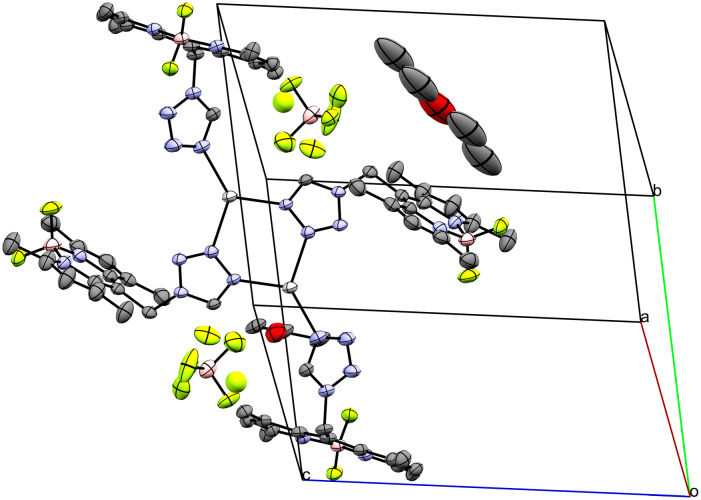
Structure of the coordination compound 2 (ellipsoids: 50% probability level; atom color code: grey – C, blue – N, light green – F, pink – B, light grey – Ag, red – oxygen; H-atoms are omitted for clarity) with bridging **L** and crystal solvates (ace and Et_2_O).

Coordination compound 2 (Fig. 3), synthesized in analogy to 1, but using a non-coordinating solvent (ace) instead of CH_3_CN, crystallizes in the triclinic *P*1̄ space group. 2 likewise shows a crystallographically unique dinuclear complex with one solvate molecule ace, one solvate molecule Et_2_O and two non-coordinating counterions (BF_4_^−^) per complex, which is located on a center of inversion. Moreover, the non-coordinating counterion (BF_4_^−^) is disordered in an analogous manner to 1, but with a different occupancy ratio of 75.7 : 24.3(13). The two Ag-atoms are bridged by two molecules of **L**, each with their tetrazolic *N3*- and *N4*-atom (Fig. 3 and S2†). Beside the two bridging ligands, each Ag-atom is also coordinated by another molecule of **L**, with its tetrazolic *N4*-atom. The bond distance between this monocoordinating ligand **L** and the Ag-atom (N12–Ag1) is rather short with 2.191(4) Å, but well within the range reported in literature.^35^ Interestingly, the distance between the *N3*-atom of the bridging ligand and the Ag-atom (N5–Ag1 2.251(4) Å) is shorter than the distance between the *N4*-atom and the same Ag-atom (N6(1 − *x*, 1 − *y*, −*z*)–Ag1 2.345(3) Å). Generally, it is assumed that the *N4*-atom of a tz ligand represents the preferred coordination site,^37^ which was already shown for Ag(i) in 1 and also in 2 for the monocoordinating ligand. Therefore, the *N4*–Ag(I) bond is expected to be shorter in a bridging coordination mode than the *N3*–Ag(I) bond.^37^

The bridging mode of **L** in 2 leads to a longer distance between the two Ag-atoms of the complex, which are related by inversion (Ag1(1 − *x*, 1 − *y*, −*z*)–Ag1 4.1294(5) Å), in comparison to 1. This is unexpected since short Ag(i)–Ag(i) distances are often promoted by bridging ligands.^2,5^ Again, there are two crystallographically independent molecules of **L** in 2 which are conformers. The monocoordinating ligand shows an tz/FBF angle of 42.8(5)°, whereas the bridging ligand shows an angle of 15.6(6)°. In both cases the tetrazolic *N2*-atom is facing towards the B-atom.

**Fig. 4:**
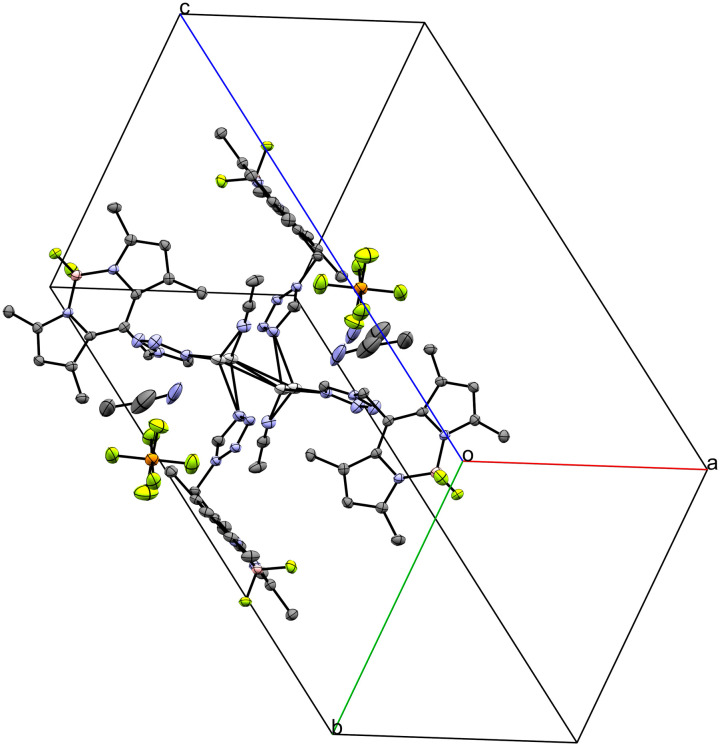
Structure of the coordination compound 3 (ellipsoids: 50% probability level; atom color code: grey – C, blue – N, light green – F, pink – B, light grey – Ag, orange – P; H-atoms are omitted for clarity) with Ag–Ag interaction, disordered metal centers and crystal solvate (CH_3_CN).

Coordination compound 3 (Fig. 4 and S3†) is structurally closely related to 1, with some noteworthy differences. First of all, the crystallographically unique Ag-atom is disordered about two positions with an occupancy ratio of 53.09 : 46.91(12), as well as the crystal solvate (CH_3_CN), where N14 and C33 are disordered with an occupancy ratio of 64.8 : 35.2(8). In contrast to the BF_4_^−^ molecule in 1, the octahedral PF_6_^−^ molecule shows no disorder in 3. The bond distances between the central atom and the tetrazolic *N4*-atom of the ligands **L** and also of the coordinating CH_3_CN molecule are very similar to 1 (N13–Ag1 2.223(3) Å, N12–Ag1 2.362(3) Å and N6–Ag1 2.290(3) Å). The disorder of the central atom allows up to three different Ag–Ag distances (Ag1(−*x*, 1 − *y*, 1 − *z*)–Ag1 2.6028(15) Å, Ag1(−*x*, 1 − *y*, 1 − *z*)[Ag1′(−*x*, 1 − *y*, 1 − *z*)]–Ag1′[Ag1] 3.0946(16) Å, Ag1′(−*x*, 1 − *y*, 1 − *z*)–Ag1′ 3.6073(17) Å) between the two Ag-atoms of the dinuclear complex which are related by inversion. The first two Ag–Ag distances are sub-van der Waals contacts, indicating argentophilic interactions^10^ like in 1. Similar to 1, there are two crystallographically independent molecules of **L** in 3, which are conformers (angle between FBF plane and tz: 26.8(3)° and 44.1(3)°).

Coordination compound 4, (Fig. S4–S6†) crystallizes in the triclinic *P*1̄ space group with one crystallographically unique mononuclear complex located on the general position. The anion (PF_6_^−^) is non-coordinating and also located on the general position. The coordination compound is not disordered and the crystal is free of any incorporated solvent molecules. The Ag-atom is coordinated in a pseudo trigonal planar geometry (N6–Ag1–N12 127.60(11)°; N12–Ag1–N18 140.41(12)°) by three molecules of **L**, each with the tetrazolic *N4*-atom. The BODIPY cores of two of the three ligands are located on one side of the plane which is defined by the trigonal planar geometry, whereas the third one points to the other side. The bond distances between the *N4*-atoms and the central atom consist of one short (Ag1–N12 2.184(4) Å) and two medium (Ag1–N6 2.305(3) Å, Ag1–N18 2.306(3) Å) long contacts. All three coordinating ligands are conformers of **L**, mainly differing in the orientation of their tz moieties in respect to the FBF plane (53.0(4)°, 55.9(5)° and 32.5(7)°), as it was already discussed earlier. In all three cases, the tetrazolic H-atom faces towards the B-atom.

**Fig. 5:**
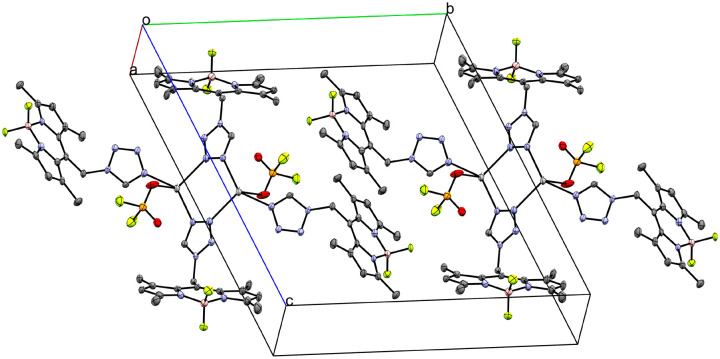
Crystal packing of dinuclear complex 5 (ellipsoids: 50% probability level; atom color code: grey – C, blue – N, light green – F, pink – B, light grey – Ag, red – O, orange – P; H-atoms are omitted for clarity).

In contrast to BF_4_^−^ and PF_6_^−^, PF_2_O_2_^−^ is acting as a coordinating anion in coordination compounds 5 and 6. 5 (Fig. 5 and S7†) is structurally related to 2, with two main differences. First, 5 crystallizes without any solvate molecules and each PF_2_O_2_^−^ is coordinated with one of its O-atoms to one Ag(i) center. Moreover, the crystallographically unique Ag-atom is coordinated by three tetrazolic N-atoms (two *N4*-atoms and one *N3*-atom) from three molecules of ligand **L**. One of these ligand molecules coordinates through a single site, while the other two function as bridging ligands, like in 2. The bond distance between the monocoordinating ligand **L** and the Ag-atom (N7–Ag1) is longer than in 2 with 2.246(4) Å, yet still in line with literature.^15^ In contrast to 2, the bond distance between the Ag-atom and the tetrazolic *N4*-atom (N1–Ag1 2.262(3) Å) is shorter than the distance between the Ag-atom and the tetrazolic *N3*-atom (N2(1 − *x*, 2 − *y*, 1 − *z*)–Ag1 2.376(3) Å) of the bridging ligand **L**. This is in good agreement with the already observed coordination behavior of the tz based ligand **L** and is consistent with literature^37^ (*vide supra*). The distance between the two Ag-atoms of the crystallographically unique dinuclear complex, which are related by inversion, in 5 is 4.0191(10) Å, slightly shorter than in 2 (4.1294(5) Å), however it is significantly longer than in 1 (3.2427(4) Å). The distance between the coordinating O-atom of the anion and the Ag-atom (O2–Ag1 2.522(3) Å) is very similar to a literature known Ag–OPF_2_O bond (2.5166(1) Å).^54^

In addition to the mentioned differences between 2 and 5, the spatial arrangement of the ligand **L** is slightly different, which is visualized in Fig. S8.† In 5, the two ligand molecules are two crystallographically independent molecules of **L**, which are conformers as in 2. In both cases the tetrazolic *N2*-atom points towards the FBF plane, but the angles between this plane and the tz plane vary: the bridging molecule of **L** shows 35.8(4)° and the monocoordinating ligand 24.1(4)°.

**Fig. 6:**
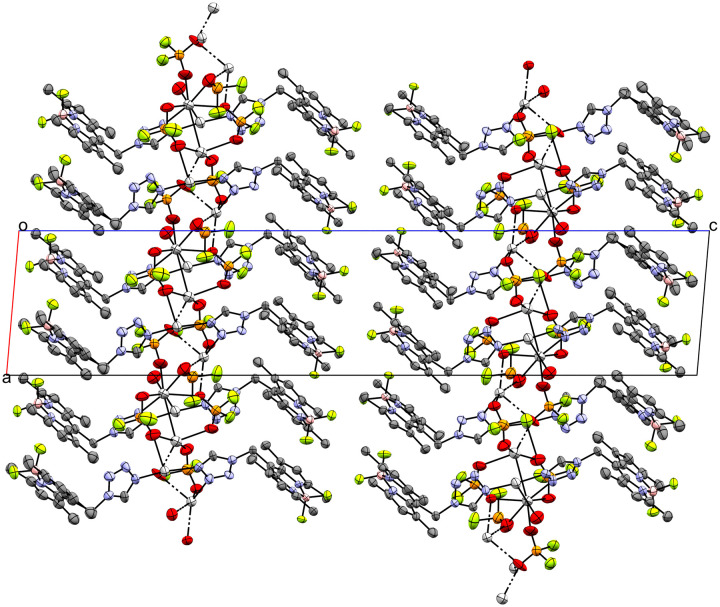
Crystal packing of coordination polymer 6 viewed along the crystallographic *b*-axis (ellipsoids: 50% probability level; atom color code: grey – C, blue – N, light green – F, pink – B, light grey – Ag, red – O, orange – P; H-atoms are omitted for clarity).

**Fig. 7:**
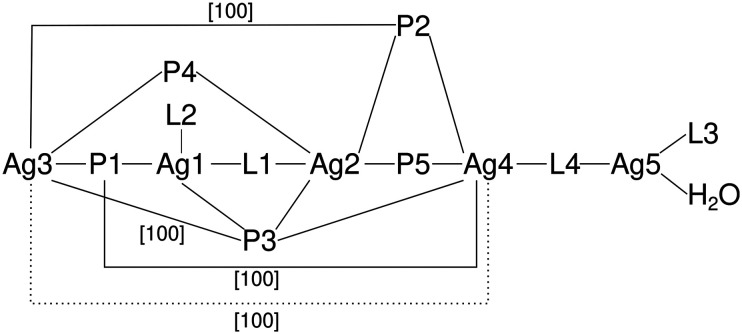
Connectivity graph of coordination compound 6 (L – ligand (**L**), P – PF_2_O_2_^−^, [100] – connection through translation along the crystallographic *a*-axis, dotted line – short Ag–Ag contact).

Coordination compound 6, a 1D coordination polymer, crystallizes in the triclinic *P*1̄ space group and extends along the crystallographic *a*-axis (Fig. 6). The infinite chain is symmetric only by translation. Besides five crystallographically independent Ag-atoms, the asymmetric unit consists of five anion molecules (PF_2_O_2_^−^), four conformers of **L** (two monocoordinating and two bridging ligands) and one molecule of water (Fig. S9–S11†). All those molecules are coordinated to at least one Ag-atom. Fig. 7 displays the remarkably complex connectivity graph of coordination compound 6. Before discussing the connectivity of each metal center, two significant structural properties should be noted: first, the Ag5-atom is not part of the chain itself (*vide infra*). Second, the P3F_2_O_2_^−^ molecule coordinates to all metal atoms (Ag1–Ag4) of the infinite chain.

The Ag1-atom is coordinated by two molecules of **L** (L1 and L2 in Fig. 7) each with the tetrazolic *N4*-atom (N6–Ag1 2.221(13) Å and N12–Ag1 2.188(13) Å) and two O-atoms of two different PF_2_O_2_^−^ molecules (P1F_2_O_2_^−^: O1–Ag1 2.588(13) Å and P3(−1 + *x*, *y*, *z*)F_2_O_2_^−^: O5(−1 + *x*, *y*, *z*)–Ag1 2.669(15) Å). Moreover, of the two neighboring Ag-atoms (Ag2–Ag1 3.8354(19) Å and Ag3–Ag1 3.351(2) Å), only the second contact is short enough to indicate intermetallic interactions. Interestingly, as already noted for 2, a bridging tz unit may be detrimental for the formation of shorter Ag–Ag distances.

Ag2 is coordinated by four O-atoms (O4(−1 + *x*, *y*, *z*)–Ag2 2.511(10) Å, O5(−1 + *x*, *y*, *z*)–Ag2 2.696(13) Å, O8–Ag2 2.354(10) Å and O9(−1 + *x*, *y*, *z*)–Ag2 2.517(12) Å) of four different PF_2_O_2_^−^ molecules and one tetrazolic *N3*-atom (N5–Ag2 2.361(13) Å) of the ligand **L** (L1 in Fig. 7) bridging *via* N6 to Ag1. Beside the already discussed interaction of Ag2 with Ag1, Ag2 has another interaction with Ag4(−1 + *x*, *y*, *z*) (3.4770(18) Å). However, the distances to Ag3 and Ag5(−1 + *x*, *y*, *z*) are with 3.9329(18) and 4.7529(19) Å, respectively, too long for any interaction.

Ag3 is, like Ag2, coordinated by four different O-atoms (O1–Ag3 2.314(13) Å, O3–Ag3 2.367(13) Å, O5(−1 + *x*, *y*, *z*)–Ag3 2.366(13) Å and O7–Ag3 2.387(14) Å) and it is the only Ag-atom in 6 which is not coordinated by any **L** molecule. In addition to the already discussed proximity of Ag3 to Ag1 and Ag2, there are two more neighboring Ag-atoms: Ag4 and Ag5. The distance between Ag3 and Ag5 is rather long with 3.860(2) Å, however the Ag4-atom is in close proximity to Ag3 and shows the strongest intermetallic interaction in 6 with a distance of 3.1140(18) Å.

Ag4 is coordinated by four different PF_2_O_2_^−^ molecules (O2–Ag4 2.461(15) Å, O4–Ag4 2.509(13) Å, O6–Ag4 2.421(12) Å, O9–Ag4 2.491(12) Å) and once by the tetrazolic *N3*-atom of a molecule of **L** (L4 in Fig. 7, N23–Ag4 2.385(14) Å), which bridges to Ag5 with its tetrazolic *N4*-atom. The remaining Ag–Ag contact of Ag4, which was not yet discussed, is the Ag4–Ag5 contact with a distance of 3.579(2) Å.

Ag5 has a coordination number of only three and therefore the smallest of all metal centers in 6. It is coordinated by two tetrazolic N4-atoms of two different molecules of **L** (L3 and L4 in Fig. 7, N18–Ag5 2.169(13) Å and N24–Ag5 2.163(13) Å), however, only the second one bridges to the polymeric chain (Ag4). Additionally, Ag5 is coordinated once by water (OW1–Ag5 2.689(14) Å).

All three coordination compounds containing perchlorate as anion (7–9) show structures very similar to already described coordination compounds and are not discussed in detail. 7 is isostructural to 1, but the ClO_4_^−^ molecule shows no disorder in contrast to the BF_4_^−^ molecule (Fig. S12†). 8 is isostructural to 5 with minor deviations in the spatial arrangement of the anions (ClO_4_^−^*vs.* PF_2_O_2_^−^) (Fig. S13†). Coordination compound 9 is isostructural to coordination polymer 6.

**Fig. 8:**
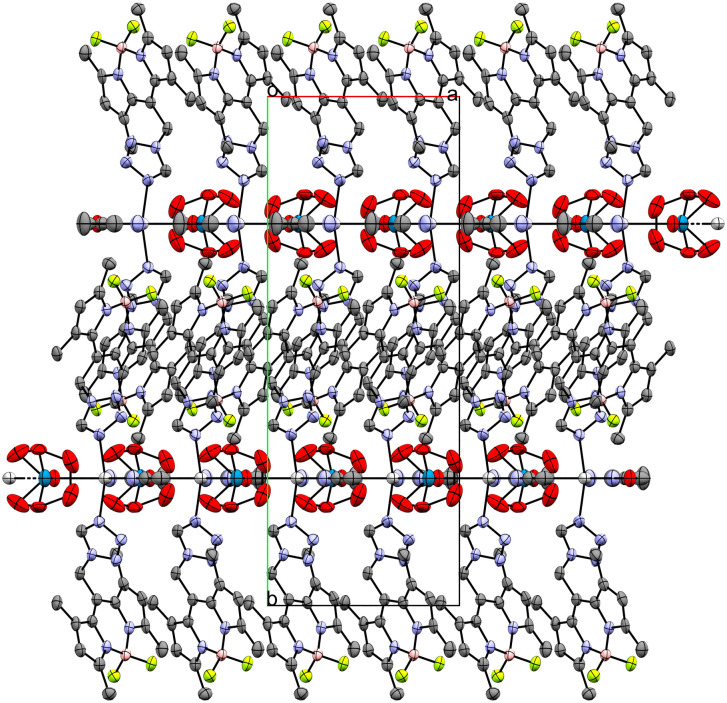
Crystal packing of coordination polymer 10 with CH_3_CN as solvate viewed along the crystallographic *c*-axis (ellipsoids: 50% probability level; atom color code: grey – C, blue – N, light green – F, pink – B, light grey – Ag, red – O, petrol blue – Re; H-atoms are omitted for clarity).

Coordination compound 10 crystallizes as coordination polymer in the orthorhombic space group *Pnma* extending along the crystallographic *a*-axis (Fig. 8). The infinite cation is symmetric by the 2_1_ screw rotation in [100] direction, the *m*_[010]_ reflection and the *a*_[001]_ glide reflection of the *Pnma* space group. The asymmetric unit of 10 (Fig. S14†) consists of one Ag-atom, one molecule of **L**, two molecules of CH_3_CN and a perrhenate anion. The crystallographically unique Ag-atom is coordinated by two tetrazolic *N4*-atoms (N6/N6(*x*, 1/2 − *y*, *z*)–Ag1 2.298(5) Å) of two molecules of **L** in a monocoordinating fashion. Moreover, one molecule of CH_3_CN is coordinated to the central atom (N7–Ag1 2.248(10) Å), whereas the other one is embedded as solvate. The ReO_4_^−^ counterion coordinates with its O1-atom to two symmetry related Ag-atoms: the Ag1-atom (2.520(7) Å) and the Ag1(1/2 + *x*, 1/2 − *y*, 3/2 − *z*)-atom (2.709(8) Å) (Fig. S15†). The ReO–Ag distances are consistent with literature (2.397(2)–2.894(3) Å).^29^

The ReO_4_^−^ molecule is disordered about the *m*_[010]_ reflection plane of the *Pnma* space group. Overall, the Ag1-atom has a coordination number of five with a distorted square pyramidal geometry.

**Fig. 9:**
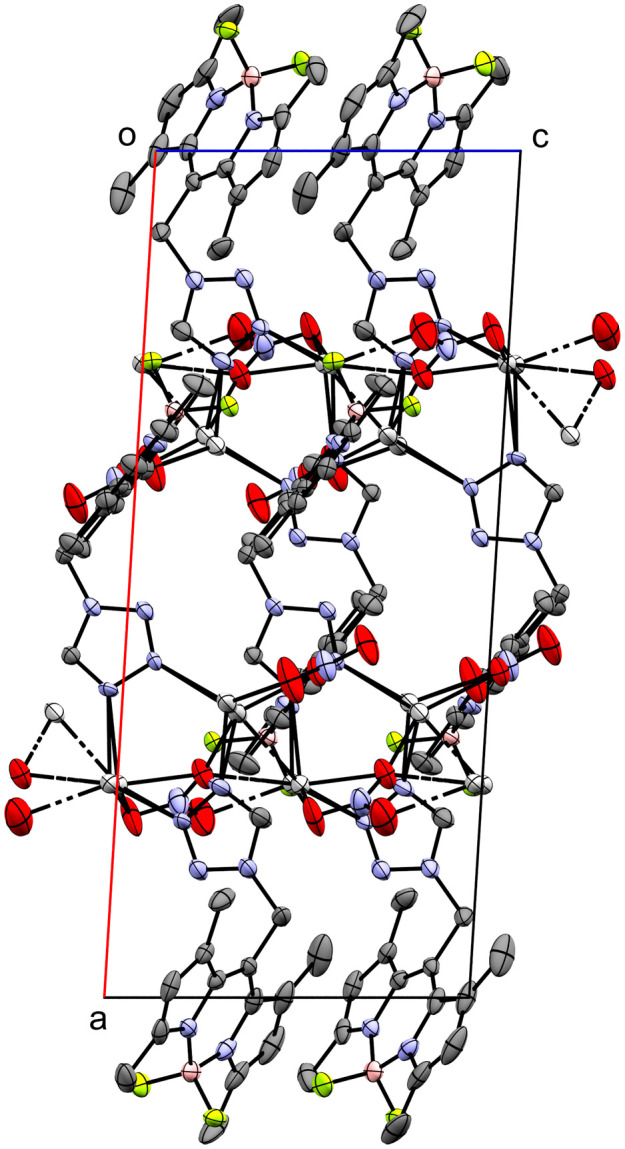
Crystal packing of coordination polymer 11 viewed along the crystallographic *b*-axis (ellipsoids: 50% probability level; atom color code: grey – C, blue – N, light green – F, pink – B, light grey – Ag, red – O; H-atoms are omitted for clarity).

11, the only coordination compound discussed in this work with NO_3_^−^ as counterion, crystallizes as coordination polymer (Fig. 9) in the monoclinic *P*2_1_/*c* space group extending along the crystallographic *c*-axis. The infinite chain is located on the *c*_[010]_ glide reflection plane. Both crystallographically independent Ag-atoms are disordered about two positions with an occupancy ratio of 97.20 : 2.80(12) and coordinated by two molecules of **L** in a bridging mode *via* the tetrazolic *N3* and *N4*-atoms (N5–Ag1 2.222(5), N12–Ag1 2.301(5), N6–Ag2 2.354(4), N11–Ag2 2.301(5)) (Fig. S16†). The distance between the major positions of the two Ag-atoms is 3.6221(7) Å, significantly shorter compared to the equivalent distance of the bridged dimers 2, 5 and 8 (>4 Å). The metal centers are also coordinated each by one NO_3_^−^ molecule. In the case of Ag1, the anion molecule functions as a bidentate ligand (O1–Ag1 2.417(6) Å, O2–Ag1 2.412(5) Å). For Ag2, the case is more complicated since the N14O_3_^−^ molecule coordinates once with its O4-atom (O4–Ag2 2.463(6) Å) and the N14O_3_^−^ molecule, which is related by glide reflection, twice (O5(*x*, 3/2 − *y*, 1/2 + *z*)–Ag2 2.558(5) Å, O6(*x*, 3/2 − *y*, 1/2 + *z*)–Ag2 2.508(7) Å). There are two additional interesting interactions: first a close intermetallic interaction of Ag1′ with Ag2′(*x*, 3/2 − *y*, −1/2 + *z*) (3.06(3) Å) and second an anagostic interaction of the tetrazolic H15 with the Ag1′(*x*, 3/2 − *y*, 1/2 + *z*)-atom (2.44 Å) (Fig. S17†). The unit cell contains two distinct chains related by a 2_1_ screw rotation.

## Conclusion

In this study the versatile coordination behavior of ligand **L** towards Ag(i) was demonstrated. In addition to the monocoordinating mode involving the tetrazolic *N4*-atom (1, 3, 4, 7 and 10), a bridging coordination mode utilizing both the tetrazolic *N3*- and *N4*-atoms (2, 5, 6, 8, 9 and 11) was observed. Indeed, some examples (2, 5, 6, 8 and 9) display both the monocoordinating and the bridging mode within the same coordination compound. The bridging coordination mode does not inherently result in short Ag–Ag interactions in Ag bridging scenarios (2, 5 and 8) in contrast to the literature. However, the addition of a co-ligand (CH_3_CN) inhibits such bridging and results in shorter intermetallic distances (1: 3.2427(4) Å and 7: 3.2213(4) Å). Even shorter Ag–Ag interactions (3.1140(18) Å and 3.06(3) Å) were found when the compounds formed CPs (6, (9) and 11). In addition to the bridging mode of **L** and Ag–Ag interactions, interconnecting bonds in these CPs were formed by multitopic anions (PF_2_O_2_^−^, ClO_4_^−^, ReO_4_^−^, NO_3_^−^).

Not only do the geometric differences (*T*_d_, *D*_3h_ and *O*_h_) of the anions result in distinct structures, but geometrically identical anions (BF_4_^−^, PF_2_O_2_^−^, ClO_4_^−^, ReO_4_^−^) also form different structures due to variations in their donor abilities. BF_4_^−^ exhibits no coordination (1, 2), while PF_2_O_2_^−^ and ClO_4_^−^ can coordinate to the metal center forming similar structures (5*vs.*8 and 6*vs.*9) and ReO_4_^−^ functions as a bridging anion (10).

The strong PL properties of **L** in the solid state were found to be retained after complexation. However, the emission maxima of all investigated coordination compounds are significantly shifted. The differences in emission wavelength are primarily attributed to structural variations of the coordination compounds, as structurally similar coordination compounds (1, 3 and 7), possessing different anions, exhibit comparable emission maxima (647 nm, 645 nm, 643 nm).

Regarding the PL properties, future studies will focus on solid state quantum yield measurements and in-depth investigations into the mechanisms underlying the differences in emission maxima compared to the uncoordinated ligand **L**. To complete the structure–property relationship for these investigations, the selective bulk preparation of coordination compounds 5 and 9, which were identified as side products during crystallization, is highly important. Finally, this study highlights the potential of **L** as a photoluminescence-active ligand, suggesting that coordination compounds of **L** and its analogues^42^ with various metal centers could lead to greater structural diversity and new structure–property relationships.

## Data availability

The data supporting this article have been included as part of the ESI.† Crystallographic data for 1–11 has been deposited at the CCDC under 2409394–2409399 and 2409407–2409411.

## Conflicts of interest

There are no conflicts to declare.

## Supplementary Material

CE-027-D5CE00197H-s001

CE-027-D5CE00197H-s002
