# Supplementary material for: Structural tuning of tetrazole-BODIPY Ag(i) coordination compounds via co-ligand addition and counterion variation
Source: CrystEngComm. 2025 Mar 20;27(17):2689–97. doi: 10.1039/d5ce00197h (PMC11962859; doi:10.1039/d5ce00197h)
Supplement: CE-027-D5CE00197H-s001 [file CE-027-D5CE00197H-s001.pdf]

Supplementary Information (SI) for CrystEngComm.
This journal is © The Royal Society of Chemistry 2025

Supporting Information

Structural Tuning of Tetrazole-BODIPY Ag(I) Coordination Compounds via Co-Ligand Addition and Counterion Variation

Matthias Schöbinger,+*[a] Martin Huber,+[a] Berthold Stöger,[b] Christian Hametner[a] and Peter Weinberger*[a]

[a]

M. Schöbinger, M. Huber, Prof. C. Hametner, Prof. P. Weinberger
Institute of Applied Synthetic Chemistry, TU Wien
Getreidemarkt 9, 1060, Vienna (Austria)
E-mail: matthias.schoebinger@tuwien.ac.at, peter.e163.weinberger@tuwien.ac.at

[b]

Dr. B. Stöger
X-ray Center, TU Wien
Getreidemarkt 9/164, 1060, Vienna (Austria)

[+]

These authors contributed equally to this manuscript.

Table of contents

Experimental protocols 3
X-Ray structure determination 4
PXRD measurements 10
UV/Vis and PL measurements 15
Diffuse reflectance and emission spectra 15
NMR measurements 20
NMR spectra 20
ATR-IR measurements 41
IR spectra 41
References 46

Experimental protocols

All syntheses were performed with dried glassware, anhydrous and degassed solvents, and under an atmosphere of argon. Reaction monitoring was performed by TLC, if not otherwise noted. The ligand (4,4-difluoro-1,3,5,7-tetramethyl-8-[(*1H*-tetrazol-1-yl)methyl]-4-bora-3a,4a-diaza-s-indacene) **L** was synthesized according to a literature protocol[1], all other starting materials were commercially purchased and used without further purifications if not stated otherwise.

Synthesis of the complexes

For synthesis of the silver coordination compounds **1-4, 6-8**, **10**, **11** the corresponding AgX salt (X = BF_4 for **1**, **2**, PF_6 for **3**, **4**, PF_2O_2 for **6**, ClO_4 for **7**, **8**, ReO_4 for **10**, NO_3 for **11**) (0.0757 mmol, 1.00 equiv.), ligand **L** (50.0 mg, 0.151 mmol, 2.00 equiv.) and solvent (acetone for **2**, **4**, **6** and **8**; acetonitrile for **1**, **3**, **7**, **10** and **11**) (3 mL) were combined in a Schlenk tube and stirred at 40 °C overnight. Two thirds of the solvent were evaporated and diethyl ether (8 mL) was added to precipitate a red colored compound. The solvent was removed, the solid residue was washed 3 times with diethyl ether (6 mL) and dried in high vacuum.

1

^{1}H-NMR (600 MHz, $C_6D_5NO_2$): δ = 9.83 (s, 1H), 6.23 (s, 2H), 6.05 (s, 2H), 2.53 (s, 6H), 2.40 (s, 6H), 2.12 (s, 2H) ppm. ^{13}C{^{1}H}-NMR (151 MHz, $C_6D_5NO_2$): δ = 158.3, 145.1, 142.0, 132.8, 45.5, 15.6, 14.7 ppm. ^{19}F-NMR (565 MHz, $C_6D_5NO_2$): δ = -145.0 (q, *J* = 30.9 Hz), -150.0 ppm. ^{11}B{^{1}H}-NMR (193 MHz, $C_6D_5NO_2$): δ = 0.6 (t, *J* = 32.4 Hz), 0.4 ppm.

2

^{1}H-NMR (600 MHz, $C_6D_5NO_2$): δ = 9.81 (s, 1H), 6.22 (s, 2H), 6.05 (s, 2H), 2.53 (s, 6H), 2.40 (s, 6H) ppm. ^{13}C{^{1}H}-NMR (151 MHz, $C_6D_5NO_2$): δ = 158.3, 145.1, 142.0, 132.8, 45.4, 15.6, 14.7 ppm. ^{19}F-NMR (565 MHz, $C_6D_5NO_2$): δ = -145.0 (q, *J* = 31.0 Hz), -150.0 ppm. ^{11}B{^{1}H}-NMR (193 MHz, $C_6D_5NO_2$): δ = 0.6 (t, *J* = 32.3 Hz), 0.4 ppm ppm.

3

^{1}H-NMR (600 MHz, $C_6D_5NO_2$): δ = 9.74 (s, 1H), 6.22 (s, 2H), 6.05 (s, 2H), 2.52 (s, 6H), 2.39 (s, 6H), 2.12 ppm (s, 1H). ^{13}C{^{1}H}-NMR (151 MHz, $C_6D_5NO_2$): δ = 158.4, 144.9, 142.0, 132.7, 45.4, 15.6, 14.7 ppm. ^{19}F-NMR (565 MHz, $C_6D_5NO_2$): δ = -71.1 (d, *J* = 711.0 Hz), -145.0 ppm (q, *J* = 31.2 Hz). ^{11}B{^{1}H}-NMR (193 MHz, $C_6D_5NO_2$): δ = 0.6 ppm (t, *J* = 32.3 Hz). ^{31}P{^{1}H}-NMR (243 MHz, $C_6D_5NO_2$): δ = -143.0 ppm (sept, *J* = 711.0 Hz).

4

^{1}H-NMR (600 MHz, $C_6D_5NO_2$): δ = 9.74 (s, 1H), 6.22 (s, 2H), 6.05 (s, 2H), 2.52 (s, 6H), 2.39 ppm (s, 6H). ^{13}C{^{1}H}-NMR (151 MHz, $C_6D_5NO_2$): δ = 158.4, 144.9, 142.0, 132.7, 45.4, 15.6, 14.7 ppm. ^{19}F-NMR (565 MHz, $C_6D_5NO_2$): δ = -71.1 (d, *J* = 711.1 Hz), -145.0 ppm (q, *J* = 31.3 Hz). ^{11}B{^{1}H}-NMR (193 MHz, $C_6D_5NO_2$): δ = 0.6 ppm (t, *J* = 32.2 Hz). ^{31}P{^{1}H}-NMR (243 MHz, $C_6D_5NO_2$): δ = -143.0 ppm (sept, *J* = 710.4 Hz).

6

^{1}H-NMR (600 MHz, $C_6D_5NO_2$): δ = 9.58 (s, 1H), 6.15 (s, 2H), 6.05 (s, 2H), 2.54 (s, 6H), 2.36 ppm (s, 6H). ^{13}C{^{1}H}-NMR (151 MHz, $C_6D_5NO_2$): δ = 158.2, 144.4, 142.0, 132.8, 45.0, 15.6, 14.7 ppm. ^{19}F-NMR (565 MHz, $C_6D_5NO_2$): δ = -82.5 (d, *J* = 962.1 Hz), -145.0 ppm (q, *J* = 31.4 Hz). ^{11}B{^{1}H}-NMR (193 MHz, $C_6D_5NO_2$): δ = 0.6 ppm (t, *J* = 32.3 Hz). ^{31}P{^{1}H}-NMR (243 MHz, $C_6D_5NO_2$): δ = -14.2 ppm (t, *J* = 963.7 Hz).

7

^{1}H-NMR (600 MHz, $C_6D_5NO_2$): δ = 9.83 (s, 1H), 6.22 (s, 2H), 6.05 (s, 2H), 2.53 (s, 6H), 2.40 (s, 6H), 2.12 ppm (s, 3H). ^{13}C{^{1}H}-NMR (151 MHz, $C_6D_5NO_2$): δ = 158.3, 145.1, 142.0, 132.8, 45.4, 15.7, 14.7 ppm. ^{19}F-NMR (565 MHz, $C_6D_5NO_2$): δ = -145.0 ppm (q, *J* = 31.0 Hz). ^{11}B{^{1}H}-NMR (193 MHz, $C_6D_5NO_2$): δ = 0.6 ppm (t, *J* = 32.2 Hz).

8

^{1}H-NMR (600 MHz, $C_6D_5NO_2$): δ = 9.92 (s, 1H), 6.24 (s, 2H), 6.05 (s, 2H), 2.53 (s, 6H), 2.41 ppm (s, 6H). ^{13}C{^{1}H}-NMR (151 MHz, $C_6D_5NO_2$): δ = 158.4, 145.2, 142.0, 132.8, 45.5, 15.6, 14.7 ppm. ^{19}F-NMR (565 MHz, $C_6D_5NO_2$): δ = -145.0 ppm (q, *J* = 30.8 Hz). ^{11}B{^{1}H}-NMR (193 MHz, $C_6D_5NO_2$): δ = 0.6 ppm (t, *J* = 32.3 Hz).

10

^{1}H-NMR (600 MHz, $C_6D_5NO_2$): δ = 9.35 (s, 1H), 6.11 (s, 2H), 6.04 (s, 2H), 2.54 (s, 6H), 2.34 (s, 6H), 2.05 ppm (s, 1H). ^{13}C{^{1}H}-NMR (151 MHz, $C_6D_5NO_2$): δ = 158.1, 143.7, 142.0, 132.8, 44.7, 15.6, 14.7 ppm. ^{19}F-NMR (565 MHz, $C_6D_5NO_2$): δ = -145.1 ppm (q, *J* = 31.7 Hz). ^{11}B{^{1}H}-NMR (193 MHz, $C_6D_5NO_2$): δ = 0.6 ppm (t, *J* = 32.3 Hz).

11

^{1}H-NMR (600 MHz, $C_6D_5NO_2$): δ = 9.30 (s, 1H), 6.10 (s, 2H), 6.04 (s, 2H), 2.55 (s, 6H), 2.34 ppm (s, 6H). ^{13}C{^{1}H}-NMR (151 MHz, $C_6D_5NO_2$): δ = 158.1, 143.6, 142.0, 132.8, 44.6, 15.5, 14.7 ppm. ^{19}F-NMR (565 MHz, $C_6D_5NO_2$): δ = -145.1 ppm (q, *J* = 31.9 Hz). ^{11}B{^{1}H}-NMR (193 MHz, $C_6D_5NO_2$): δ = 0.7 ppm (t, *J* = 32.3 Hz).

X-Ray structure determination

X-ray diffraction data of solvates or non-solvates of **1**–**4** (CSD 2409394–2409397), **6** (CSD 2409399), **7**–**11** (CSD 2409407–2409411) were collected at T = 100 K (**1**–**4**, **6**, **7**, **10**, **11**), 150 K (**9**) or 200 K (**8**) in a dry stream of nitrogen on a STOE STADIVARI diffractometer system equipped with a Dectris Eiger CdTe hybrid photon counting detector using Cu-*K*α radiation (λ = 1.54186 Å). Data were reduced with X-Area (X-Area 1.31.192.0, LANA 2.7.12; STOE & Cie GmbH, Darmstadt, Germany, 2023). An absorption correction was applied with the multi-scan approach implemented in LANA. Intensity data of **5** (CSD 2409398) were collected at T = 100 K on a Bruker Kappa APEX II diffractometer system equipped with a CCD detector. Data were processed using the APEX3 software suite, reduced with SAINT-Plus and corrected for absorption effects using the multi-scan approach implemented in SADABS (Bruker computer programs: APEX3, SAINT and SADABS; Bruker AXS Inc., Madison, WI, 2020). All structures were solved by the dual-space approach implemented in SHELXT[2] and refined against F^2 with SHELXL.[3]

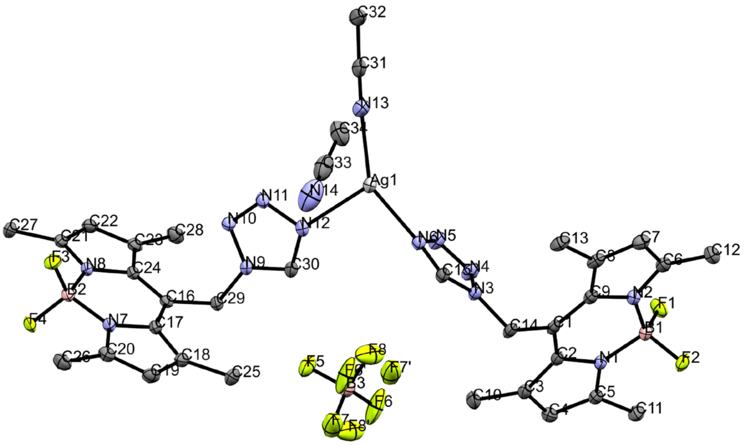

Figure S 1. Asymmetric unit of coordination compound **1** with atomic number labeling (ellipsoids: 50 % probability level; atom color code: grey…C, blue…N, light green…F, pink…B, light grey…Ag; H-atoms are omitted for clarity)

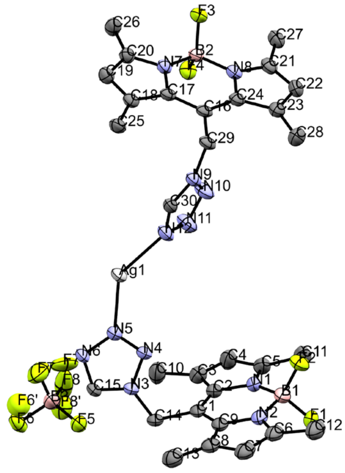

Figure S 2: Asymmetric unit of coordination compound **2** with atomic number labeling (ellipsoids: 50 % probability level; atom color code: grey…C, blue…N, light green…F, pink…B, light grey…Ag; H-atoms and crystal solvates (ace and Et_2O) are omitted for clarity)

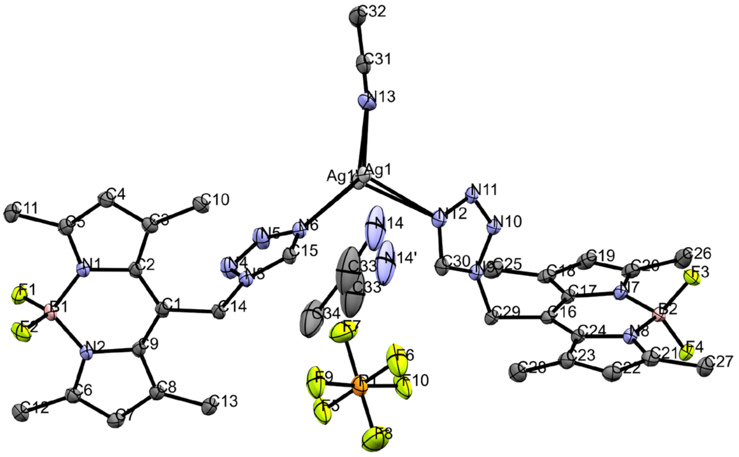

Figure S 3. Asymmetric unit of coordination compound **3** with atomic number labeling (ellipsoids: 50 % probability level; atom color code: grey…C, blue…N, light green…F, pink…B, light grey…Ag, orange…P; H-atoms are omitted for clarity)

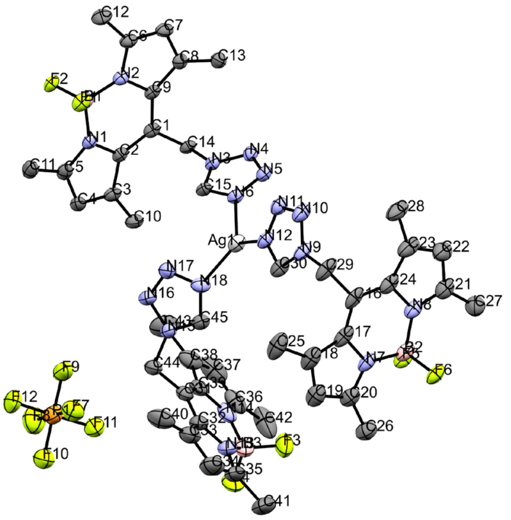

Figure S 4. Asymmetric unit of coordination compound **4** with atomic number labeling (ellipsoids: 50 % probability level; atom color code: grey…C, blue…N, light green…F, pink…B, light grey…Ag, orange…P; H-atoms are omitted for clarity)

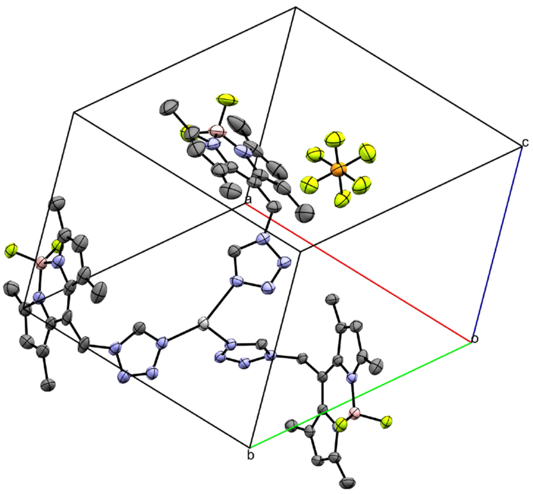

Figure S 5. Mononuclear coordination compound **4** (ellipsoids: 50 % probability level; atom color code: grey…C, blue…N, light green…F, pink…B, light grey…Ag, orange…P; H-atoms are omitted for clarity).

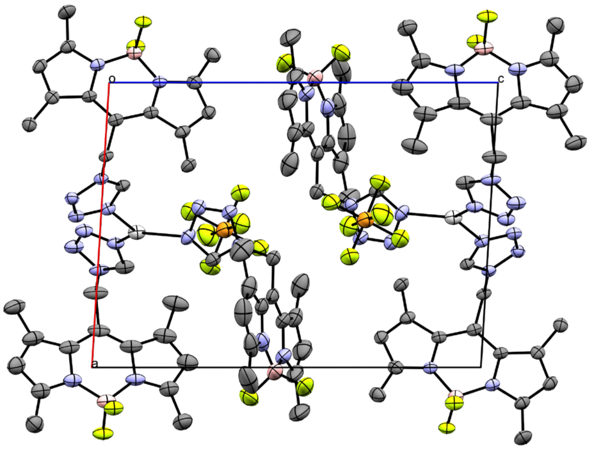

Figure S 6. Crystal packing of coordination compound **4** viewed along the crystallographic *b*-axis (ellipsoids: 50 % probability level; atom color code: grey…C, blue…N, light green…F, pink…B, light grey…Ag, orange…P; H-atoms are omitted for clarity).

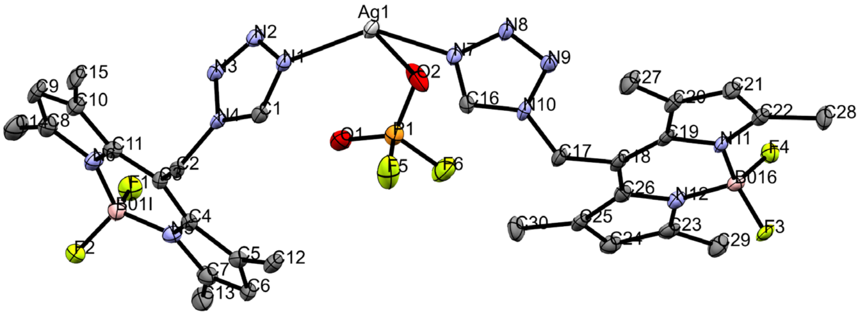

Figure S 7. Asymmetric unit of coordination compound **5** with atomic number labeling (ellipsoids: 50 % probability level; atom color code: grey…C, blue…N, light green…F, pink…B, light grey…Ag, red…oxygen, orange…P; H-atoms are omitted for clarity)

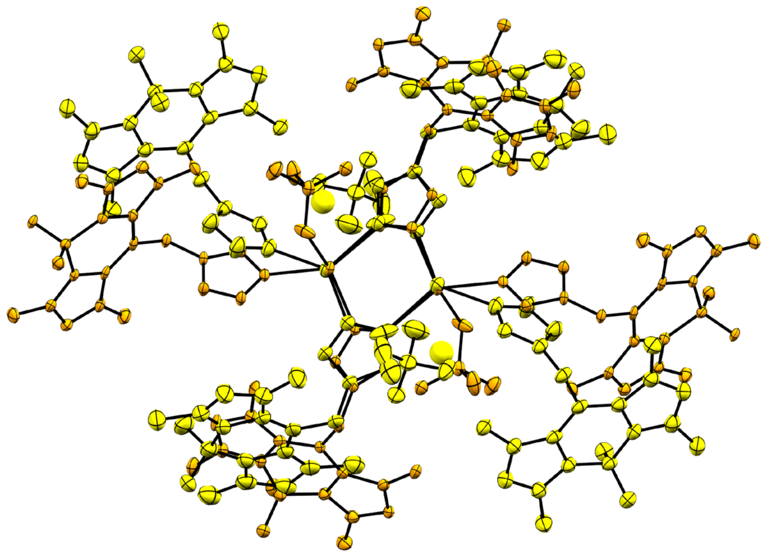

Figure S 8. Structure comparison of coordination compound **2** (yellow) and **5** (orange) (ellipsoids: 50 % probability level; H-atoms and crystal solvates are omitted for clarity)

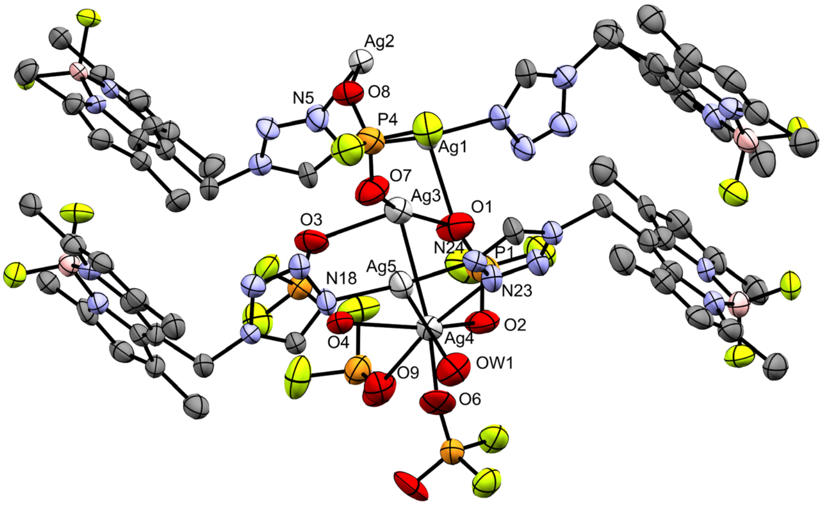

Figure S 9. Asymmetric unit of coordination compound **6** with atomic number labeling of the central atoms and coordinating atoms (ellipsoids: 50 % probability level; atom color code: grey…C, blue…N, light green…F, pink…B, light grey…Ag, red…oxygen, orange…P; H-atoms are omitted for clarity)

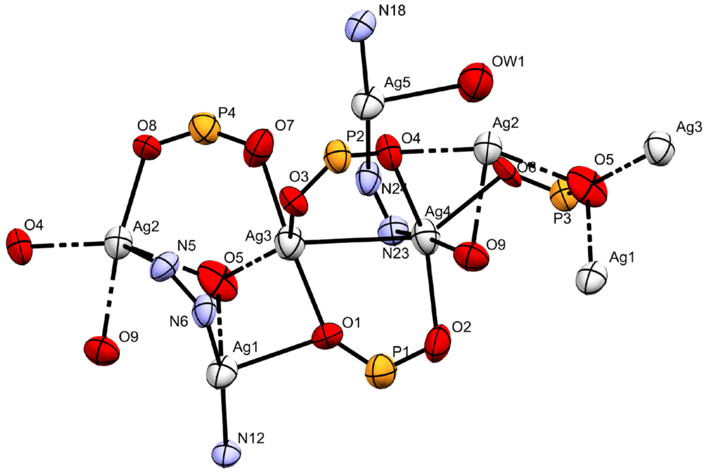

Figure S 10. Coordination environment of coordination compound **6** with atomic number labeling (ellipsoids: 50 % probability level; atom color code: grey…C, blue…N, light green…F, pink…B, light grey…Ag, red…oxygen, orange…P; H-atoms, non-coordinating and non-bridging atoms are omitted for clarity)

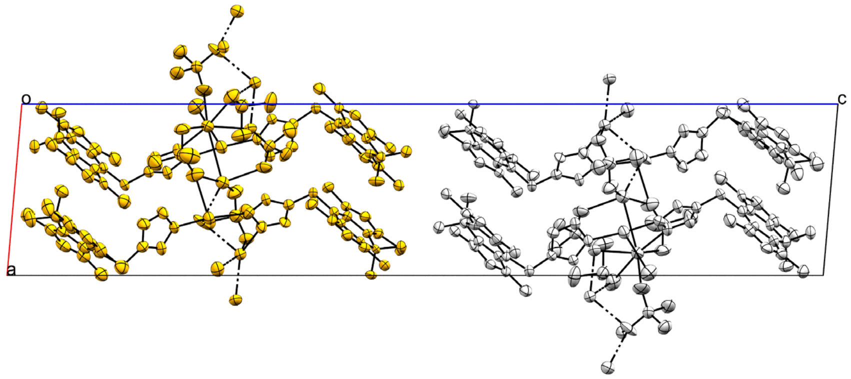

Figure S 11. Crystal packing of coordination compound **6** (ellipsoids: 50 % probability level; color by symmetry operation: light grey…identity, orange…inversion; H-atoms are omitted for clarity)

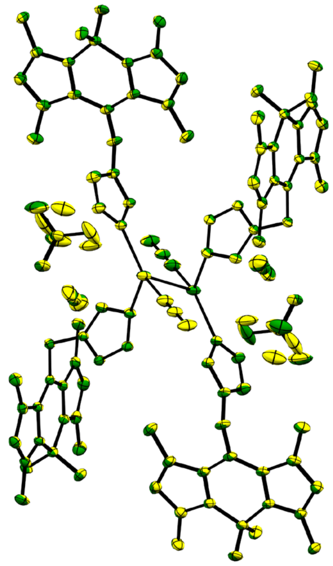

Figure S 12. Structure comparison of coordination compound **1** (yellow) and **7** (green) (ellipsoids: 50 % probability level; H-atoms omitted for clarity)

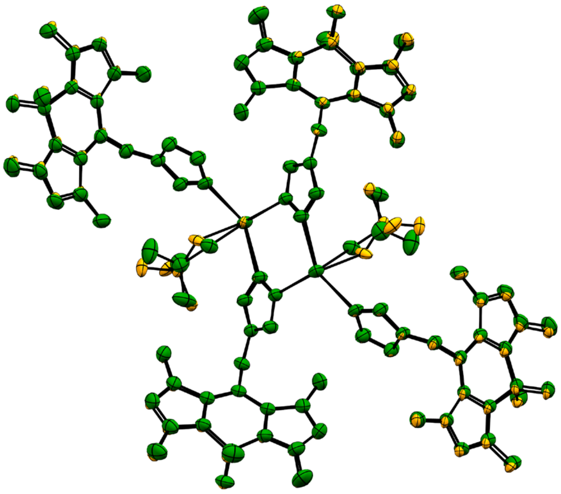

Figure S 13. Structure comparison of coordination compound **5** (orange) and **8** (green) (ellipsoids: 50 % probability level; H-atoms omitted for clarity)

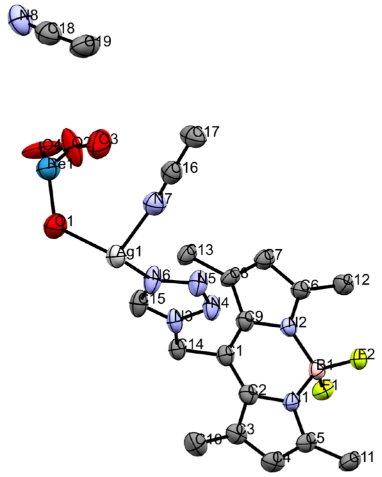

Figure S 14. Asymmetric unit of coordination compound **10** with atomic number labeling (ellipsoids: 50 % probability level; atom color code: grey…C, blue…N, light green…F, pink…B, light grey…Ag, red…oxygen, petrol blue…Re; H-atoms are omitted for clarity)

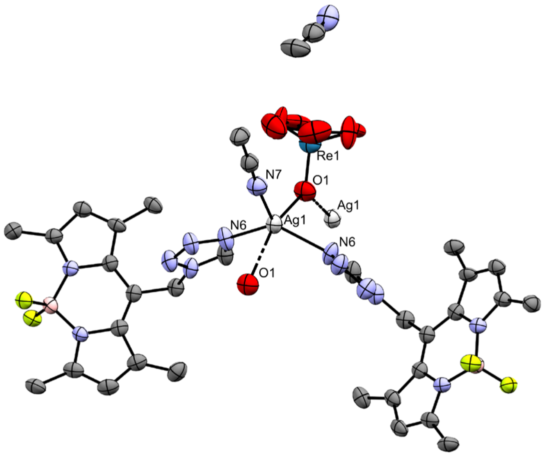

Figure S 15. Coordination compound **10** with atomic number labeling of selected atoms (ellipsoids: 50 % probability level; atom color code: grey…C, blue…N, light green…F, pink…B, light grey…Ag, red…oxygen, petrol blue…Re; H-atoms are omitted for clarity)

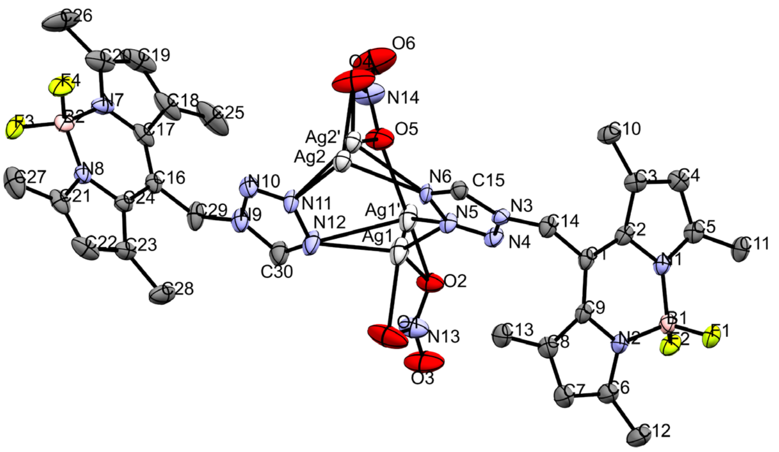

Figure S 16. Asymmetric unit of coordination compound **11** with atomic number labeling (ellipsoids: 50 % probability level; atom color code: grey…C, blue…N, light green…F, pink…B, light grey…Ag, red…oxygen; H-atoms are omitted for clarity)

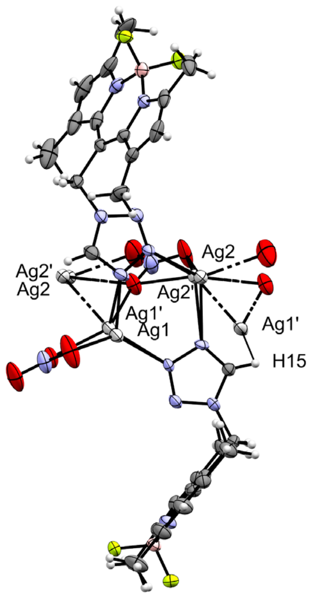

Figure S 17. Anagostic (H15-Ag1´) and intermetallic interactions (Ag1´-Ag2´) of coordination compound **11** with atomic number labeling of selected atoms (ellipsoids: 50 % probability level; atom color code: grey…C, blue…N, light green…F, pink…B, light grey…Ag, red…oxygen).

PXRD measurements

Powder X-ray diffraction experiments were performed on an “Empyrean” (Panalytical) diffractometer using Cu K-α radiation, a primary beam filter (Bragg-Brentano HD), a fixed ½° divergence slit, a 0.04 rad soller slit and a GaliPIX3D detector. Finely powdered bulk samples were positioned on a silicon single crystal cut along the (711) plane. It was measured from 3 to 45° 2 θ with a step size of 0.014°.

Diffractograms were evaluated using the PANalytical program suite HighScorePlus [4]; background correction was applied, and a calculated diffraction pattern derived from single-crystal CIF data was subsequently refined via Rietveld analysis against the measured PXRD data to confirm phase purity and structural consistency.

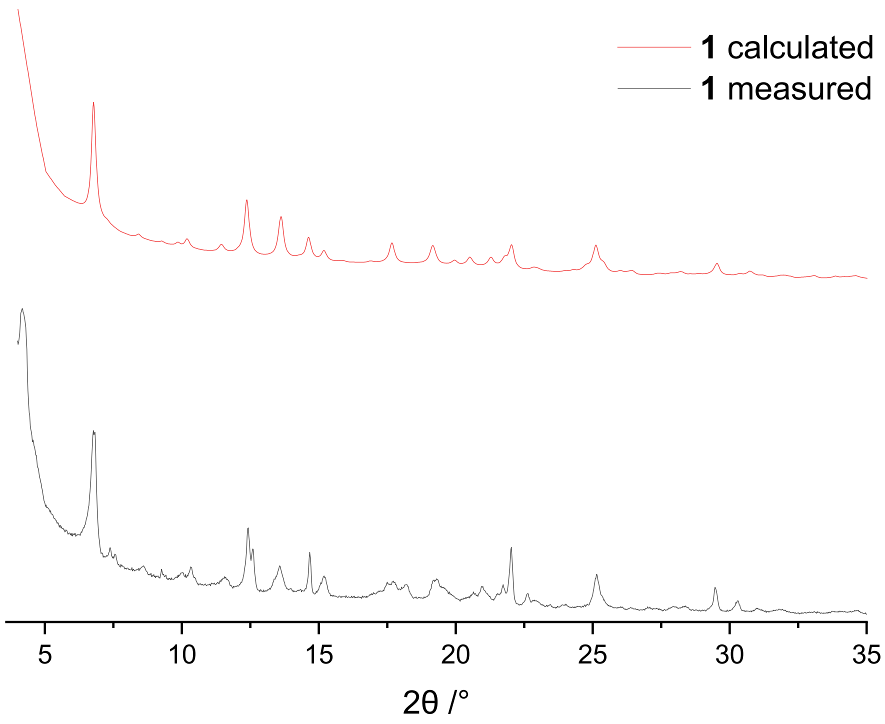
1 calculated
1 measured
5
10
15
20
25
30
35
2θ /°

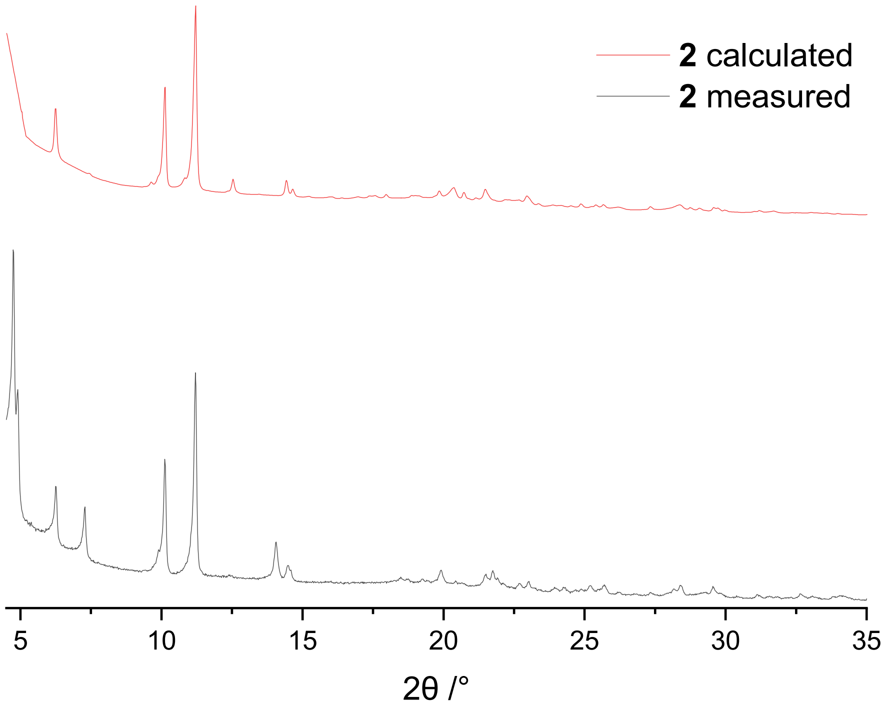
2 calculated
2 measured
5
10
15
20
25
30
35
2θ /°

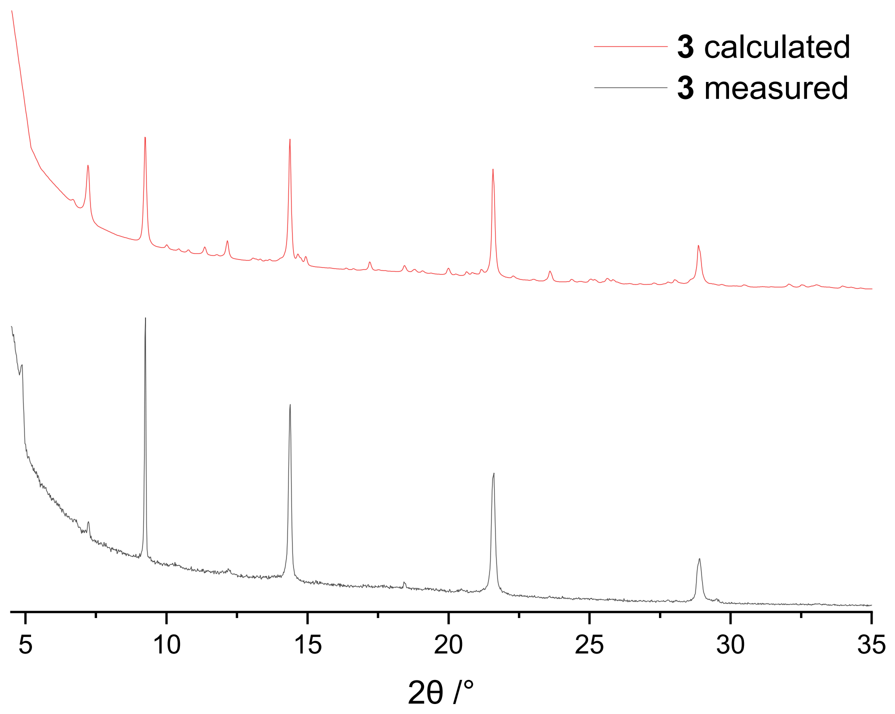

3 calculated
3 measured
5
10
15
20
25
30
35
2θ /°

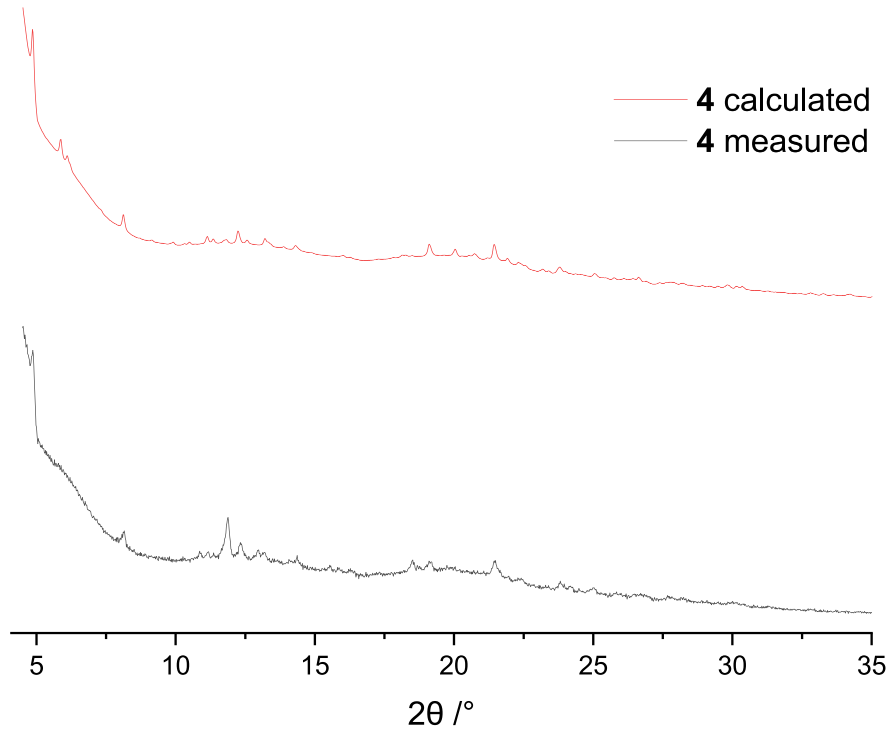

4 calculated
4 measured
5
10
15
20
25
30
35
2θ /°

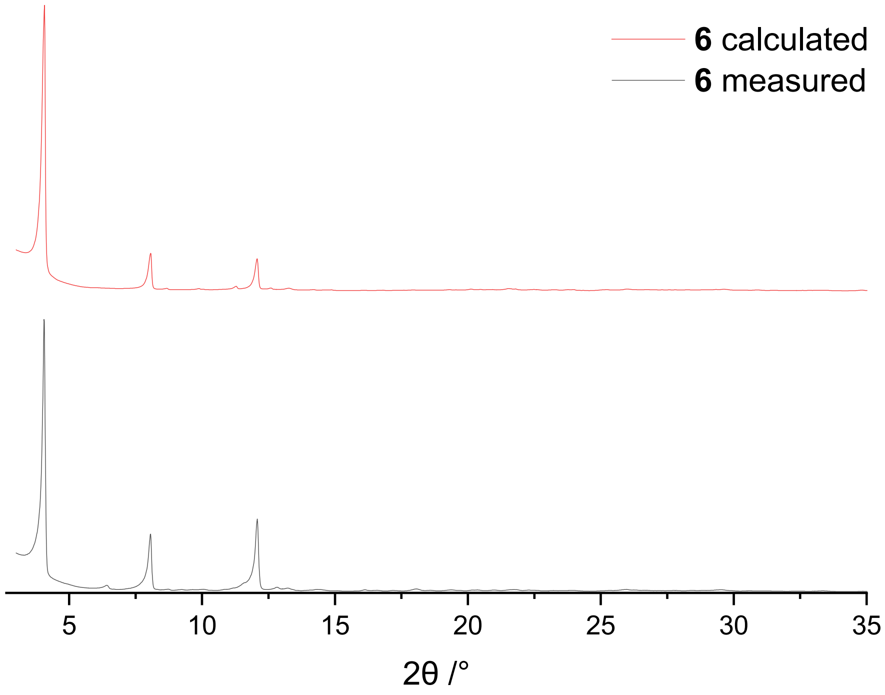
6 calculated
6 measured
5
10
15
20
25
30
35
2θ /°

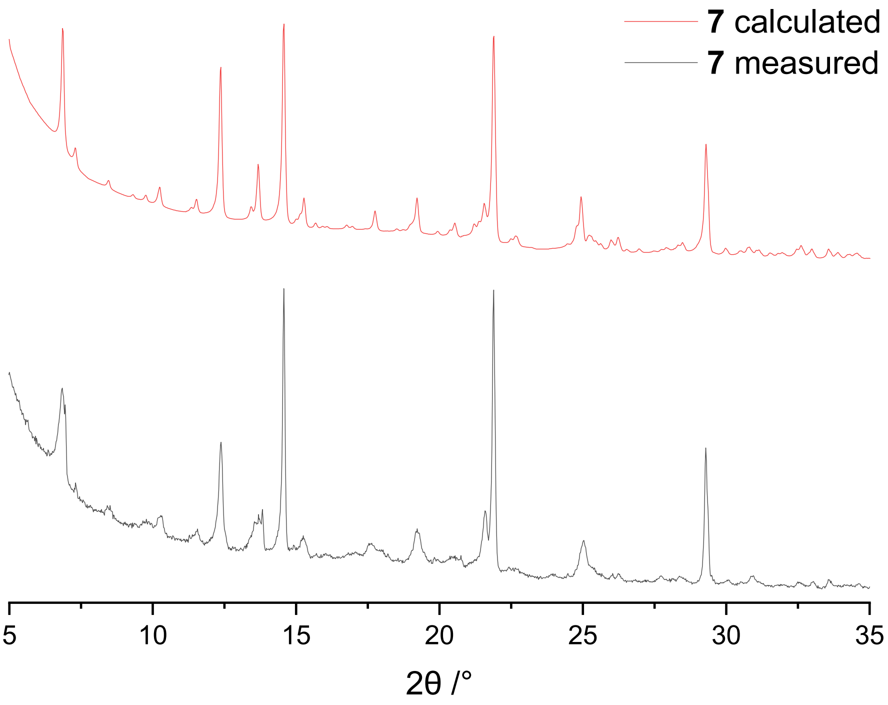
7 calculated
7 measured
5
10
15
20
25
30
35
2θ /°

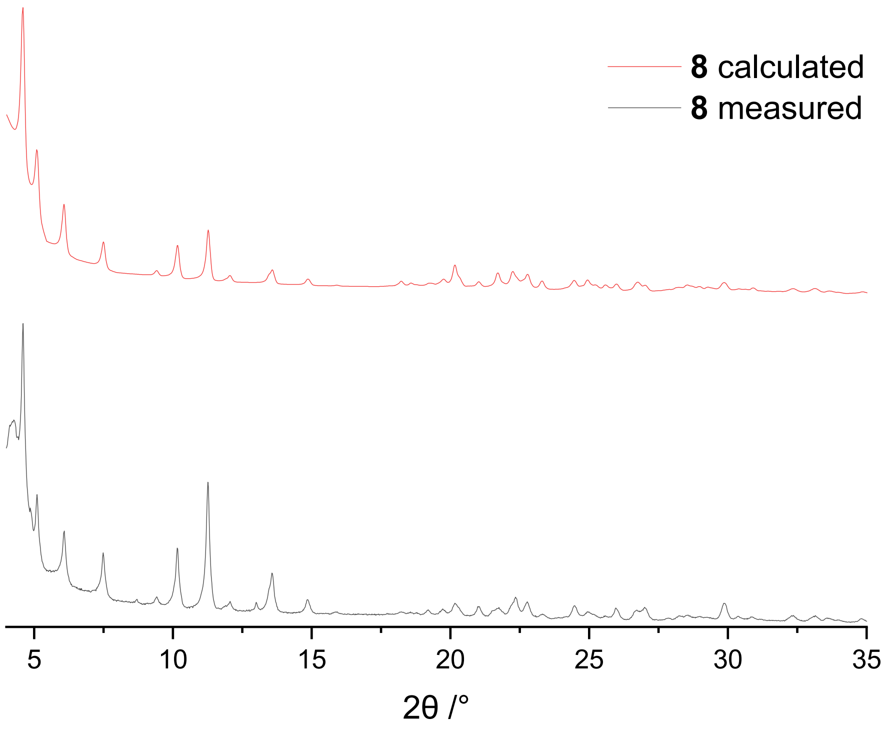

8 calculated
8 measured
5
10
15
20
25
30
35
2θ /°

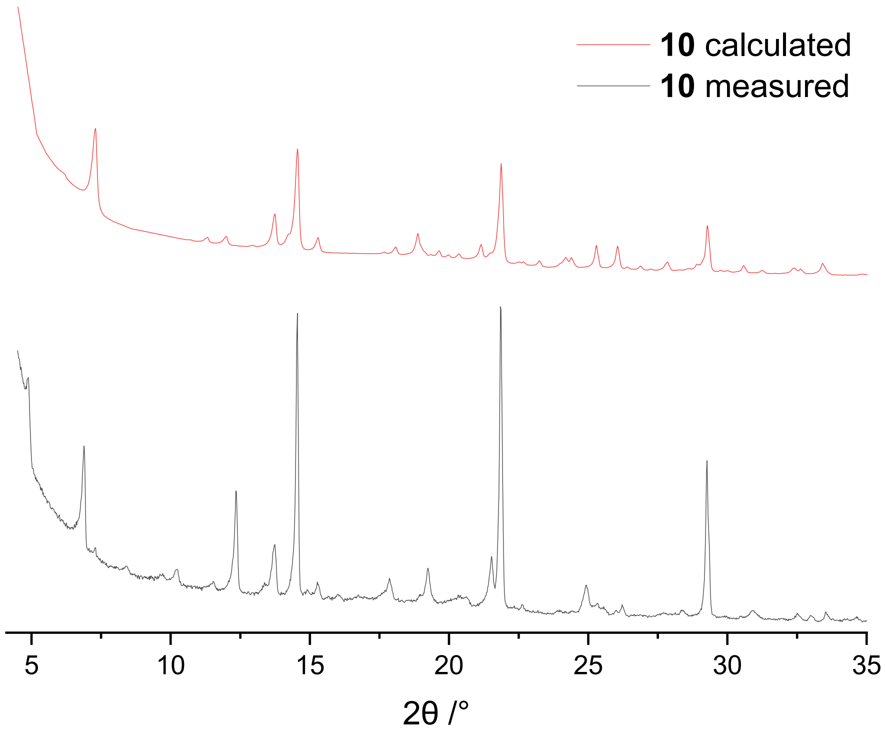

10 calculated
10 measured
5
10
15
20
25
30
35
2θ /°

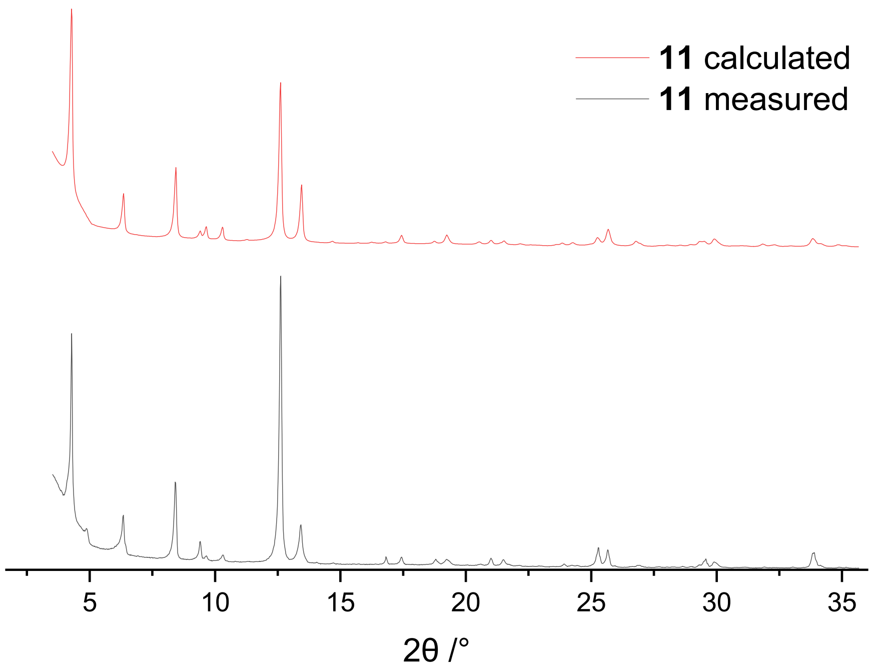

UV/Vis and PL measurements

All PL spectra were recorded with a FluoTime 300 fluorescence lifetime spectrometer (PicoQuant) equipped with a coaxial UV-Xenon arc lamp, a high-resolution excitation and emission double monochromator, and a PMA hybrid 07 detector. UV/Vis/NIR measurements were performed on a Lambda 900 spectrophotometer (PerkinElmer). Powder samples were measured in diffuse reflectance with a Harrick powder sample holder in “Praying Mantis” configuration against $BaSO_4$. Diffuse reflectance data (R) were converted using the Kubelka-Munk function, defined as $F(R) = \frac{(1-R)^2}{2R}$.

Diffuse reflectance and emission spectra

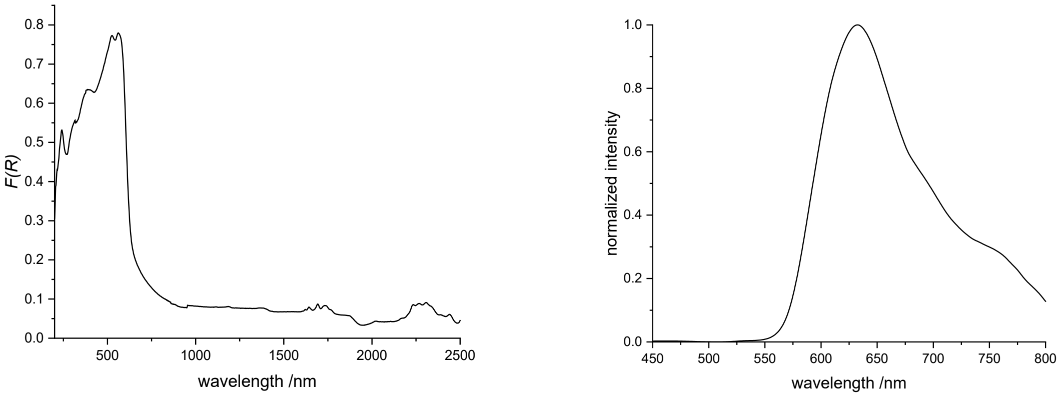

Figure S 18. Diffuse reflectance (left) and emission spectra (right) of **L** as powder sample. $\lambda_{exc.}$ = 424 nm.

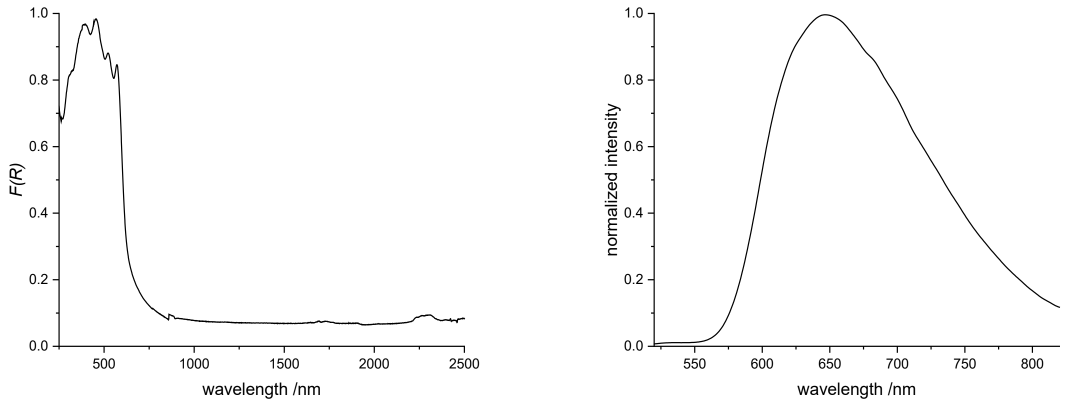

Figure S 19. Diffuse reflectance (left) and emission spectra (right) of **1** as powder sample. $\lambda_{exc.}$ = 424 nm.

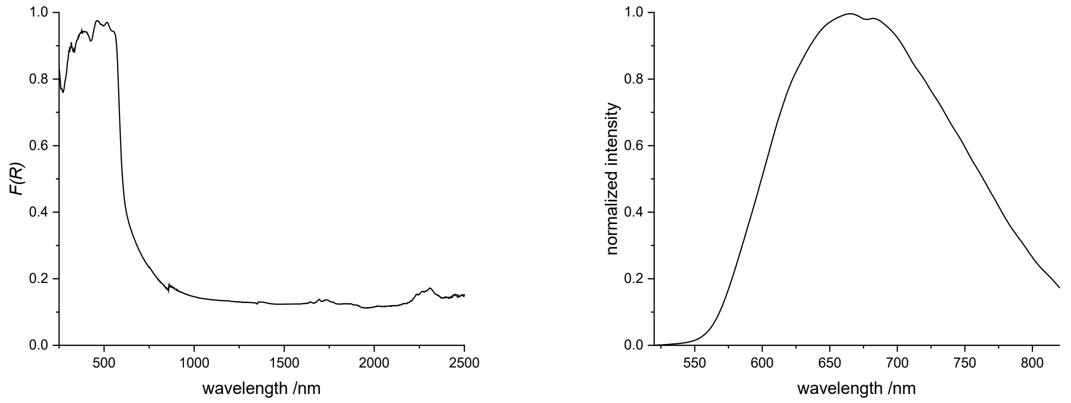

Figure S 20. Diffuse reflectance (left) and emission spectra (right) of **2** as powder sample. $\lambda_{exc.}$ = 424 nm.

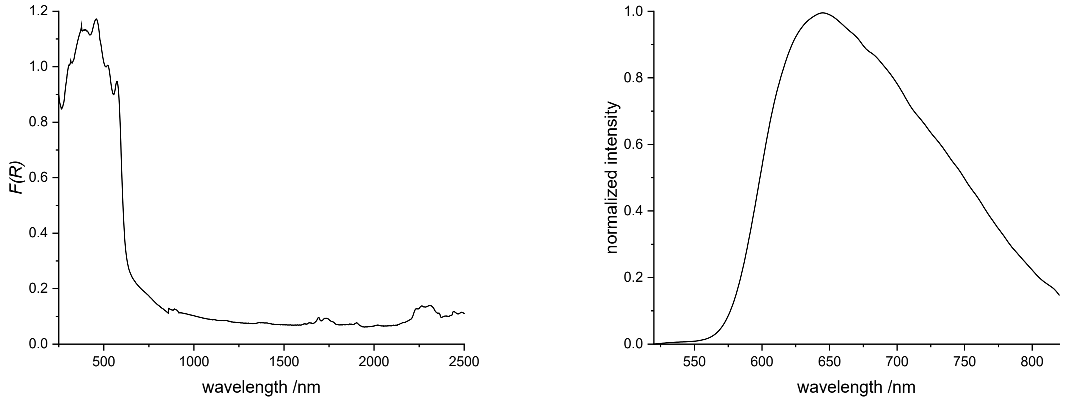

Figure S 21. Diffuse reflectance (left) and emission spectra (right) of **3** as powder sample. $\lambda_{exc.}$ = 424 nm.

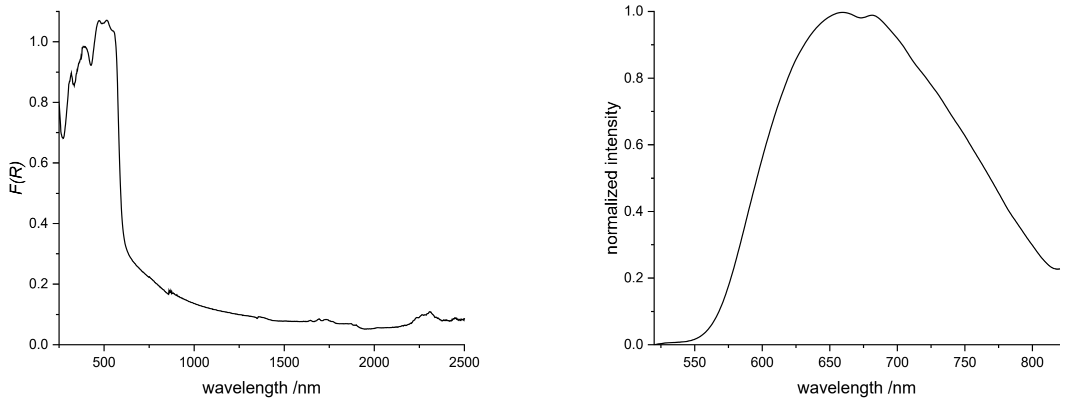

Figure S 22. Diffuse reflectance (left) and emission spectra (right) of **4** as powder sample. $\lambda_{exc.}$ = 424 nm.

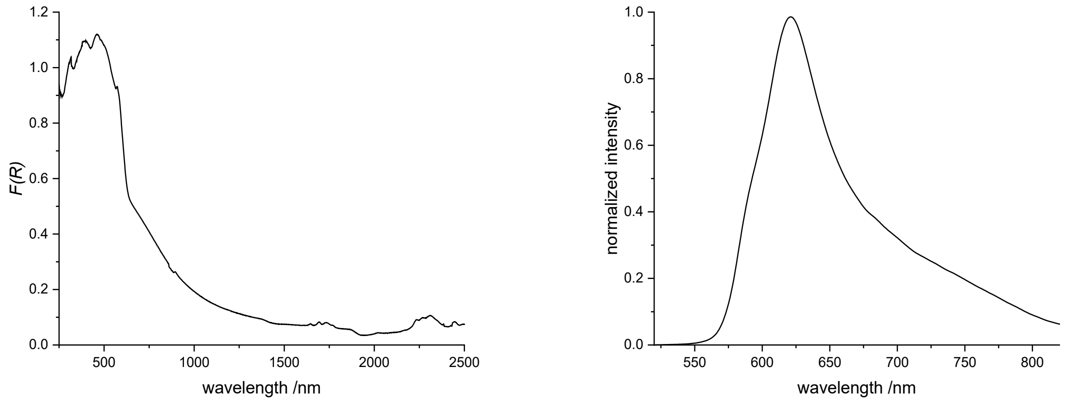

Figure S 23. Diffuse reflectance (left) and emission spectra (right) of **6** as powder sample. $\lambda_{exc.}$ = 424 nm.

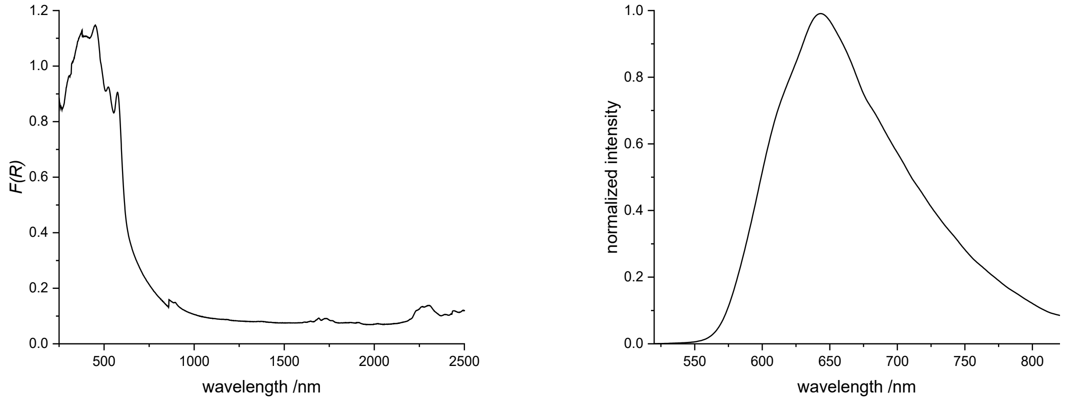

Figure S 24. Diffuse reflectance (left) and emission spectra (right) of **7** as powder sample. $\lambda_{exc.}$ = 424 nm.

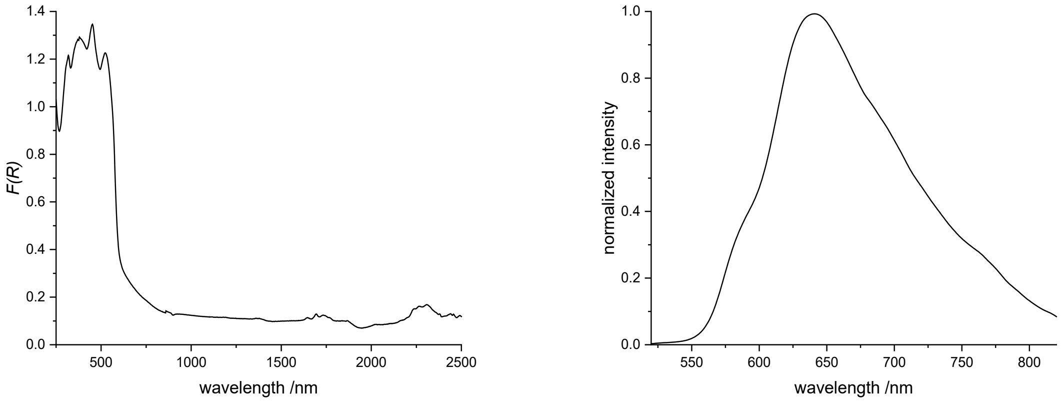

Figure S 25. Diffuse reflectance (left) and emission spectra (right) of **8** as powder sample. $\lambda_{exc.}$ = 424 nm.

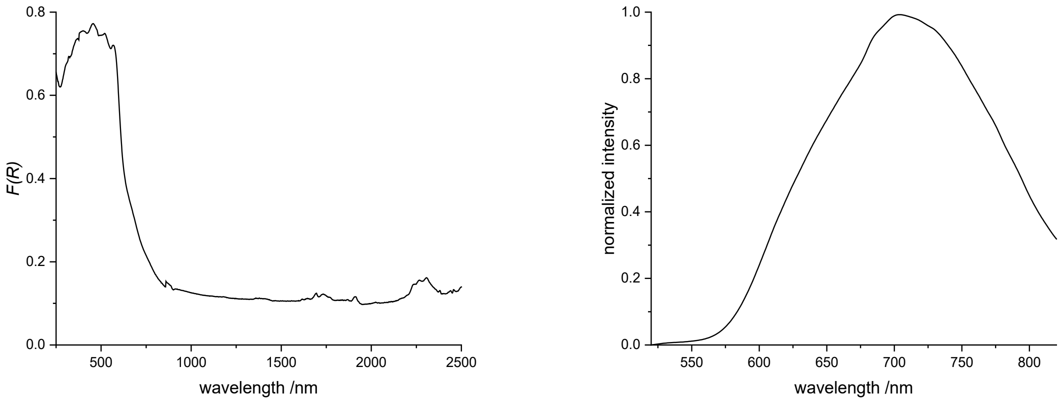

Figure S 26. Diffuse reflectance (left) and emission spectra (right) of **10** as powder sample. $\lambda_{exc.}$ = 424 nm.

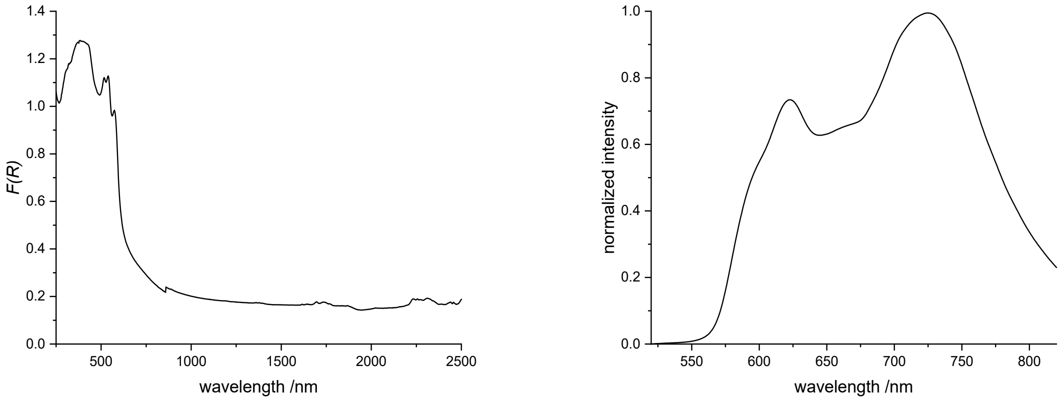

Figure S 27. Diffuse reflectance (left) and emission spectra (right) of **11** as powder sample. $\lambda_{exc.}$ = 424 nm.

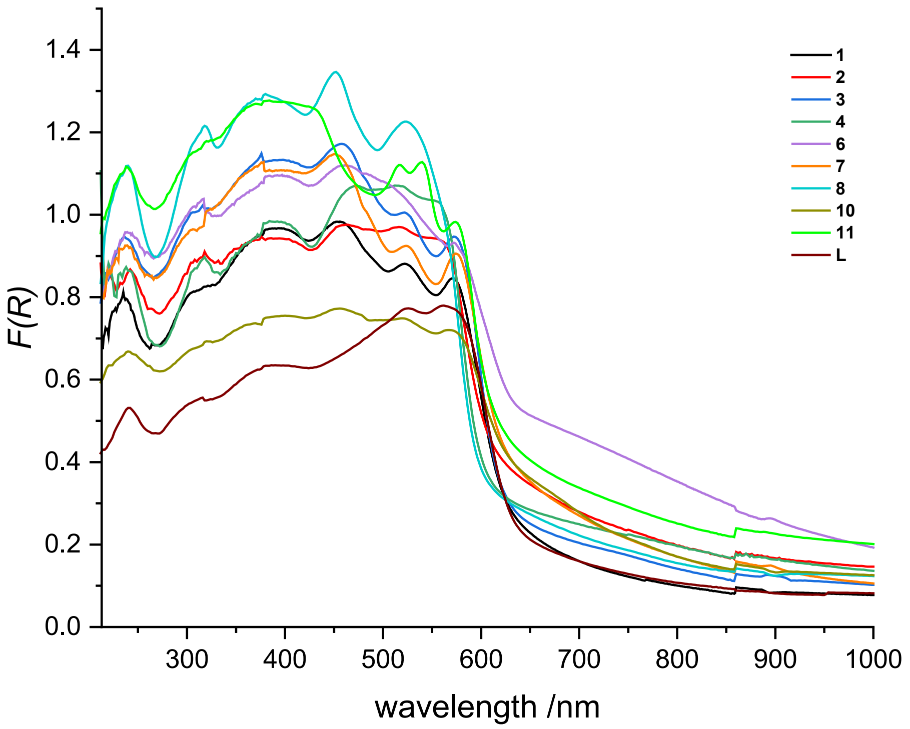

Figure S 28. Diffuse reflectance spectra of **1** (black), **2** (red), **3** (blue), **4** (dark green), **6** (purple), **7** (orange), **8** (turquoise), **10** (olive), **11** (light green), **L** (brown) as powder sample.

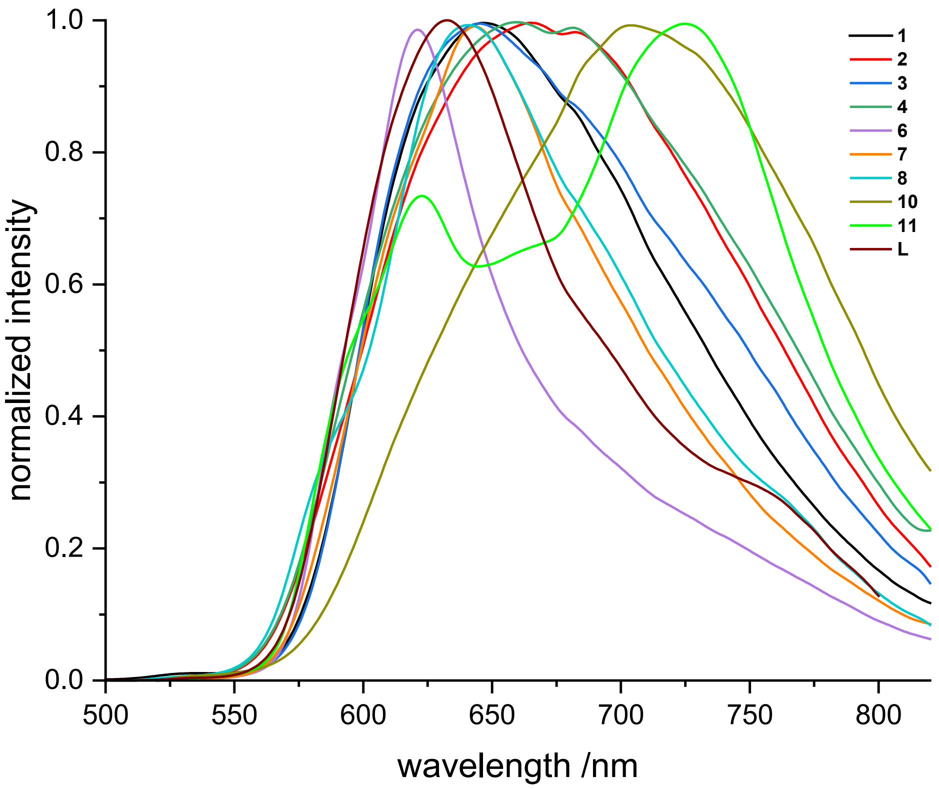

Figure S 29. Normalized emission spectra of **1** (black), **2** (red), **3** (blue), **4** (dark green), **6** (purple), **7** (orange), **8** (turquoise), **10** (olive), **11** (light green), **L** (brown) as powder sample. $\lambda_{exc.}$ = 424 nm.

NMR measurements

NMR measurements were performed in dry nitrobenzene-d5 on a Bruker Avance UltraShield 400 MHz or a 600 MHz spectrometer. The recorded ^{1}H and $^{13}C\{^{1}H\}$ spectra were internally referenced to residual proton signals and solvent resonances, respectively, and reported relative to tetramethylsilane (δ = 0 ppm). $^{11}B\{^{1}H\}$, ^{19}F, $^{31}P\{^{1}H\}$ spectra were referenced externally (δ = 0 ppm) to $BF_3 \cdot Et_2O$ (15 % in $CDCl_3$), $CFCl_3$ and H_3PO_4 (85 %) respectively.

NMR spectra

^{1}H-NMR ($C_6D_5NO_2$, 600 MHz)

1

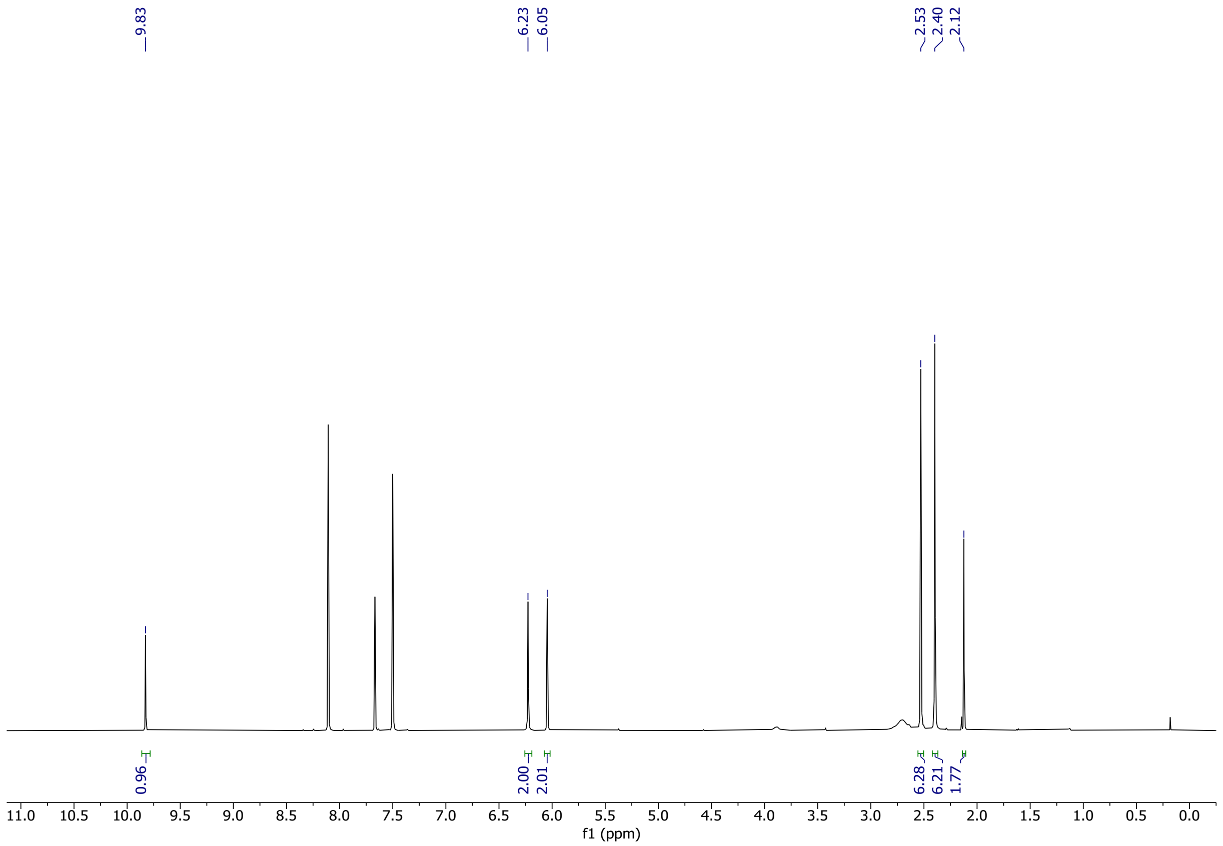

$^{13}C\{^{1}H\}$-NMR ($C_6D_5NO_2$, 151 MHz)

1

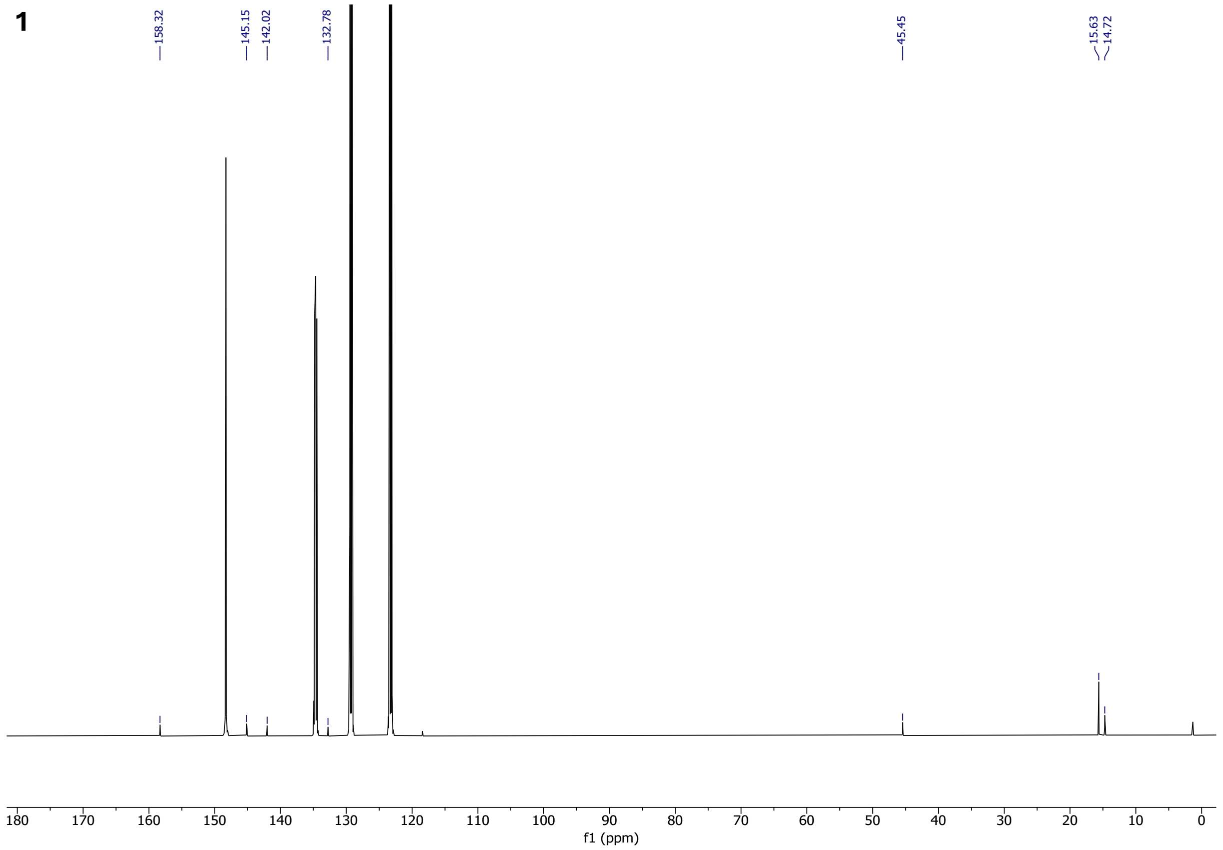

^{19}F-NMR ($C_6D_5NO_2$, 565 MHz)

1

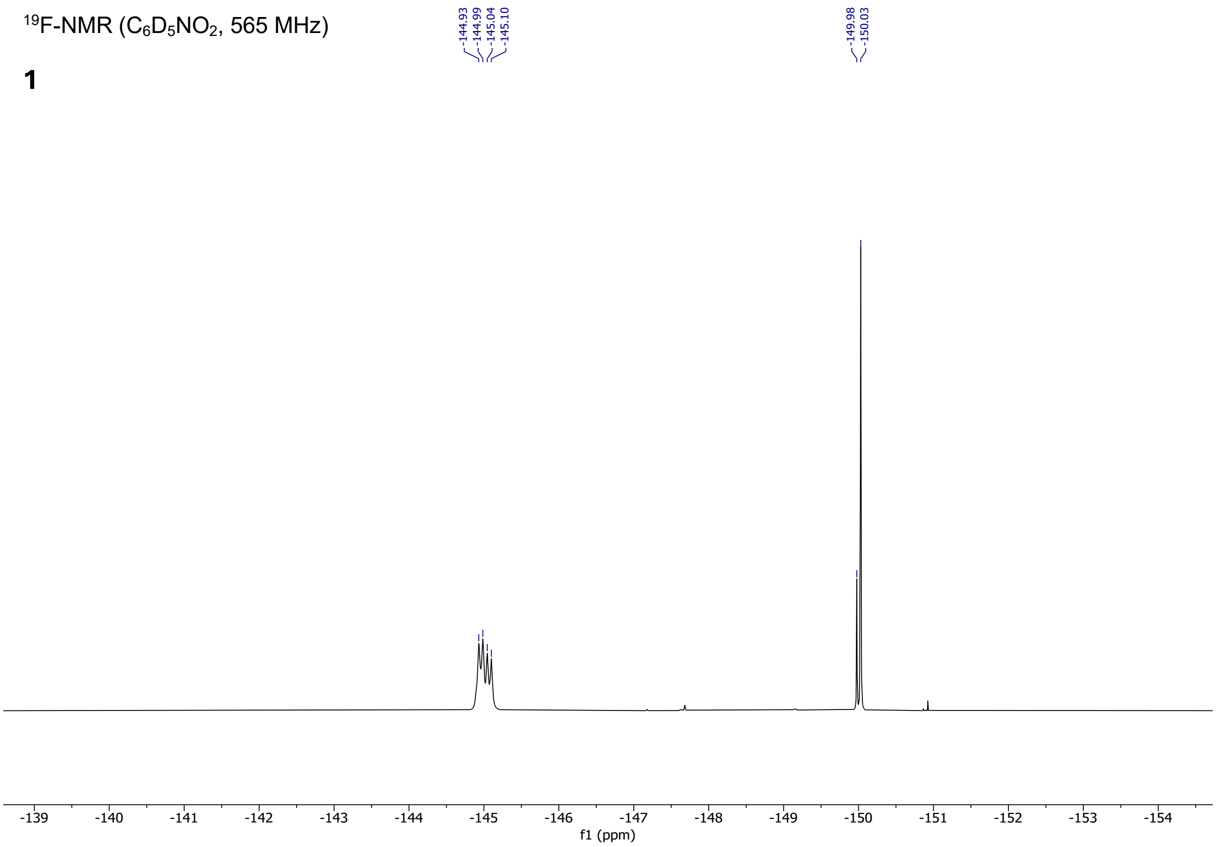

$^{11}B\{^1H\}$-NMR ($C_6D_5NO_2$, 193 MHz)

1

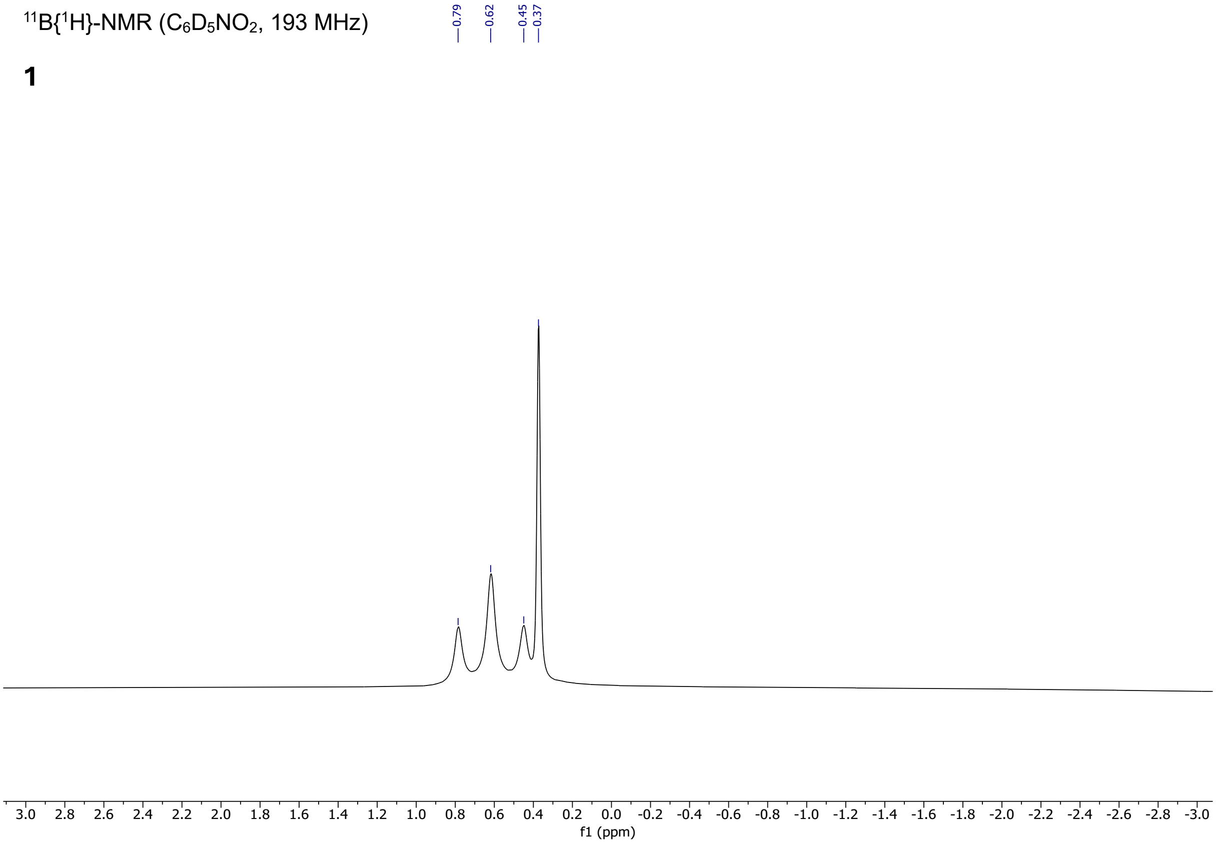

^{1}H-NMR ($C_6D_5NO_2$, 600 MHz)

2

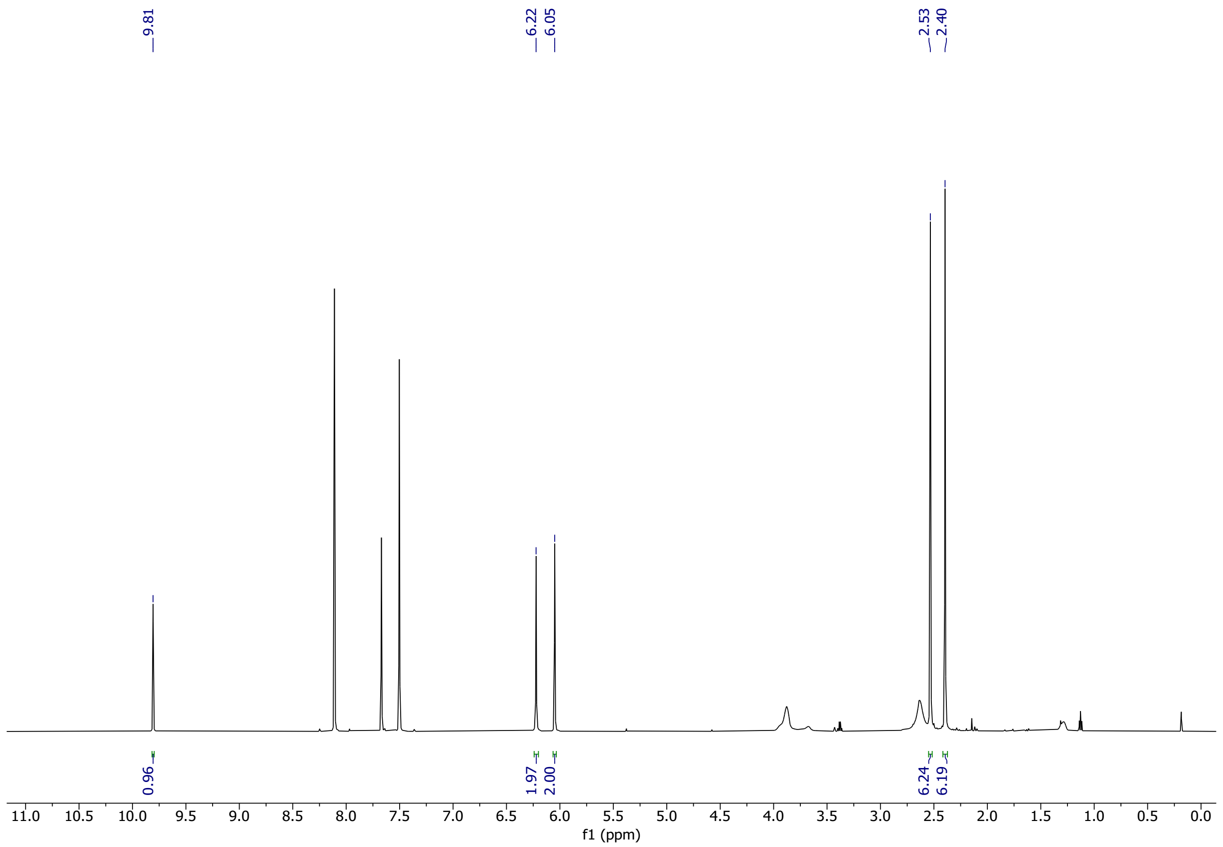

^{13}C{^{1}H}-NMR ($C_6D_5NO_2$, 151 MHz)

2

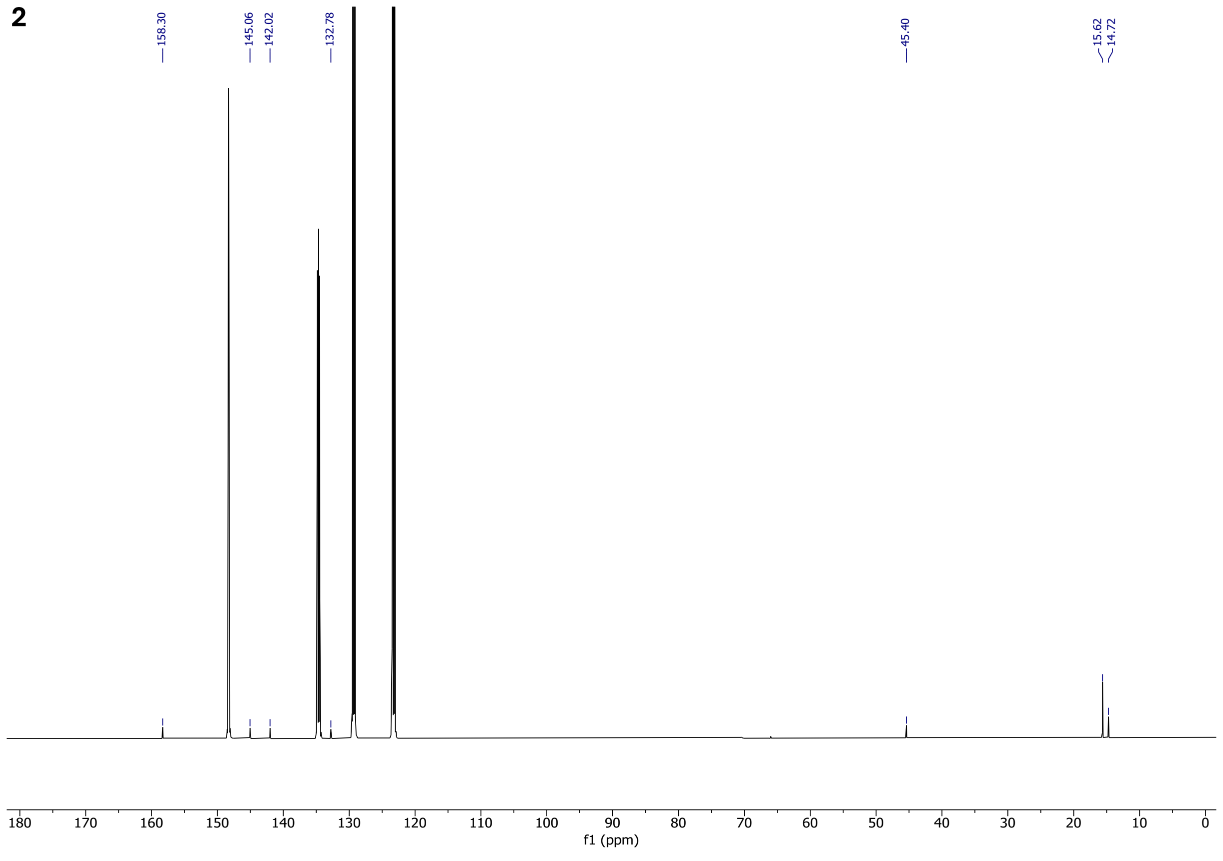

^{19}F-NMR ($C_6D_5NO_2$, 565 MHz)

2

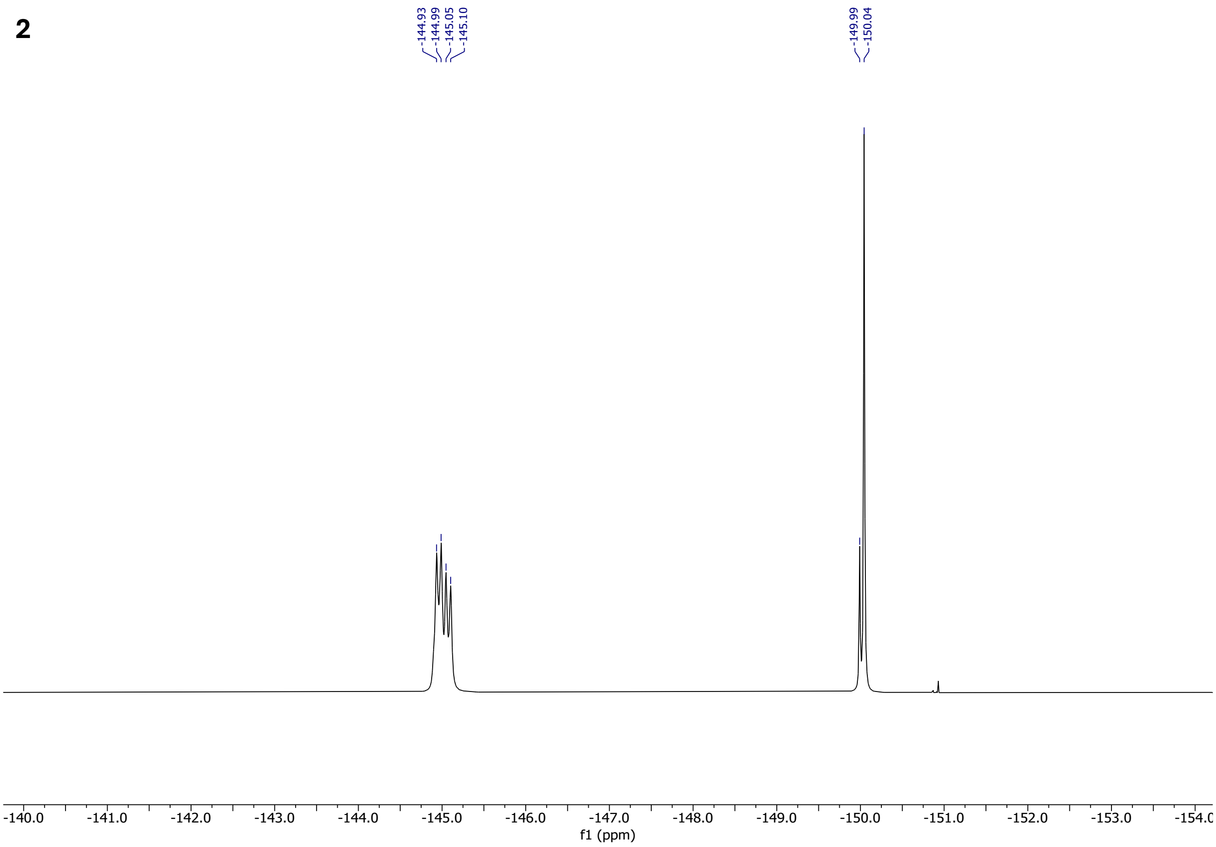

$^{11}B\{^1H\}$-NMR ($C_6D_5NO_2$, 193 MHz)

2

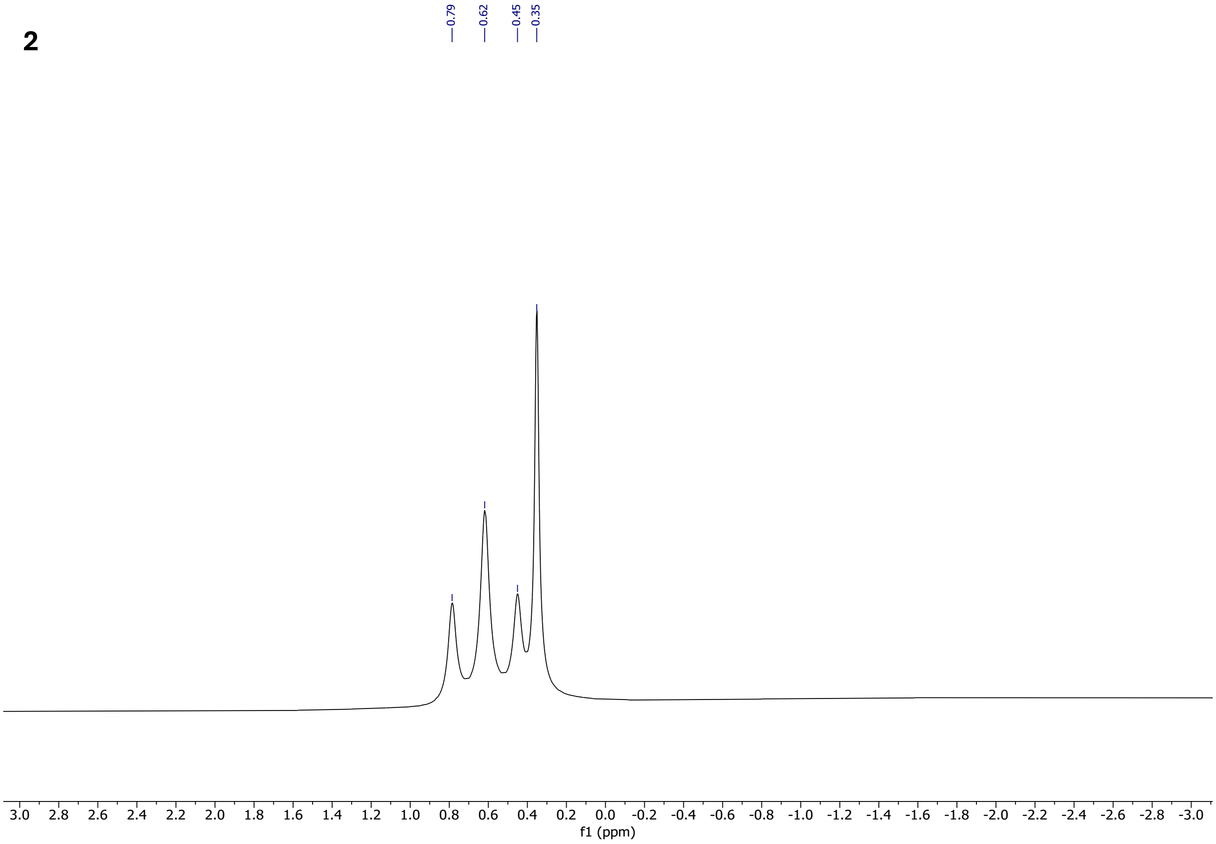

^{1}H-NMR ($C_6D_5NO_2$, 600 MHz)

3

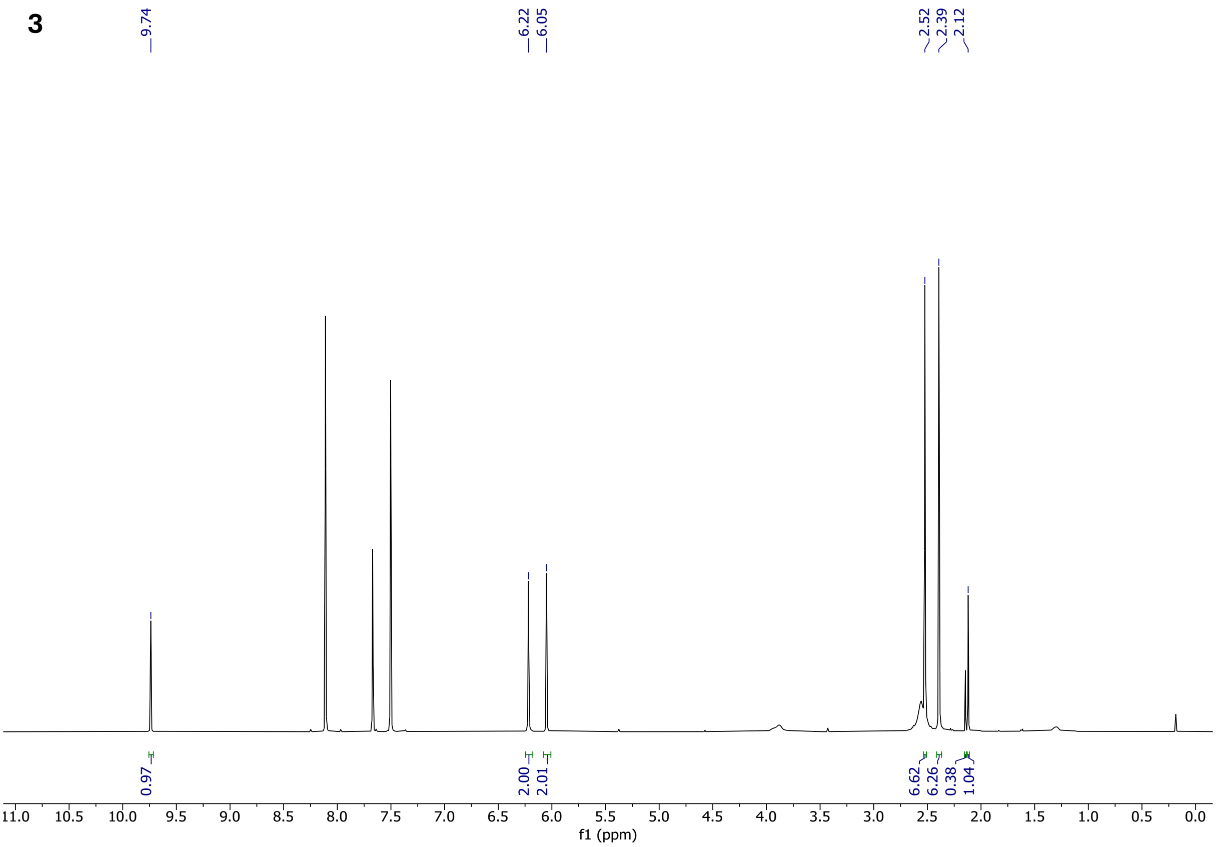

$^{13}C\{^{1}H\}$-NMR ($C_6D_5NO_2$, 151 MHz)

3

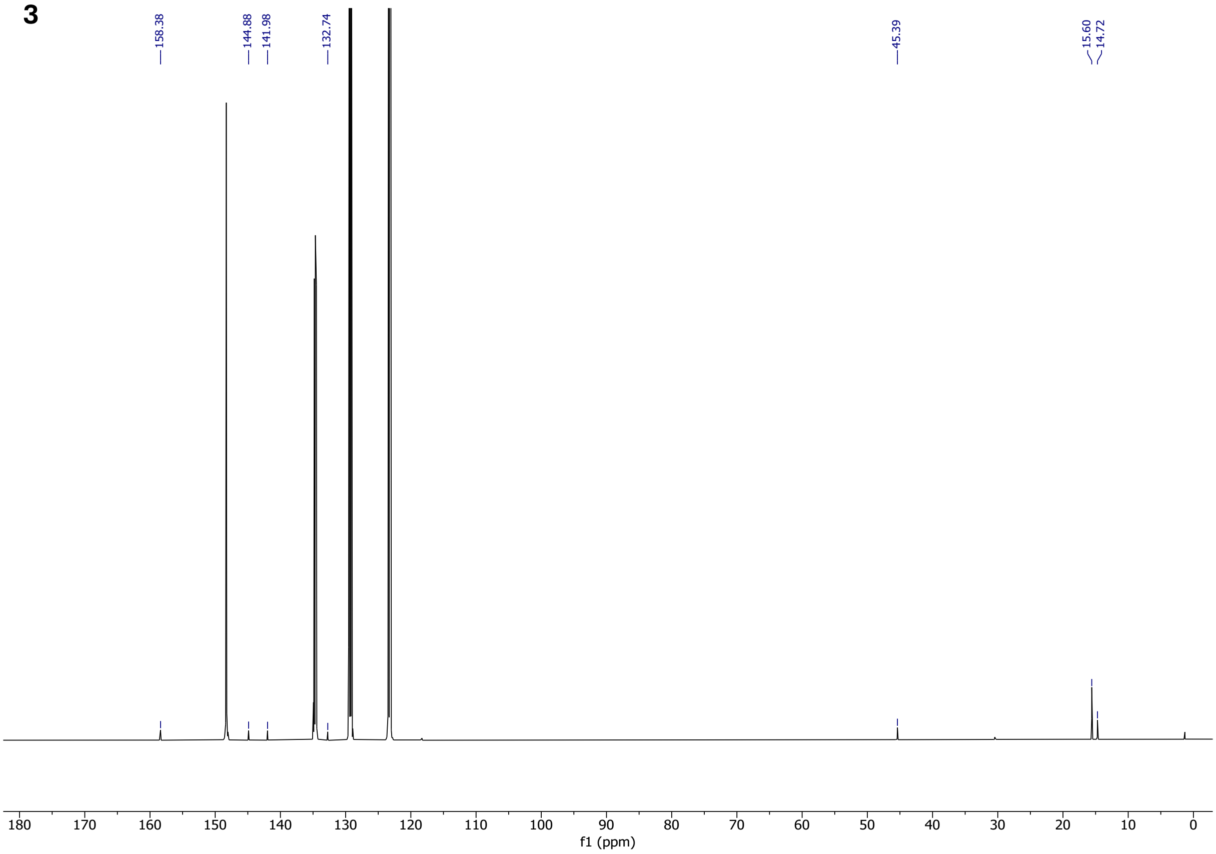

^{19}F-NMR ($C_6D_5NO_2$, 565 MHz)

3

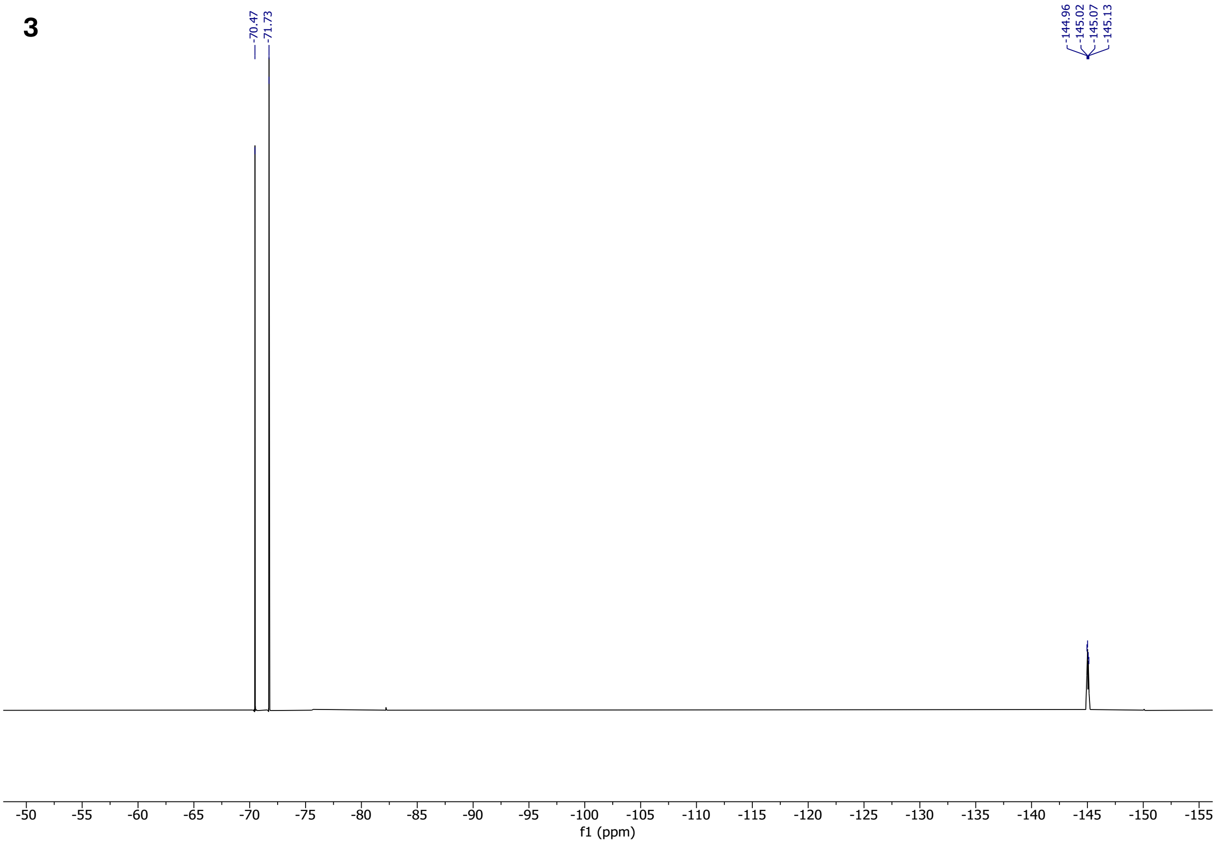

^{11}B{^{1}H}-NMR ($C_6D_5NO_2$, 193 MHz)

3

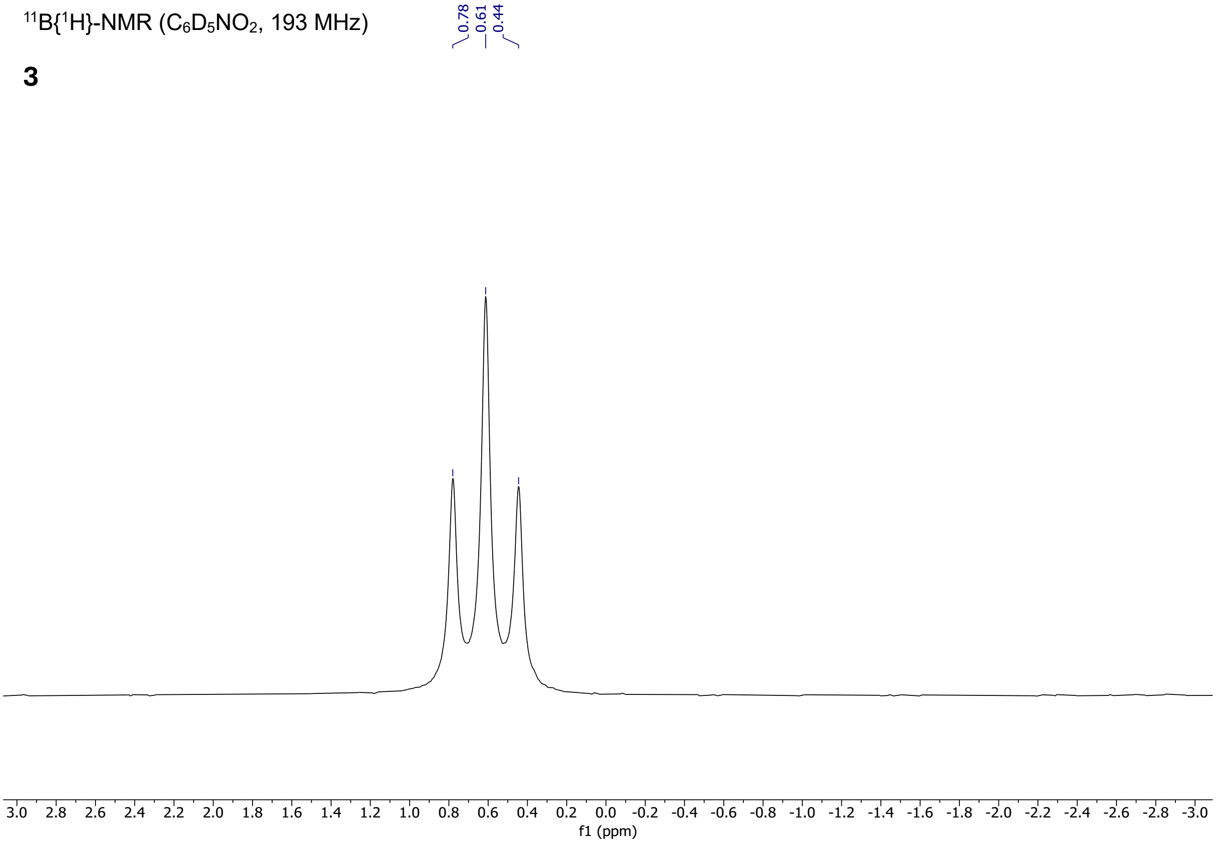

$^{31}P\{^{1}H\}$-NMR ($C_6D_5NO_2$, 243 MHz)

3

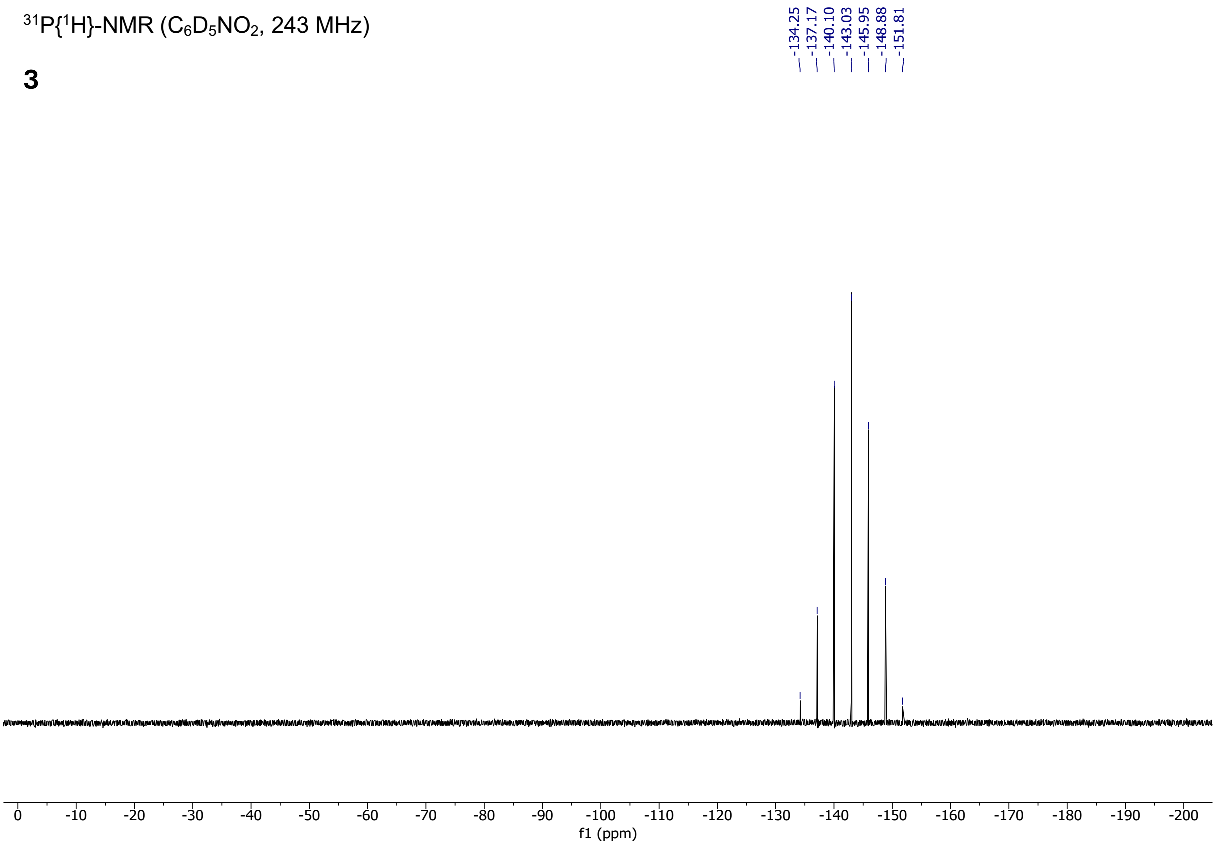

^{1}H-NMR ($C_6D_5NO_2$, 600 MHz)

4

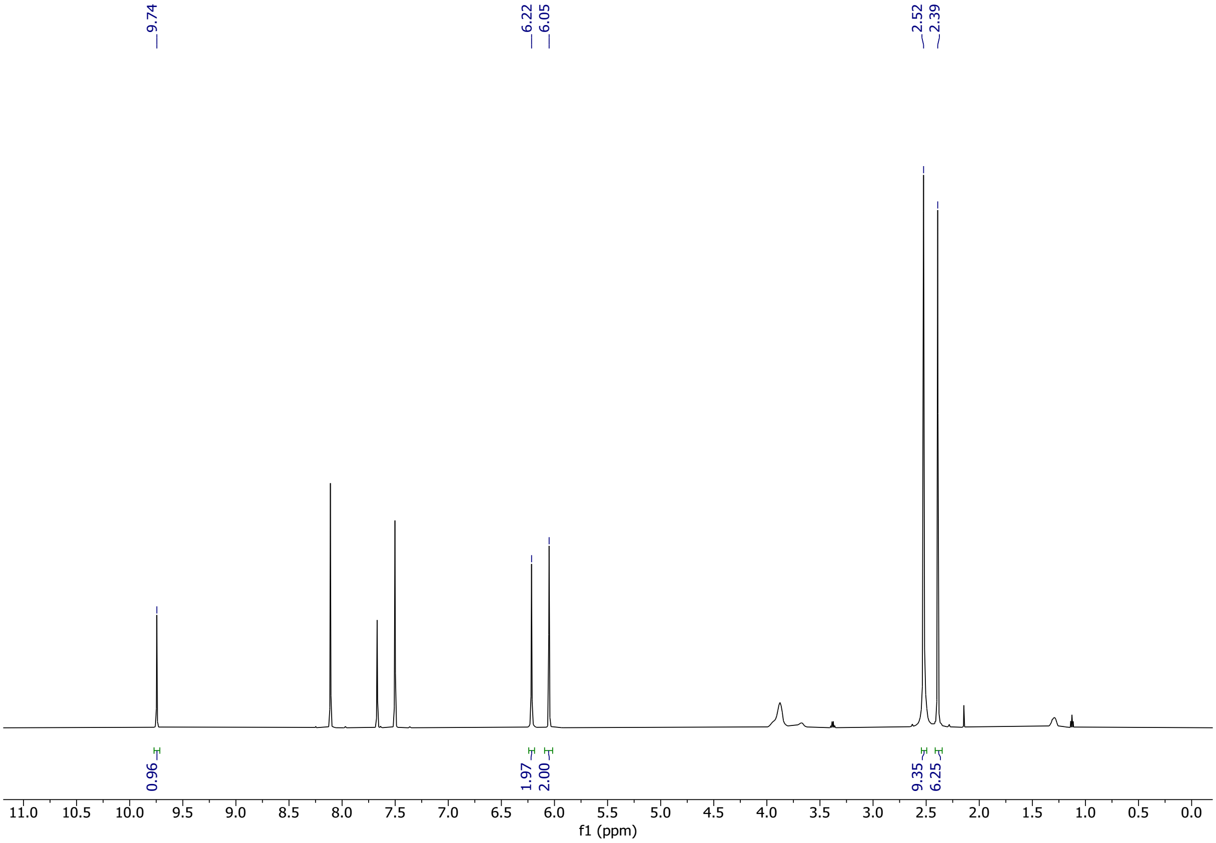

$^{13}C\{^{1}H\}$-NMR ($C_6D_5NO_2$, 151 MHz)

4

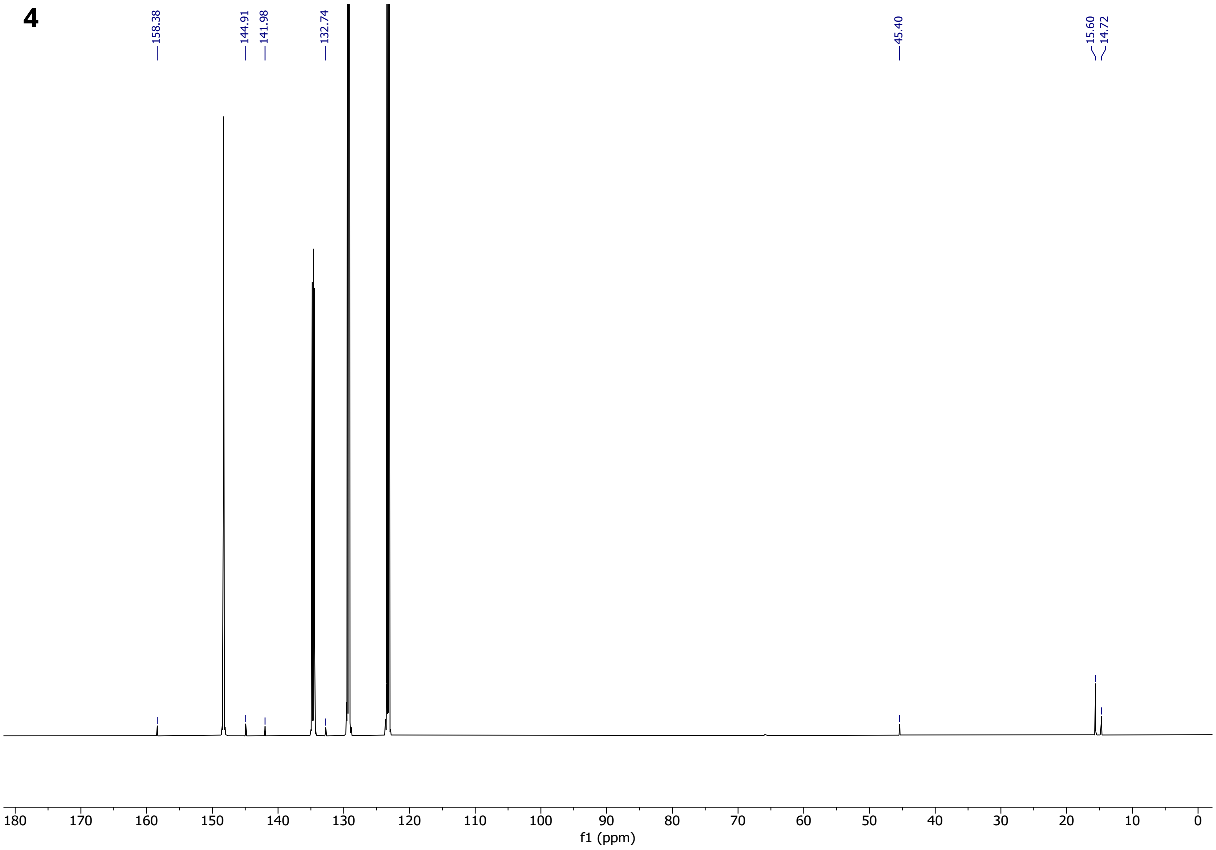

^{19}F-NMR ($C_6D_5NO_2$, 565 MHz)

4

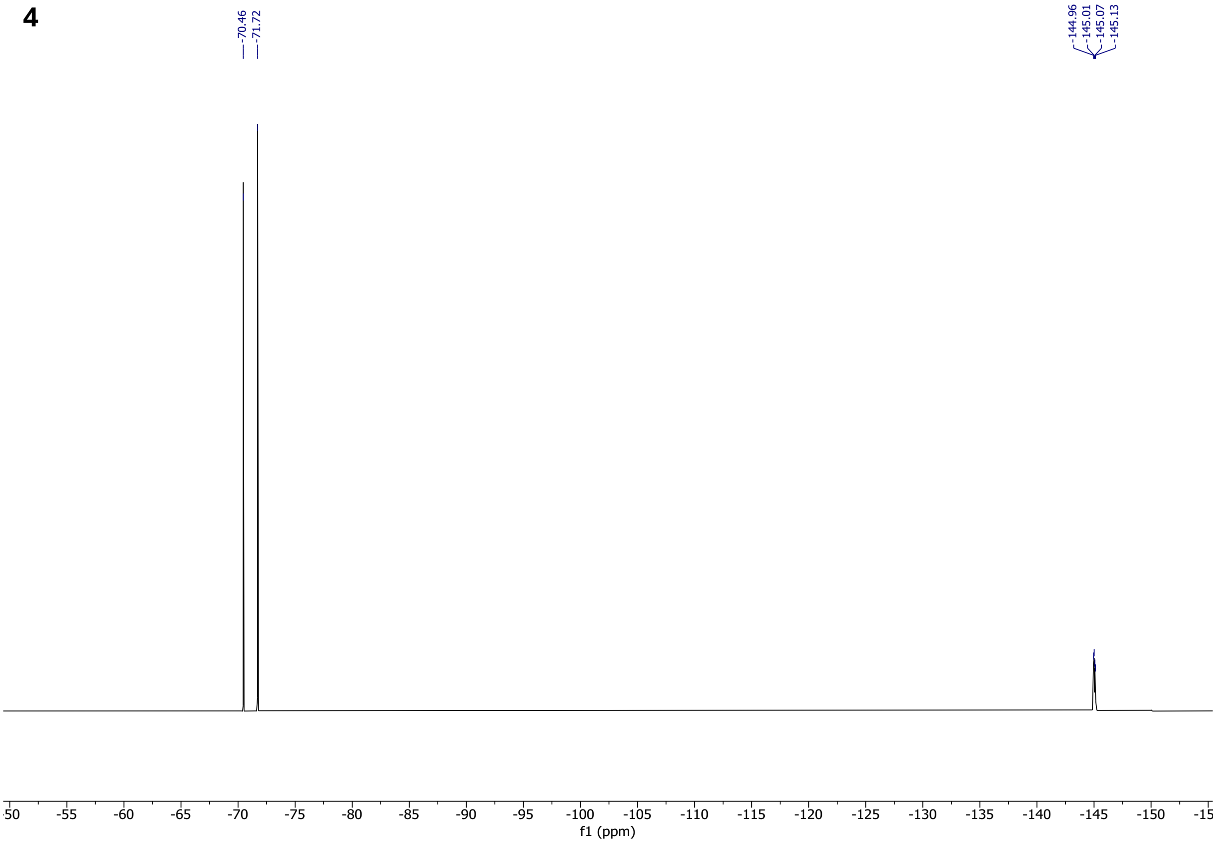

$^{11}B\{^{1}H\}$-NMR ($C_6D_5NO_2$, 193 MHz)

4

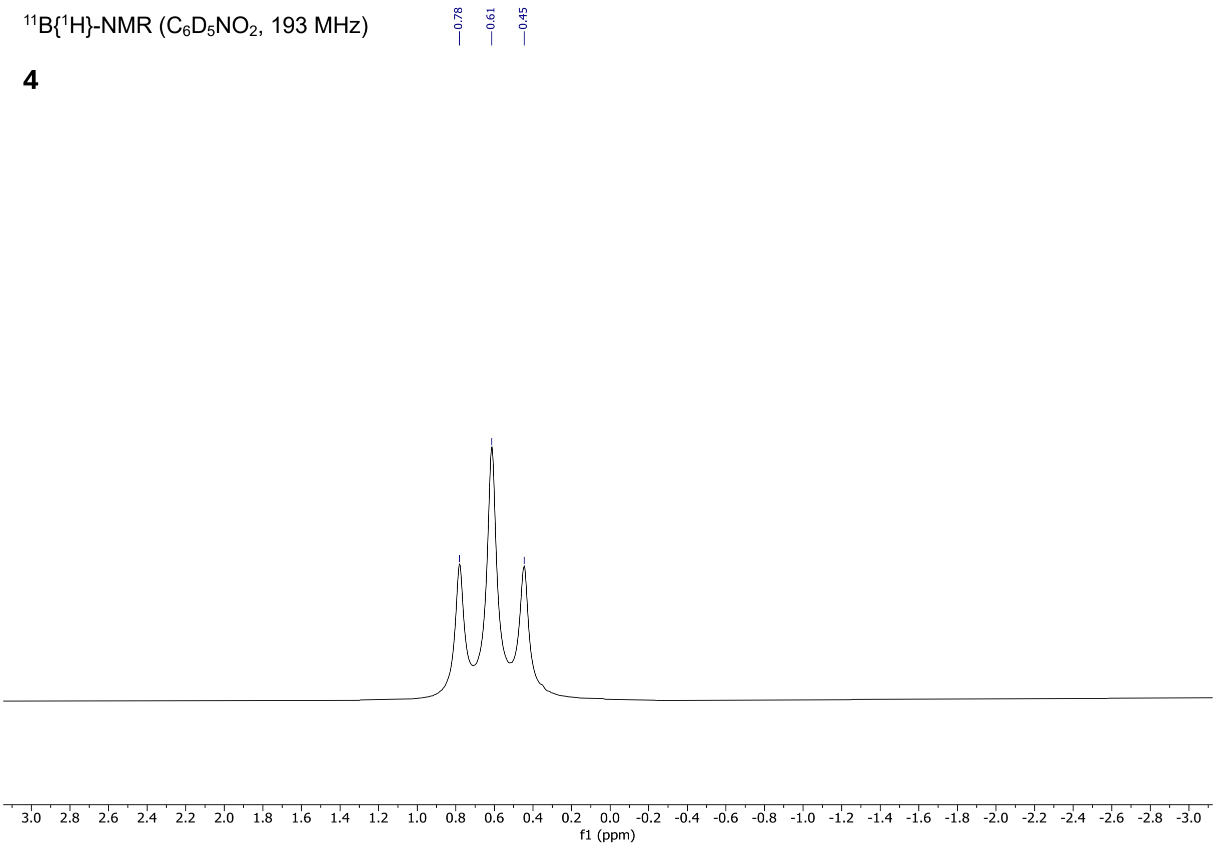

$^{31}P\{^{1}H\}$-NMR ($C_6D_5NO_2$, 243 MHz)

4

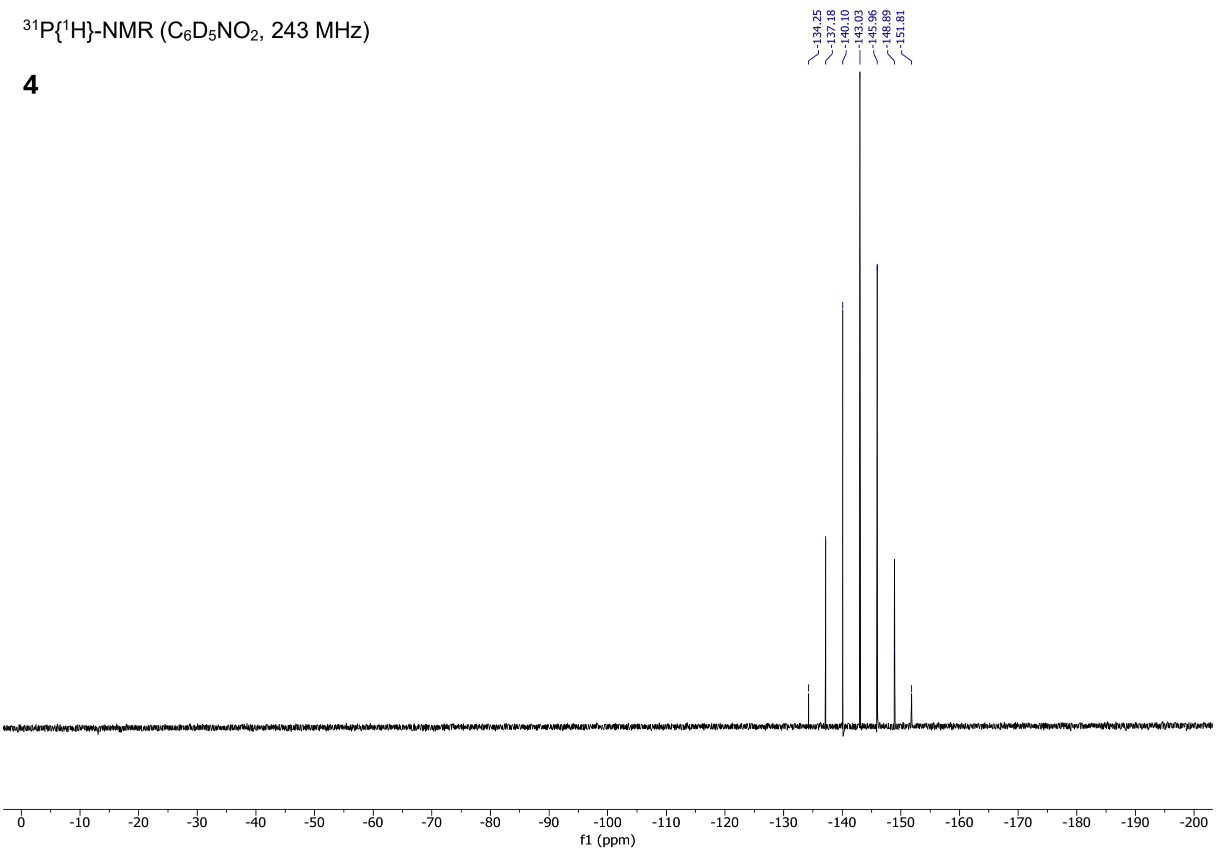

^{1}H-NMR ($C_6D_5NO_2$, 600 MHz)

6

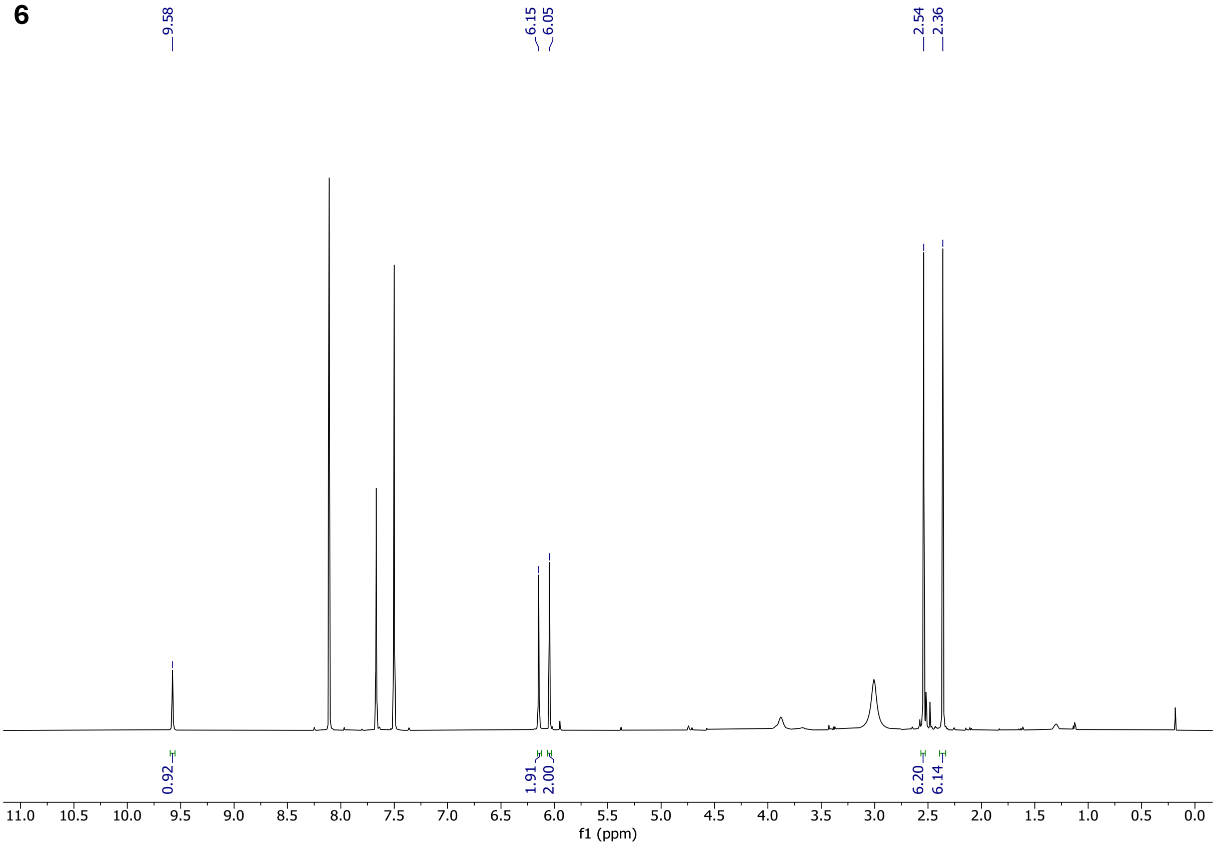

$^{13}C\{^{1}H\}$-NMR ($C_6D_5NO_2$, 151 MHz)

6

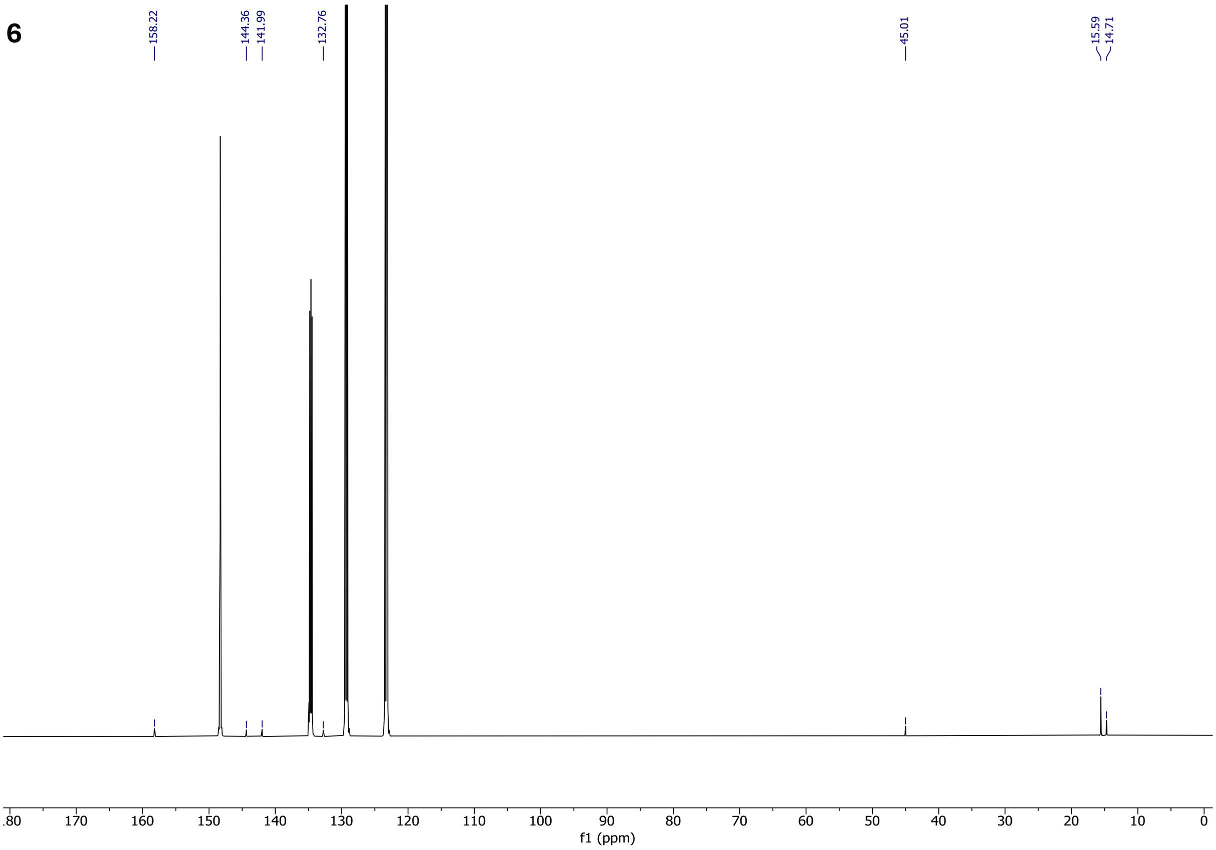

^{19}F-NMR ($C_6D_5NO_2$, 565 MHz)

6

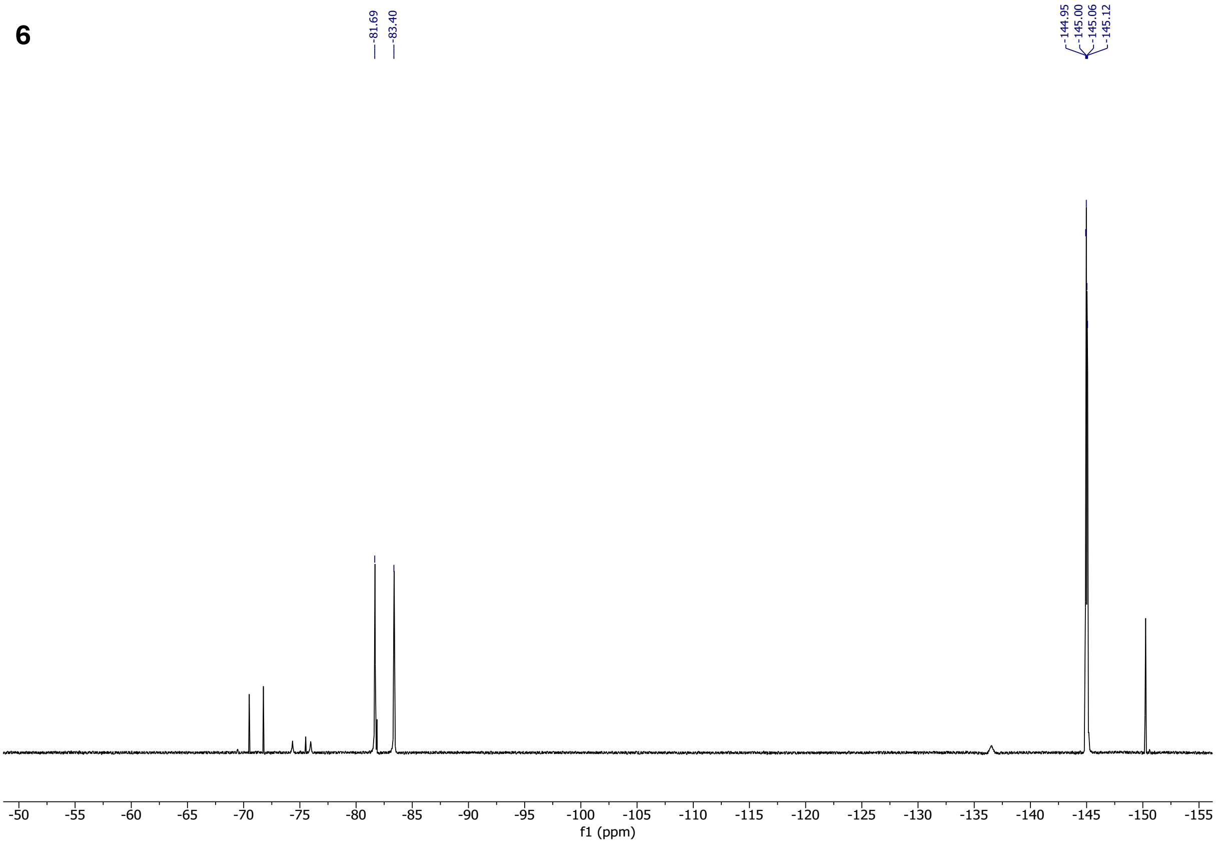

$^{11}B\{^1H\}$-NMR ($C_6D_5NO_2$, 193 MHz)

6

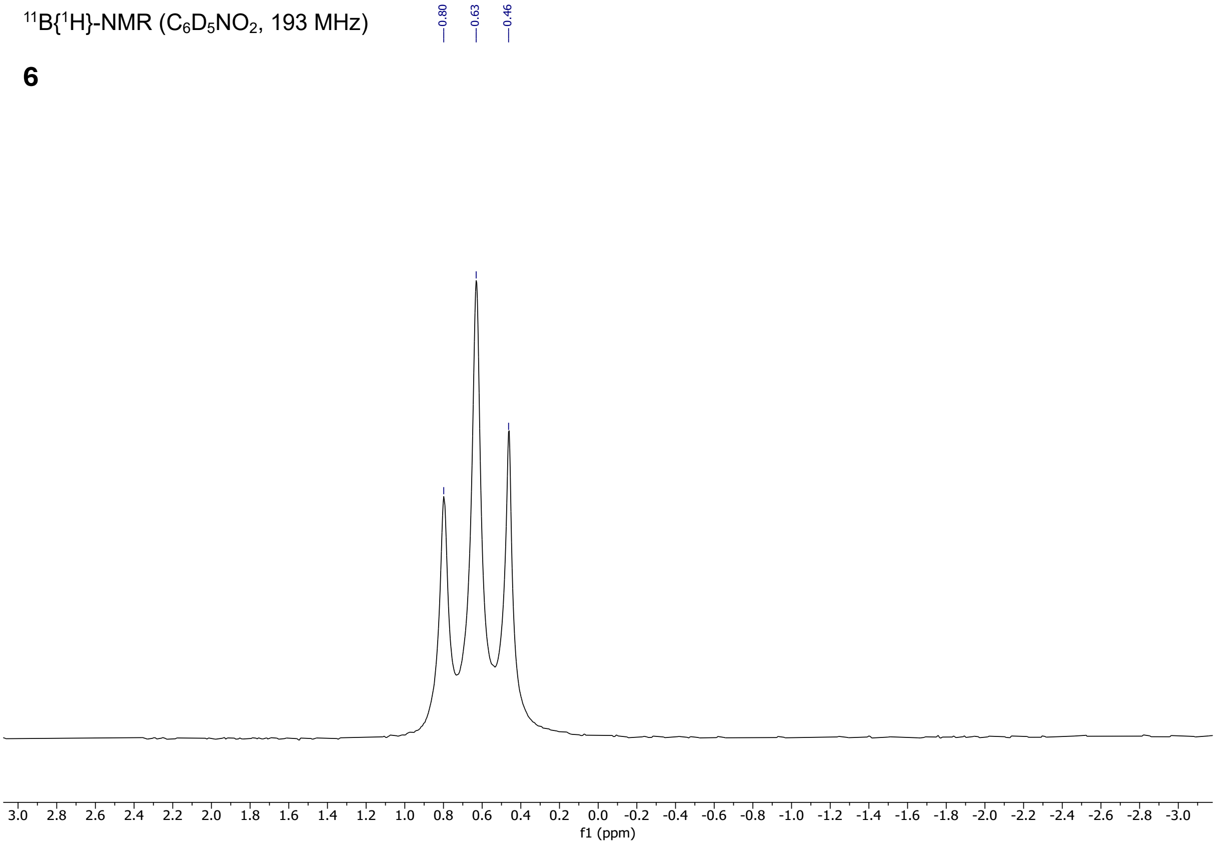

$^{31}P\{^1H\}$-NMR ($C_6D_5NO_2$, 243 MHz)

6

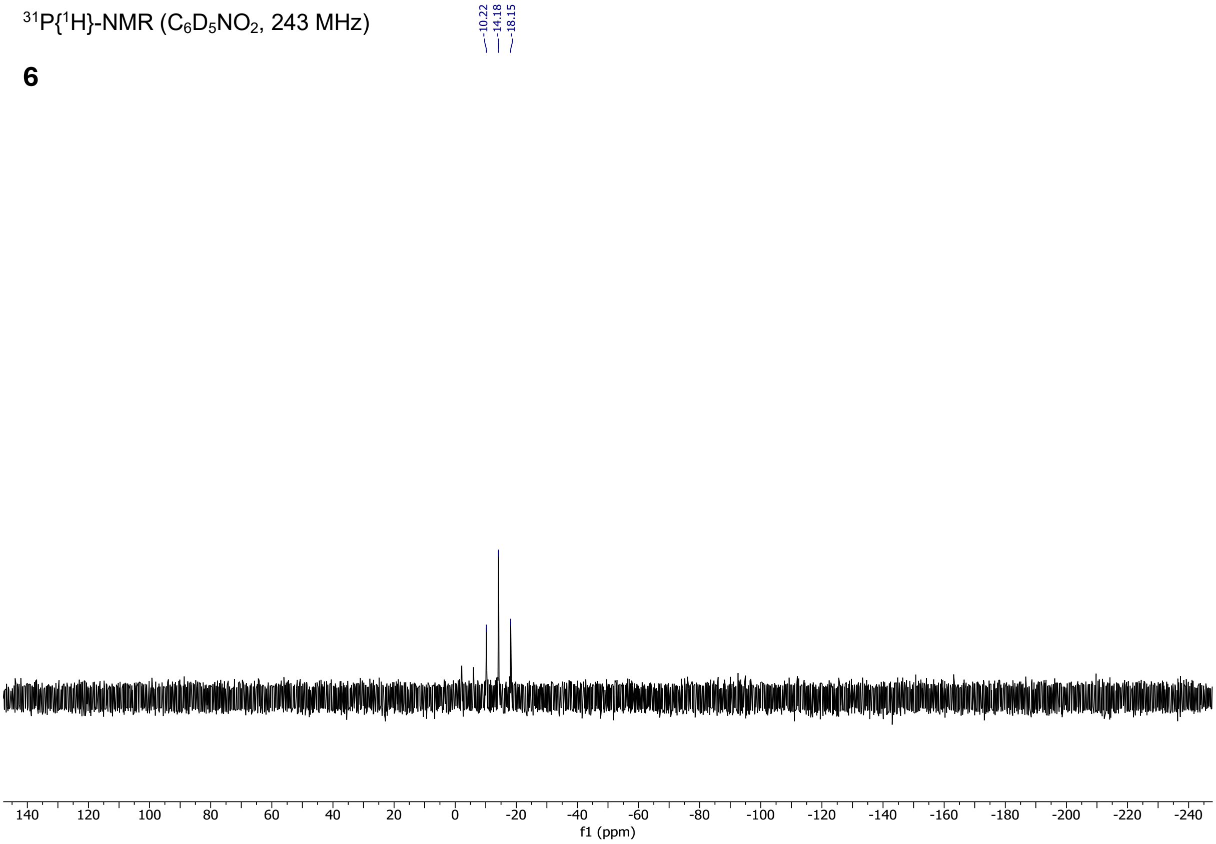

^1H-NMR ($C_6D_5NO_2$, 600 MHz)

7

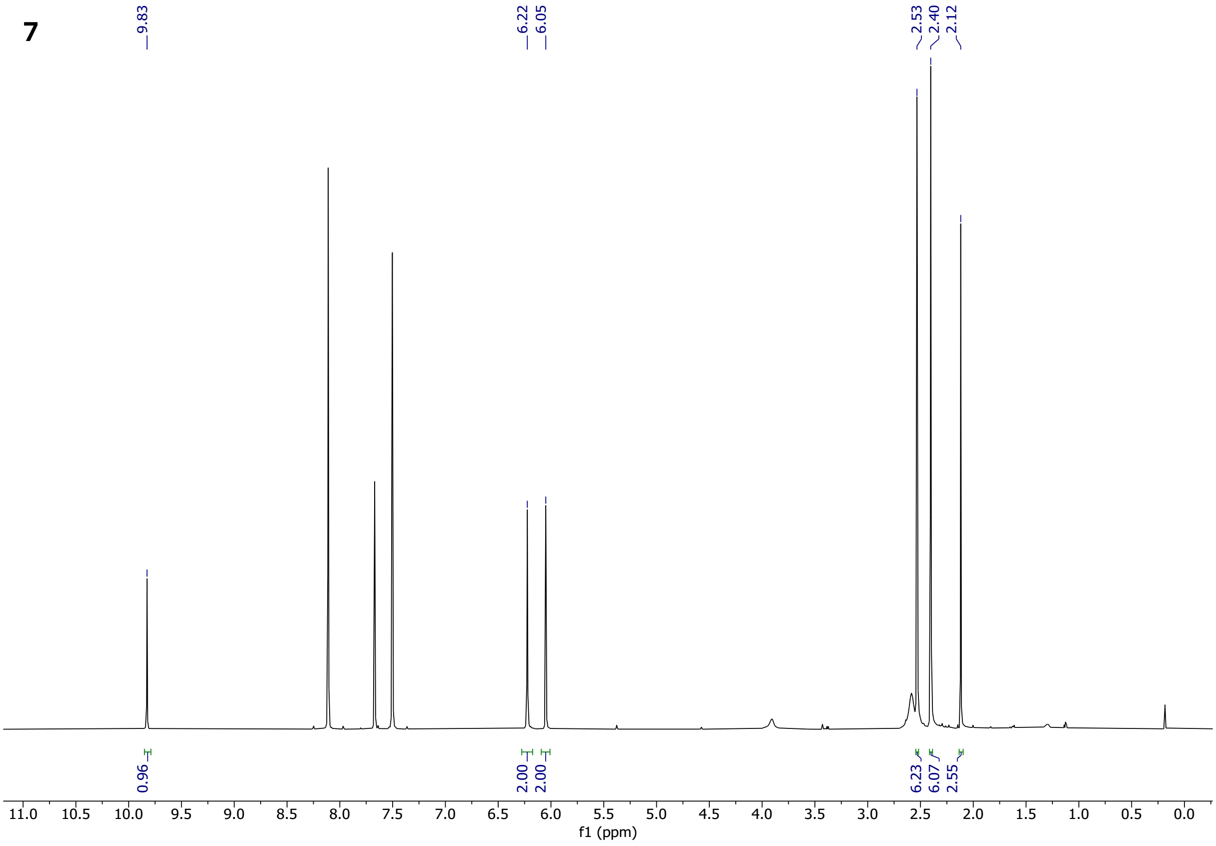

$^{13}C\{^1H\}$-NMR ($C_6D_5NO_2$, 151 MHz)

7

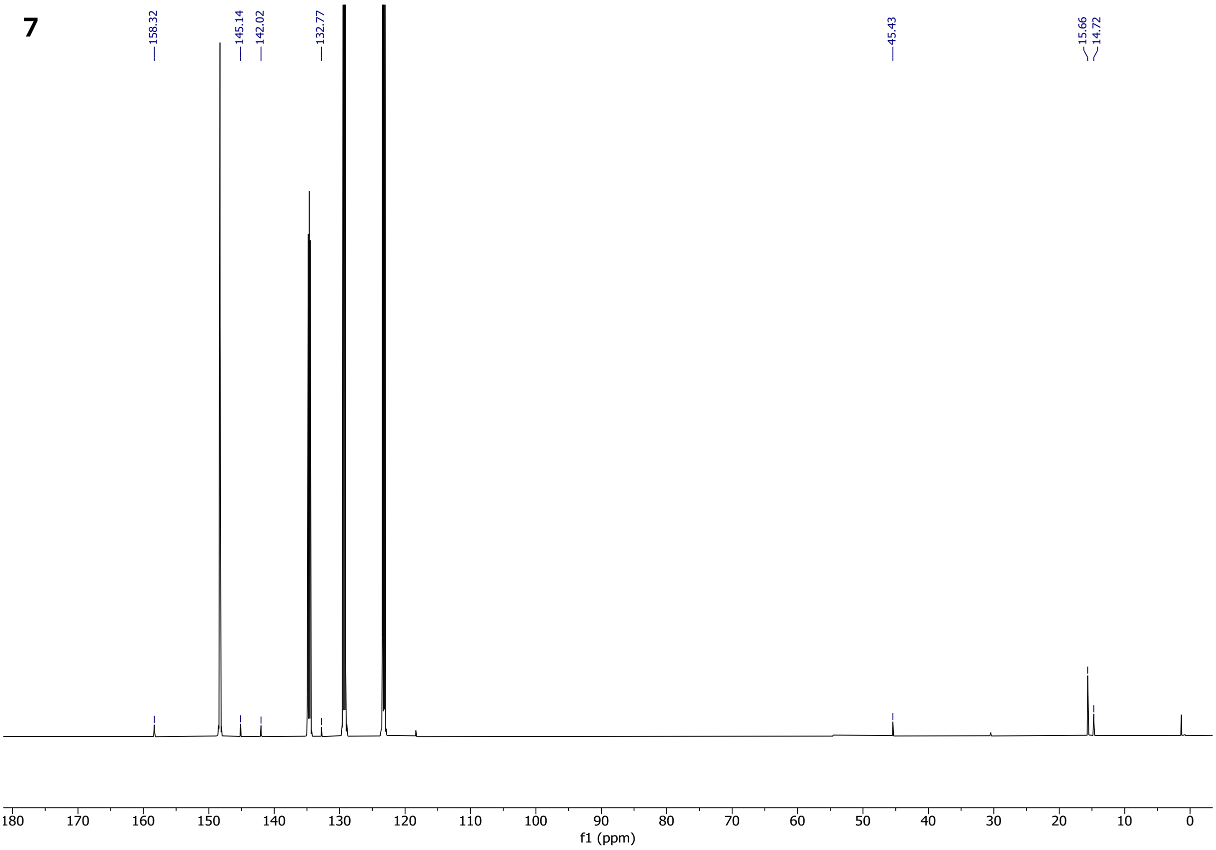

^{19}F-NMR ($C_6D_5NO_2$, 565 MHz)

7

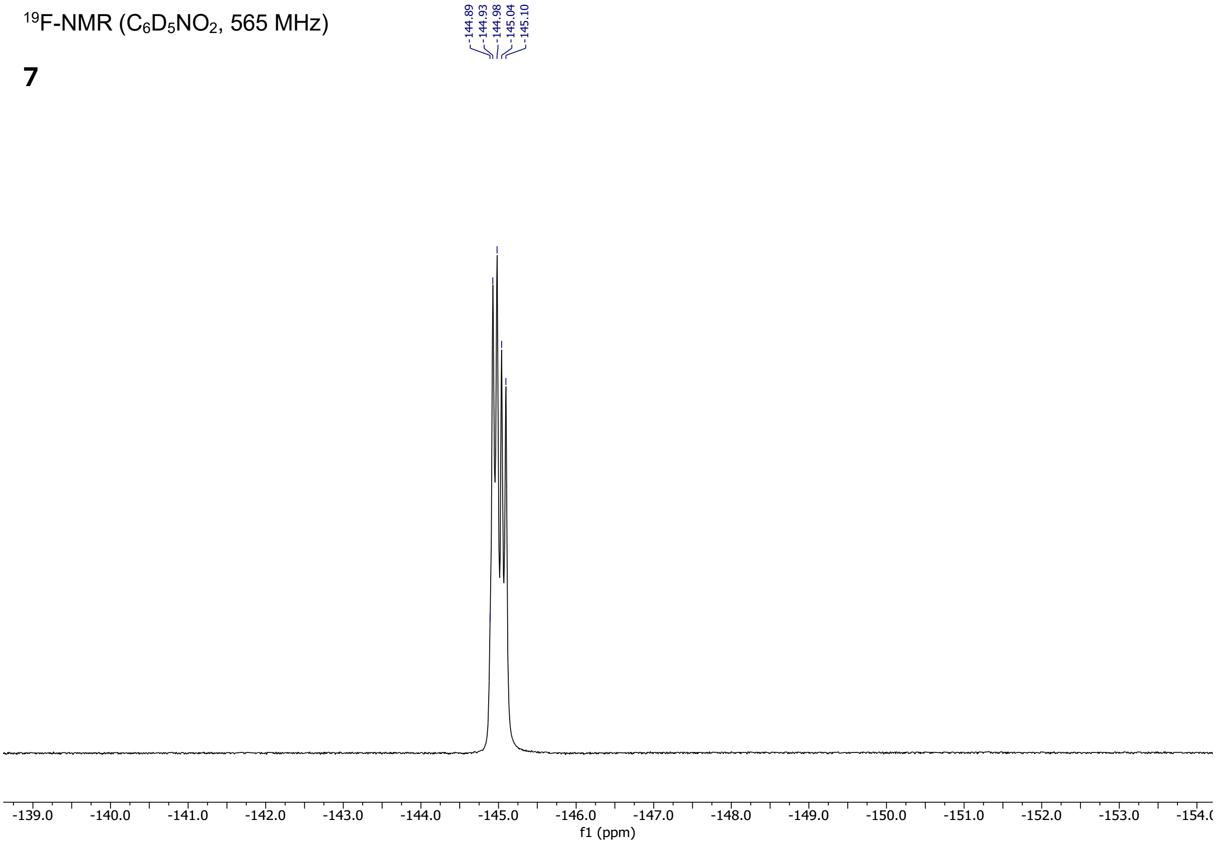

$^{11}B\{^{1}H\}$-NMR ($C_6D_5NO_2$, 193 MHz)

7

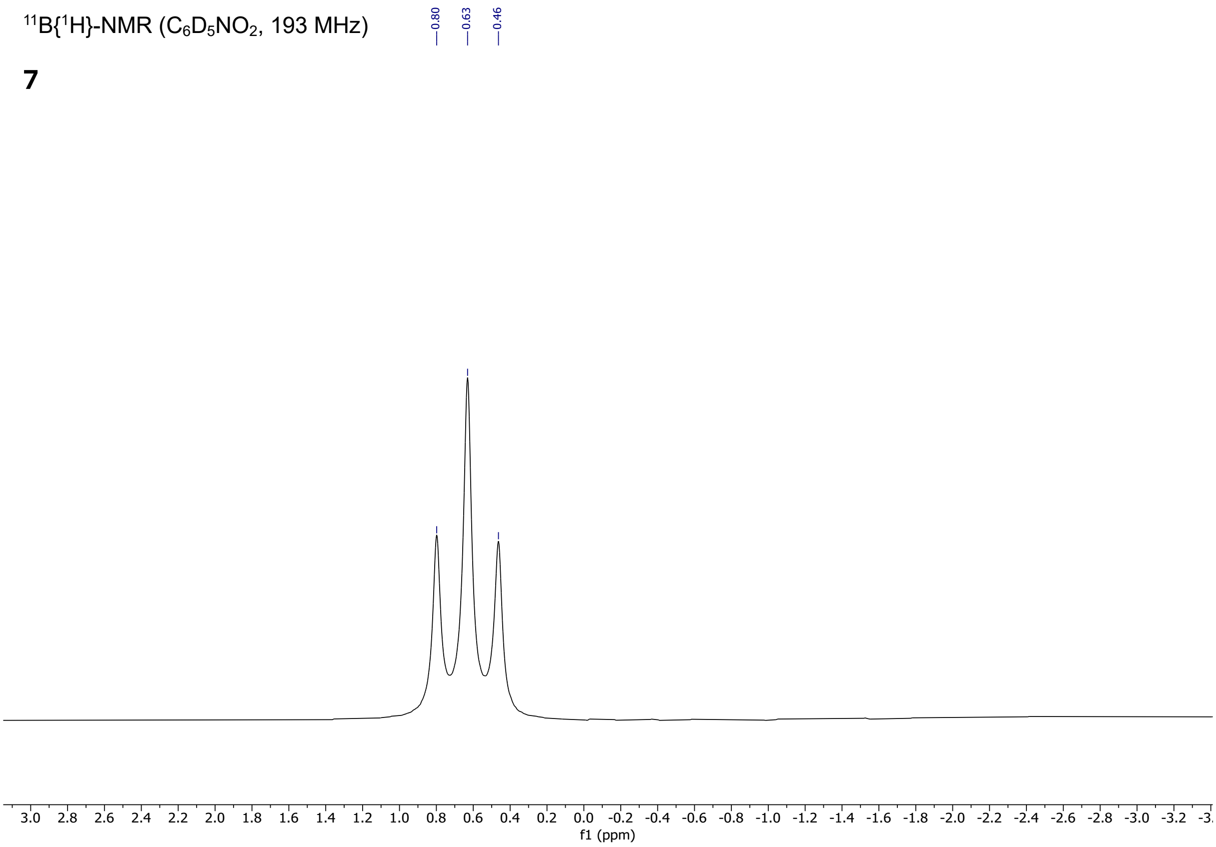

^{1}H-NMR ($C_6D_5NO_2$, 600 MHz)

8

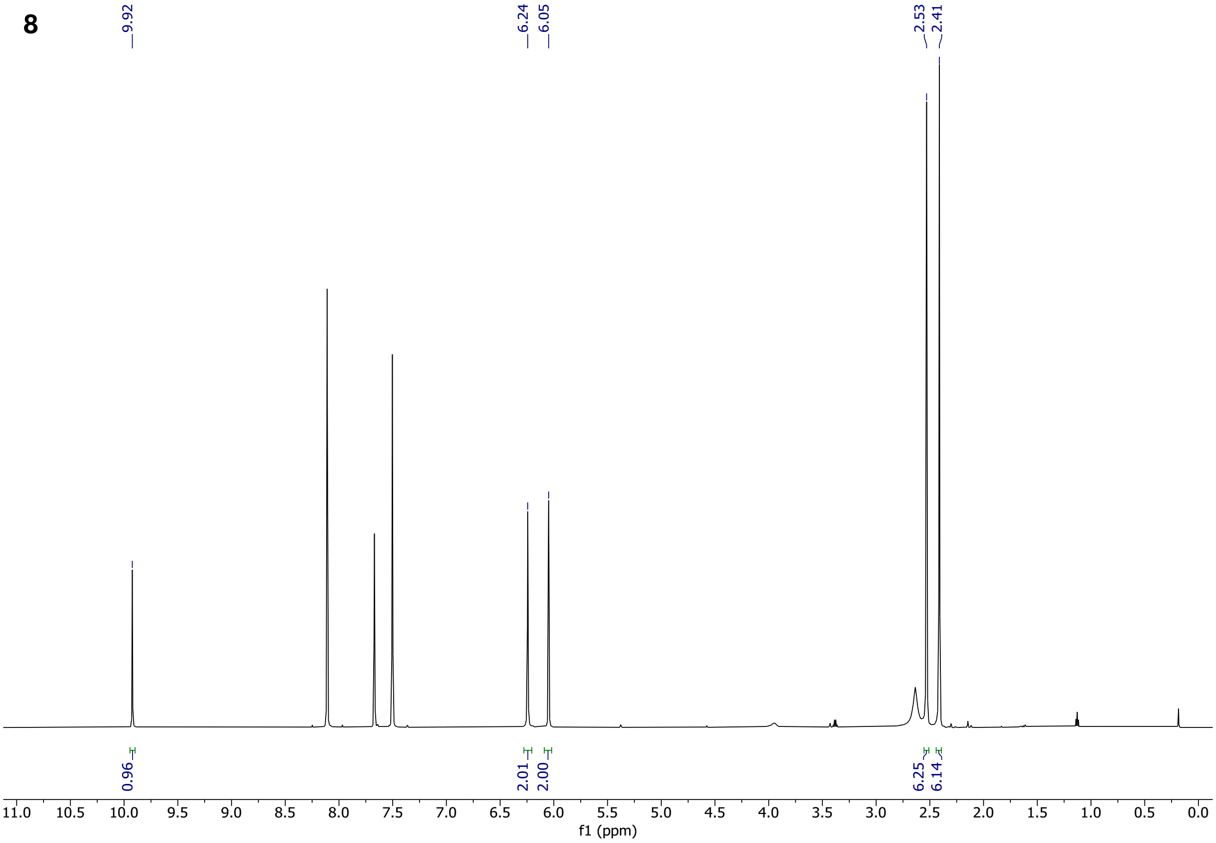

$^{13}C\{^1H\}$-NMR ($C_6D_5NO_2$, 151 MHz)

8

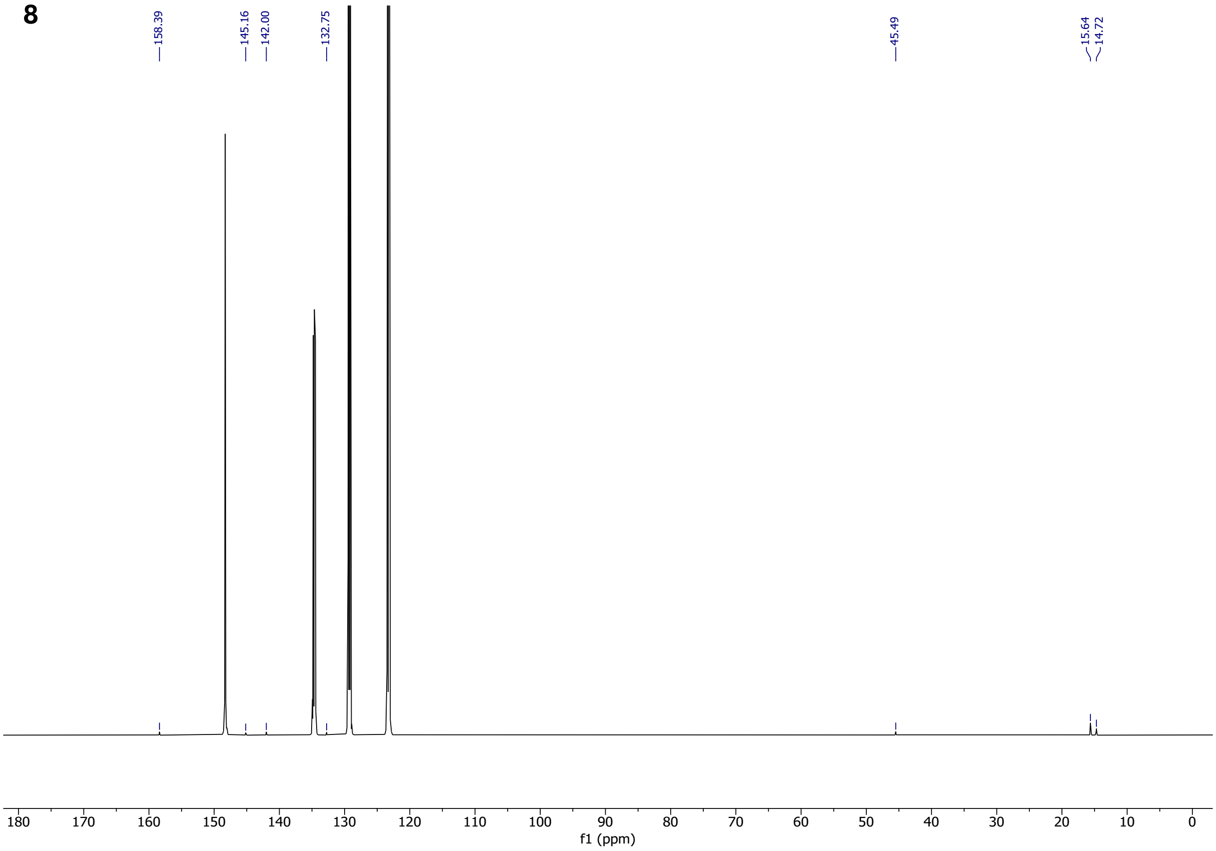

^{19}F-NMR ($C_6D_5NO_2$, 565 MHz)

8

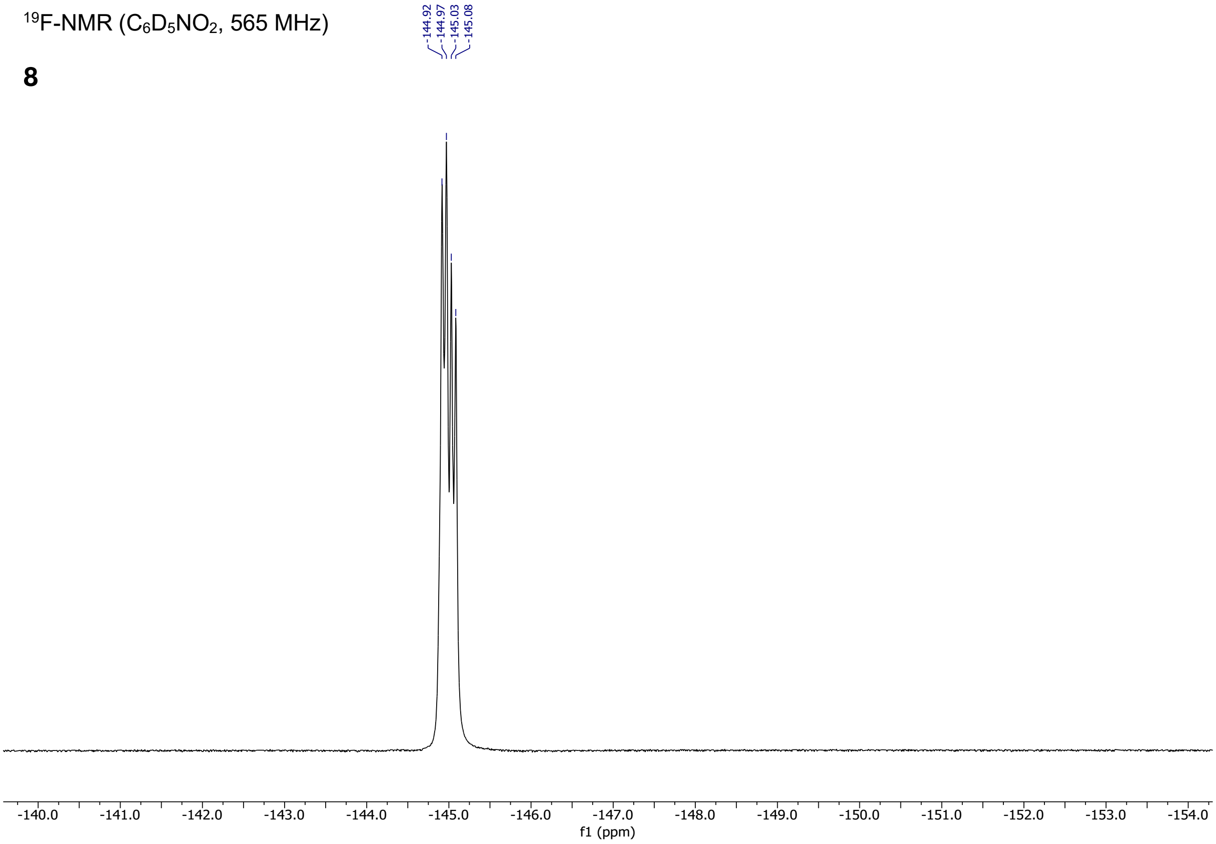

$^{11}B\{^{1}H\}$-NMR ($C_6D_5NO_2$, 193 MHz)

8

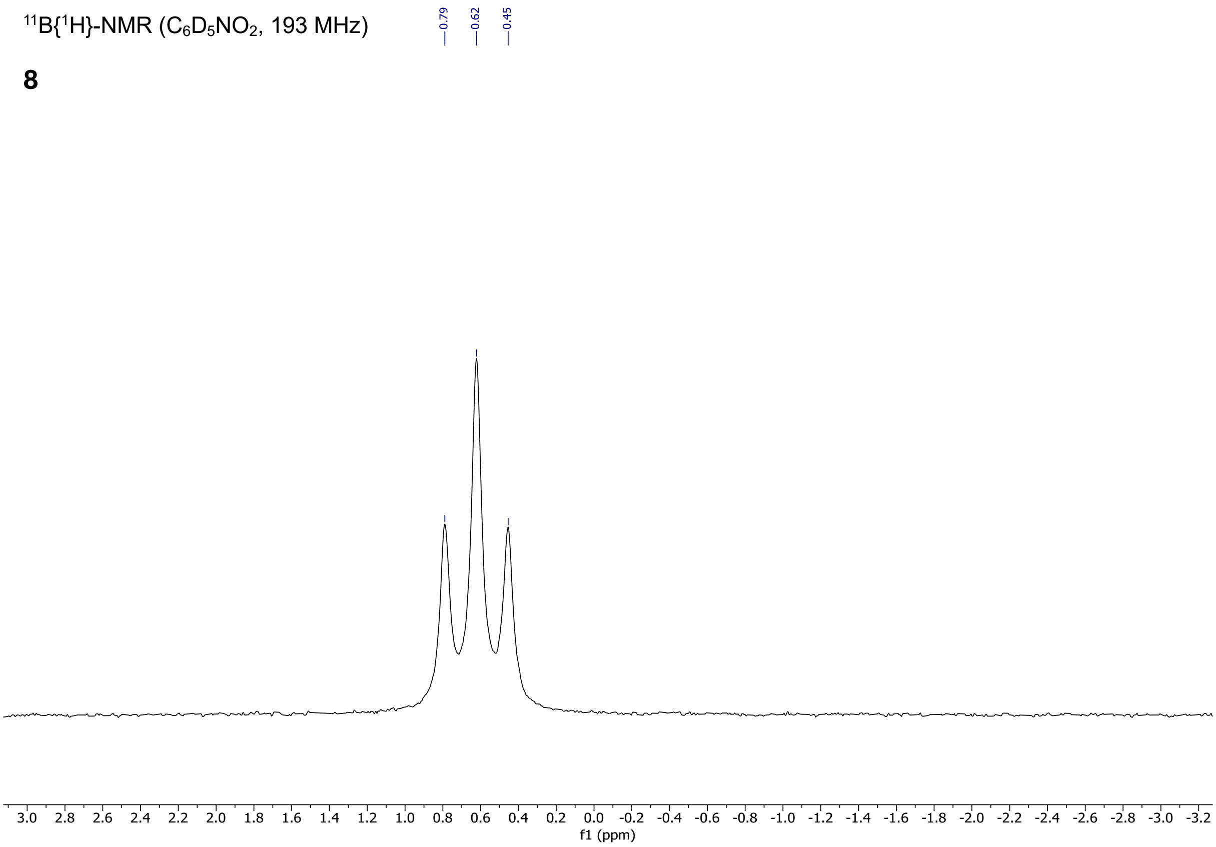

^{1}H-NMR ($C_6D_5NO_2$, 600 MHz)

10

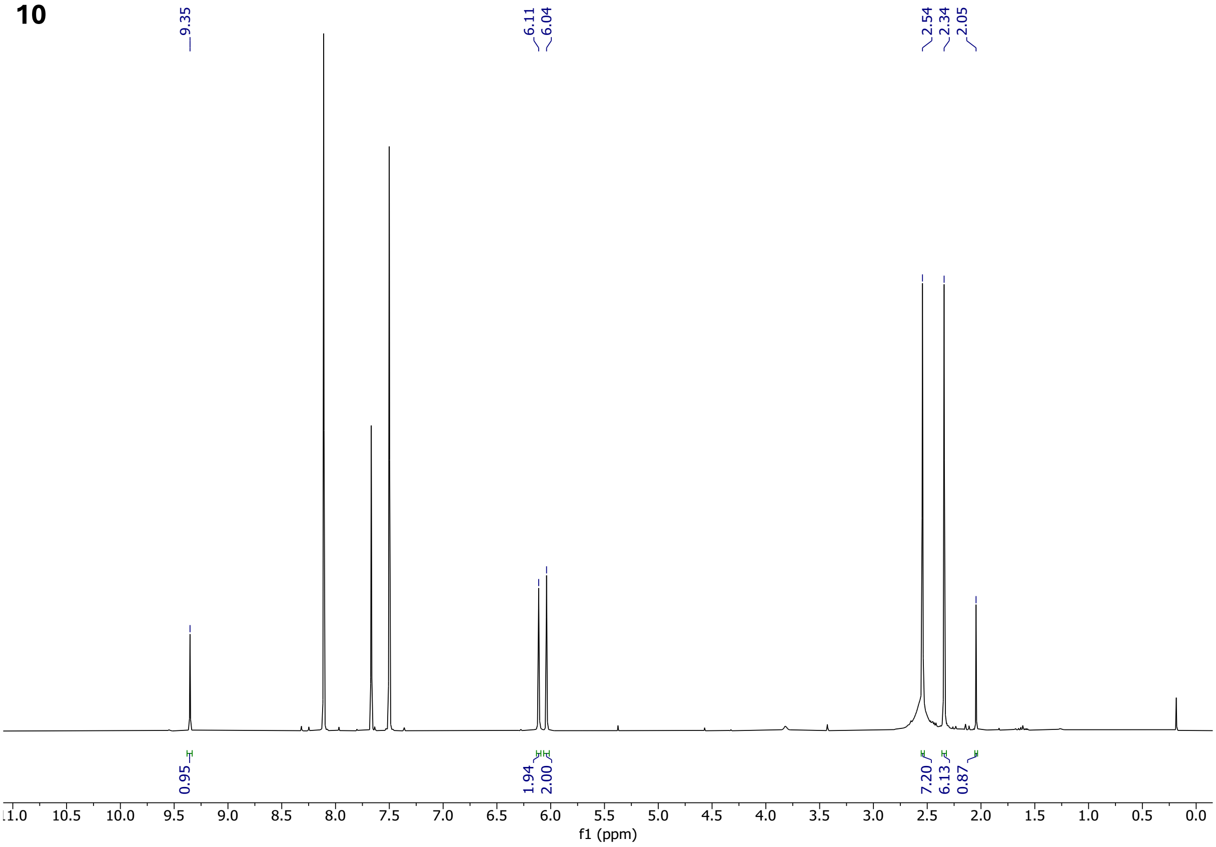

$^{13}C\{^1H\}$-NMR ($C_6D_5NO_2$, 151 MHz)

10

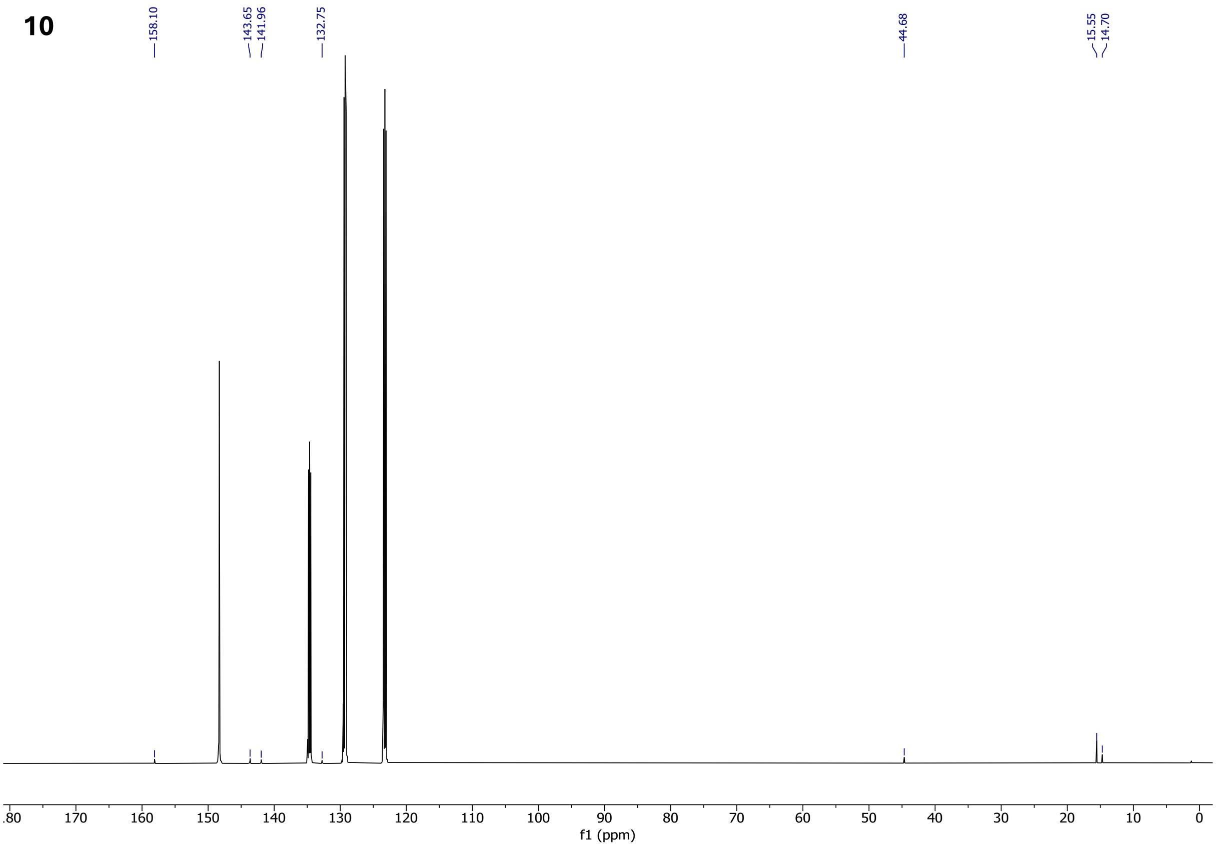

^{19}F-NMR ($C_6D_5NO_2$, 565 MHz)

10

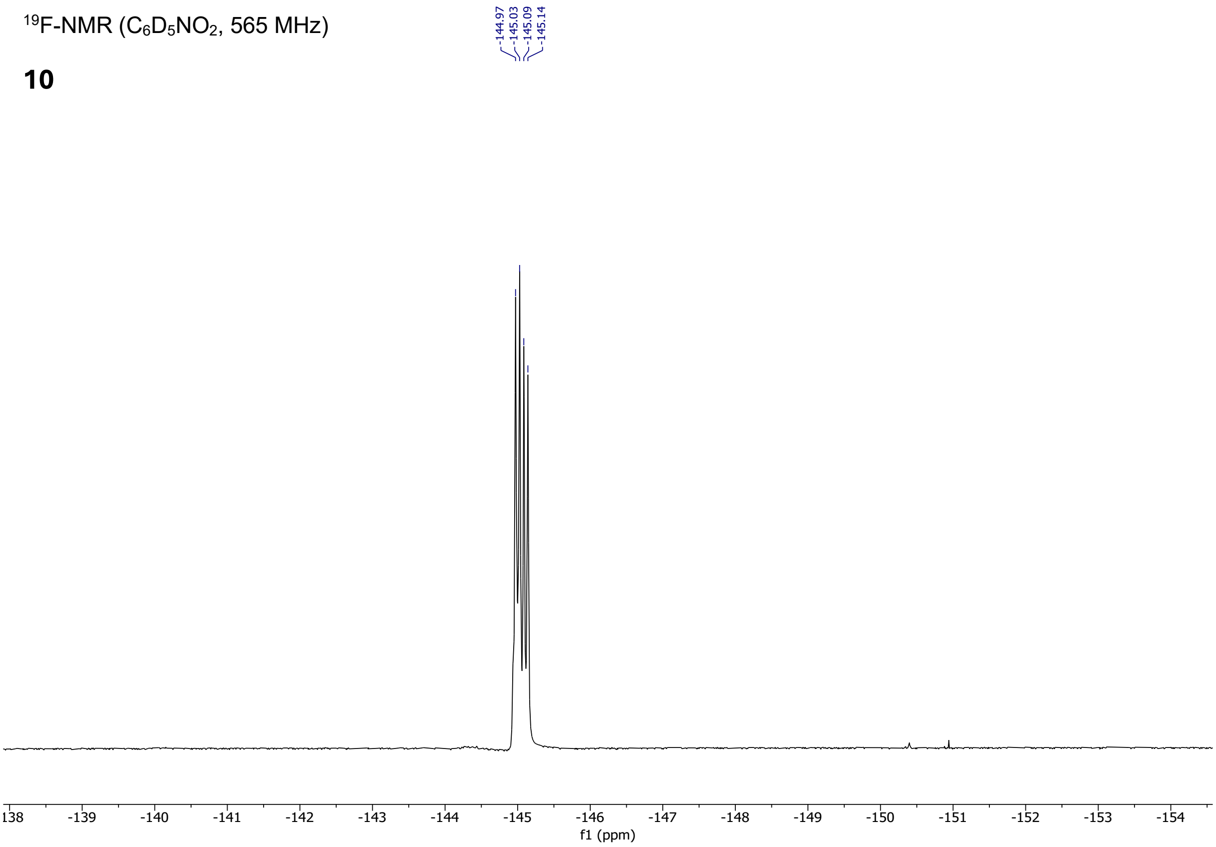

$^{11}B\{^{1}H\}$-NMR ($C_6D_5NO_2$, 193 MHz)

10

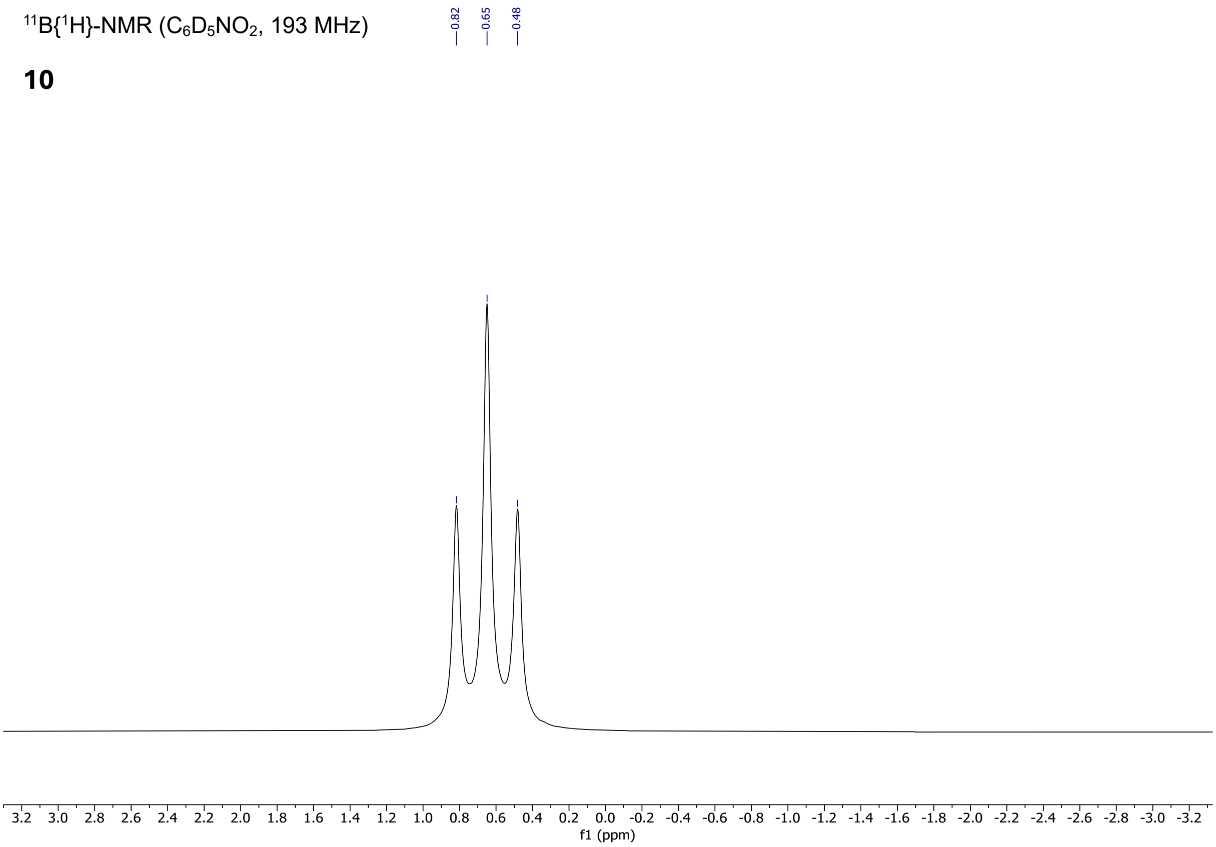

^{1}H-NMR ($C_6D_5NO_2$, 600 MHz)

11

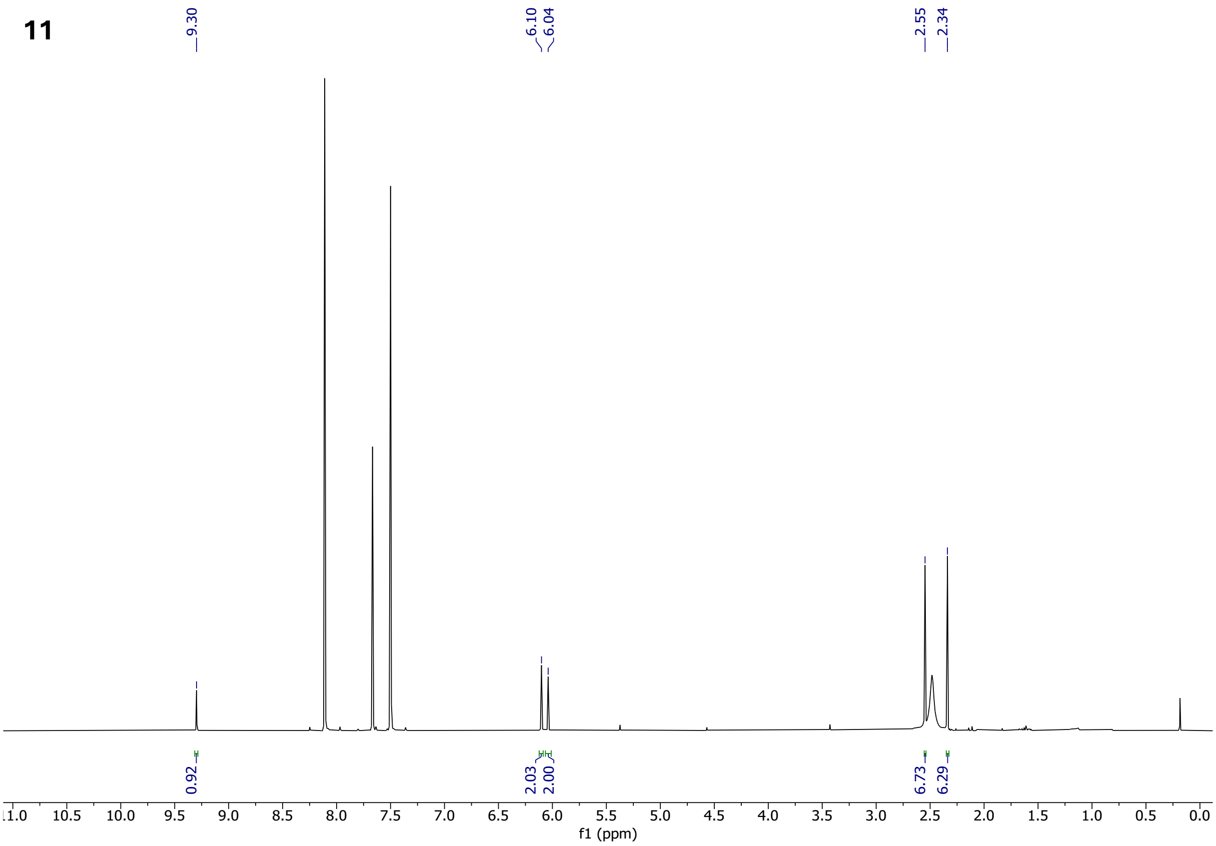

$^{13}C\{^1H\}$-NMR ($C_6D_5NO_2$, 151 MHz)

11

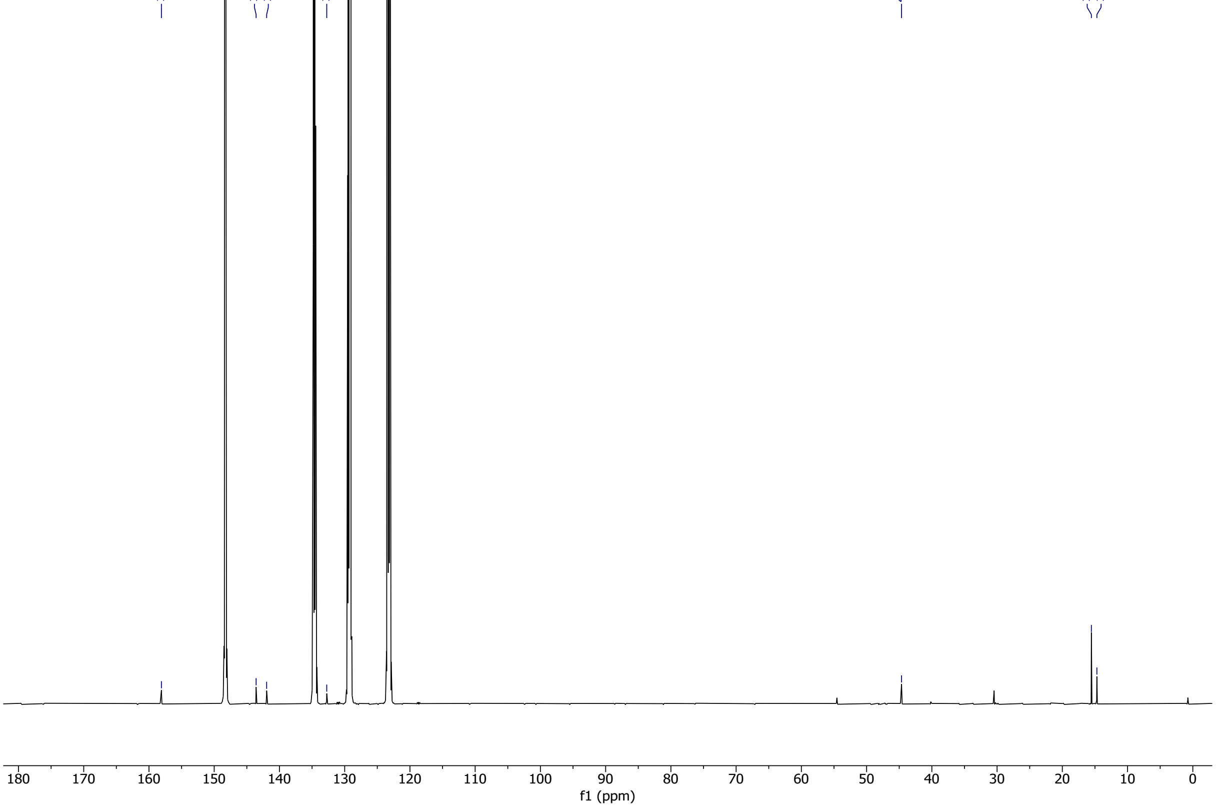

^{19}F-NMR ($C_6D_5NO_2$, 565 MHz)

11

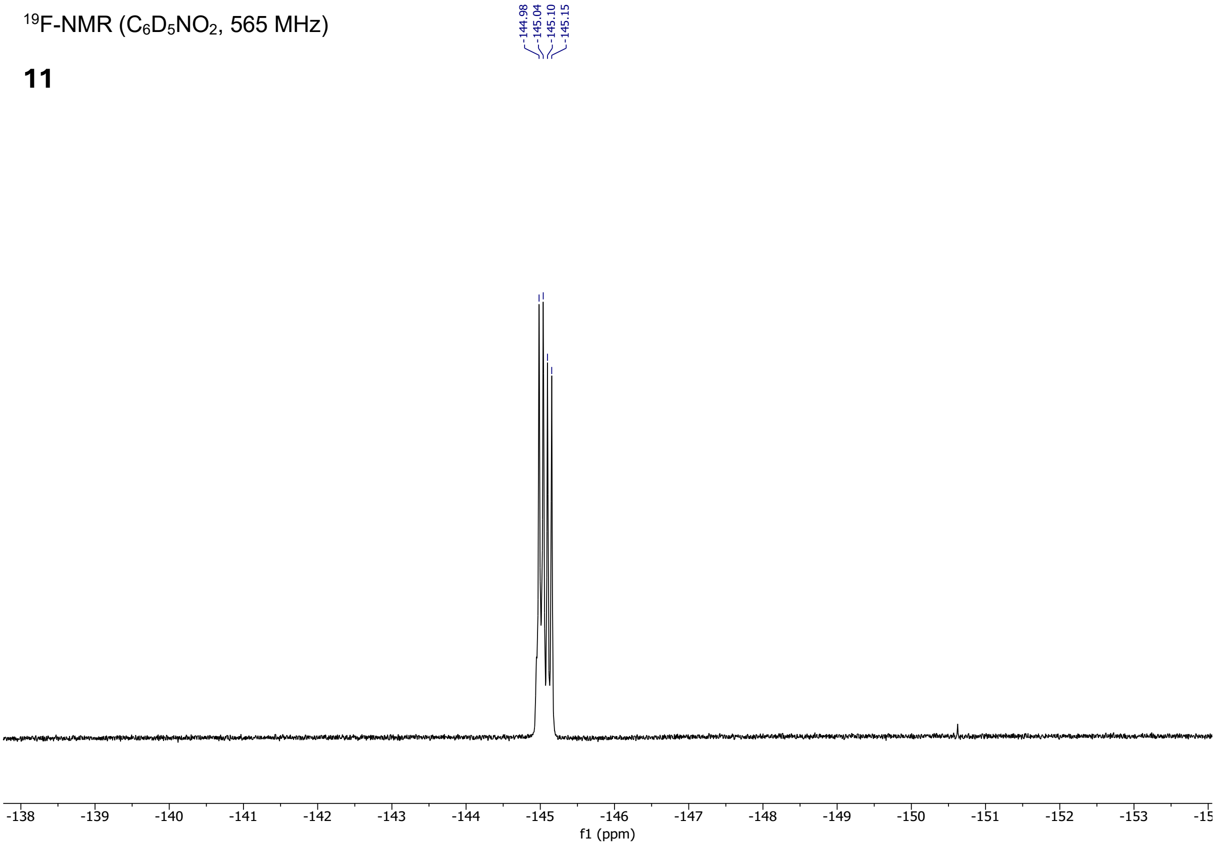

$^{11}B\{^{1}H\}$-NMR ($C_6D_5NO_2$, 193 MHz)

11

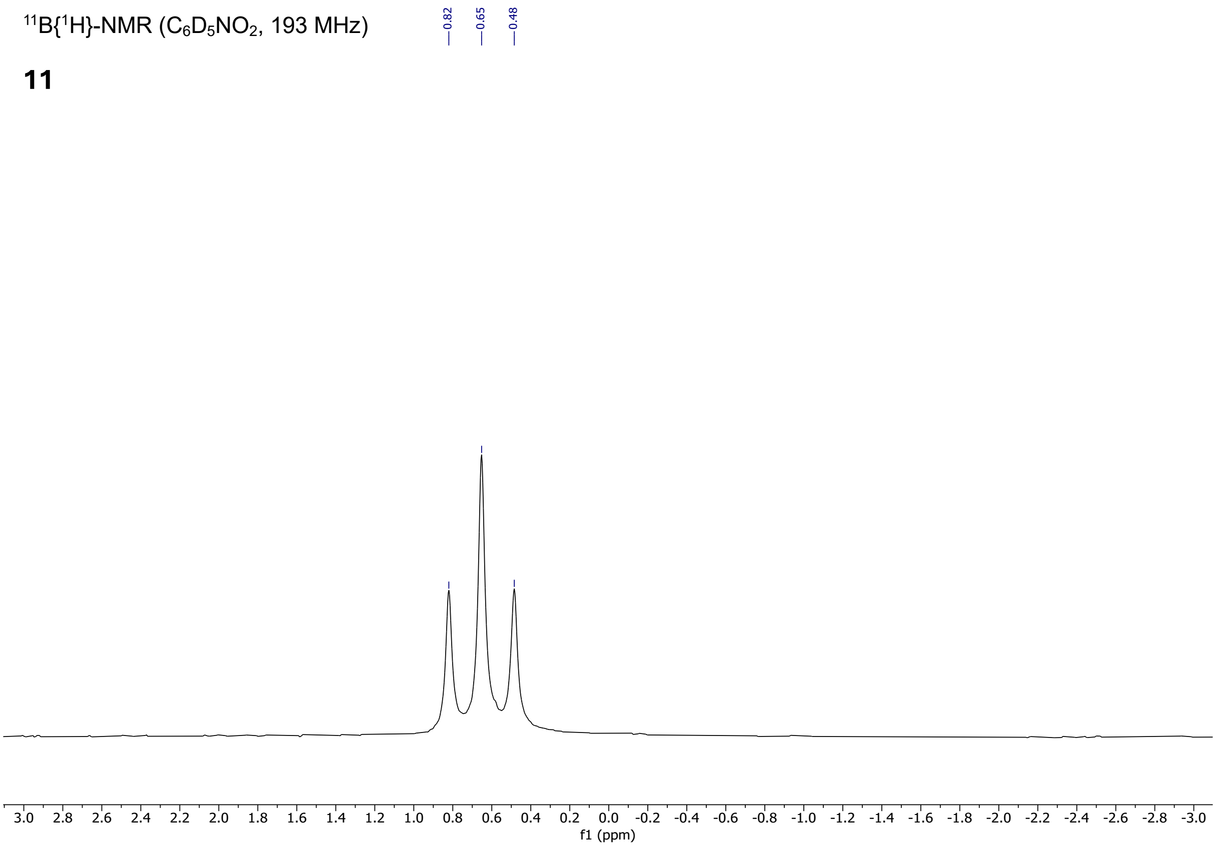

ATR-IR measurements

IR spectra were recorded with a SpectrumTwo ATR-FT-IR-spectrometer (Perkin Elmer) using a diamond ATR-unit.

IR spectra

ATR-IR

1

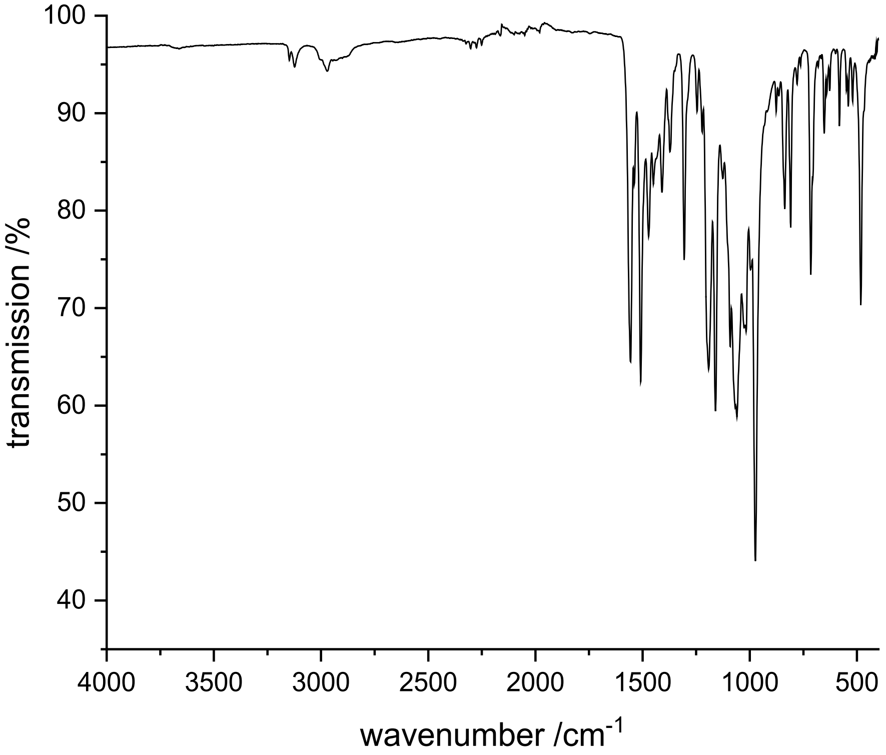

ATR-IR

2

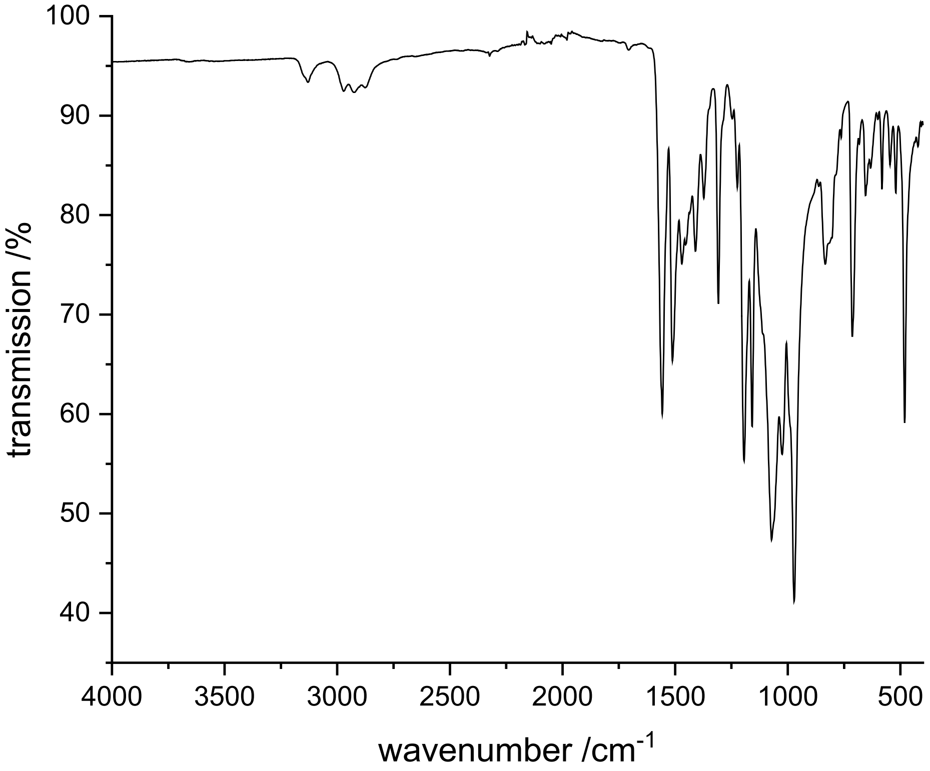

ATR-IR

3

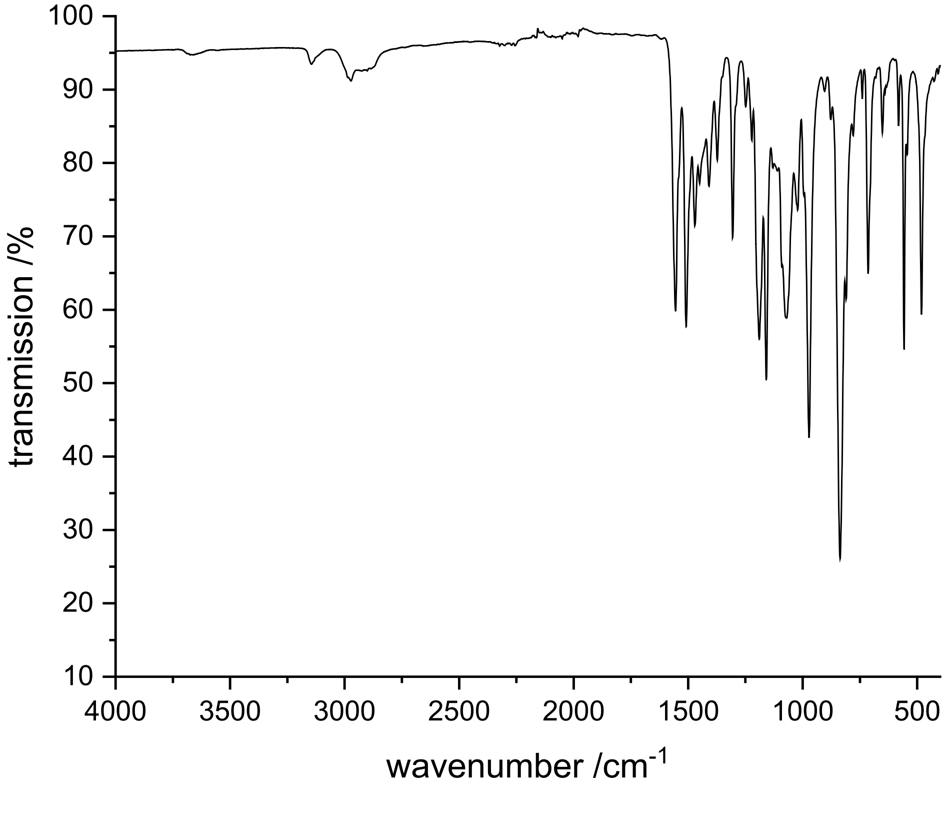

ATR-IR

4

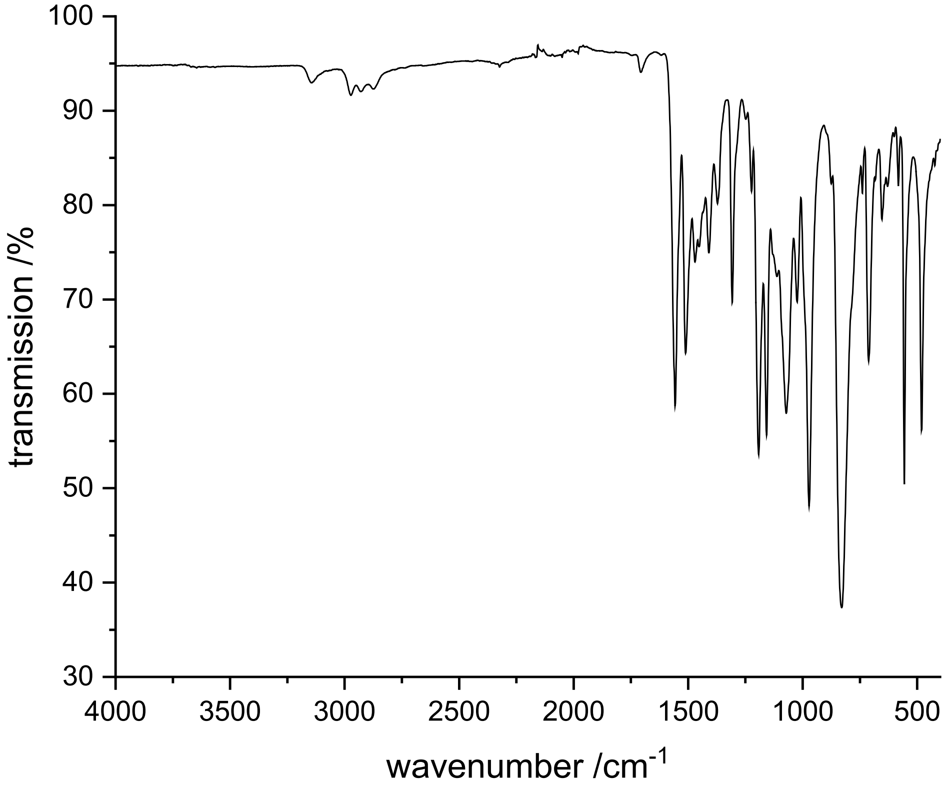

ATR-IR

6

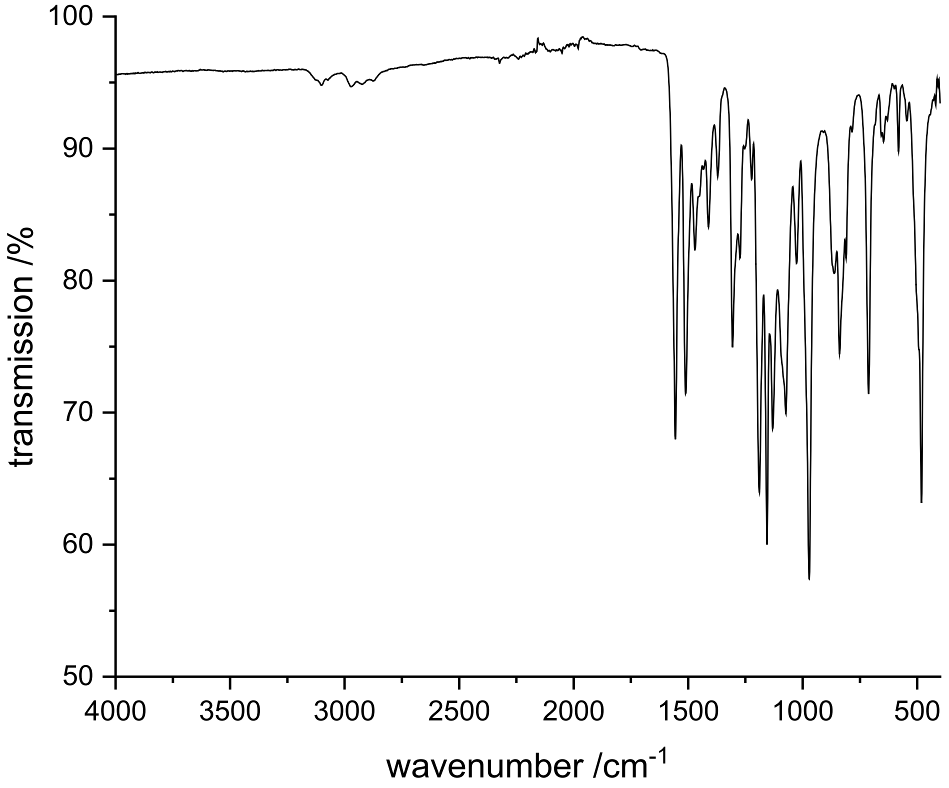

ATR-IR

7

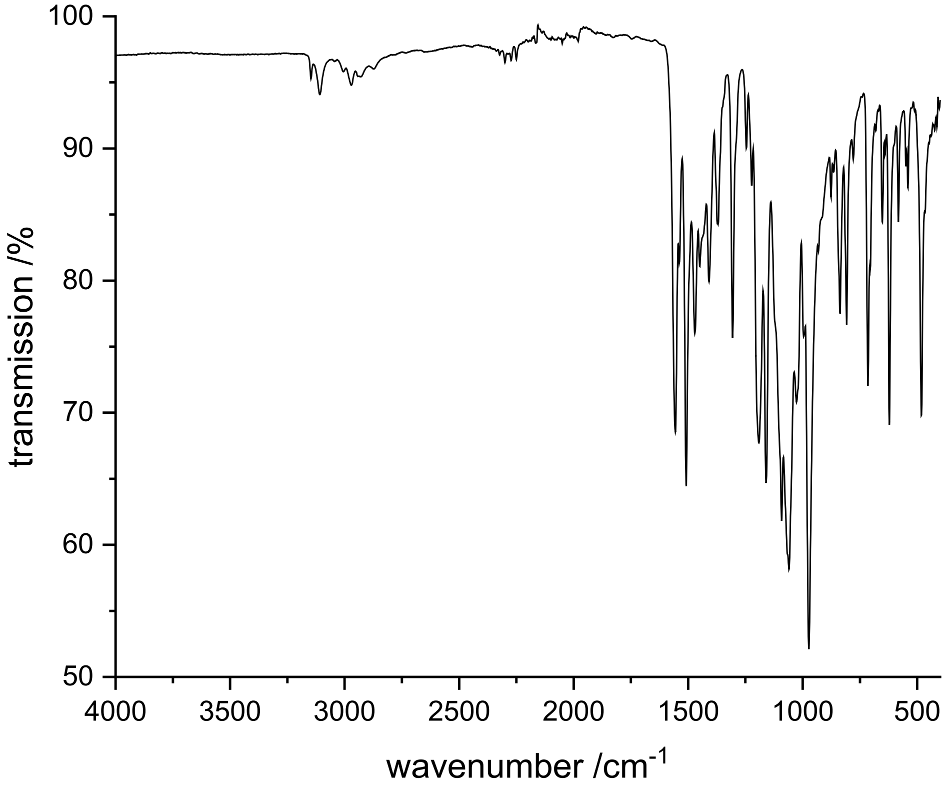

ATR-IR

8

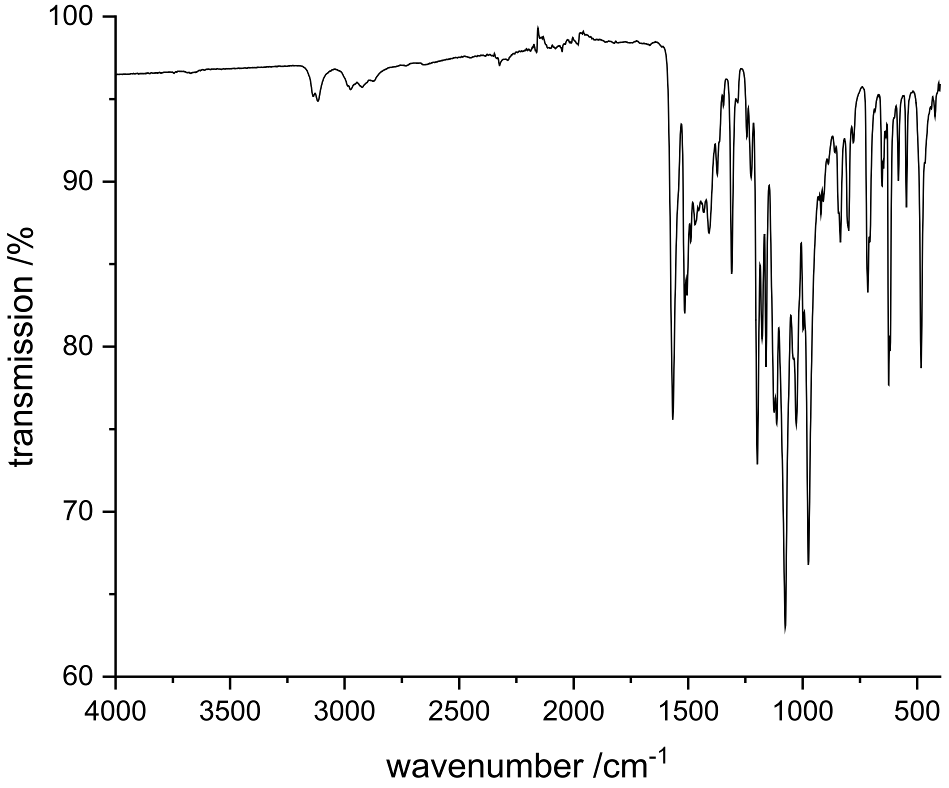

ATR-IR

10

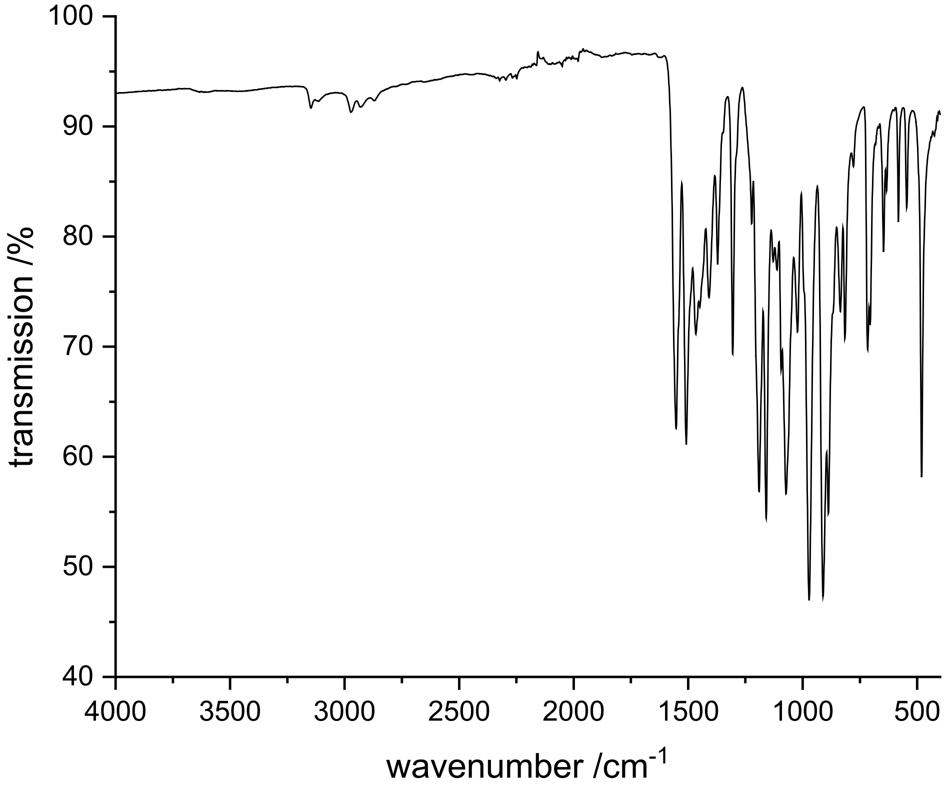

ATR-IR

11

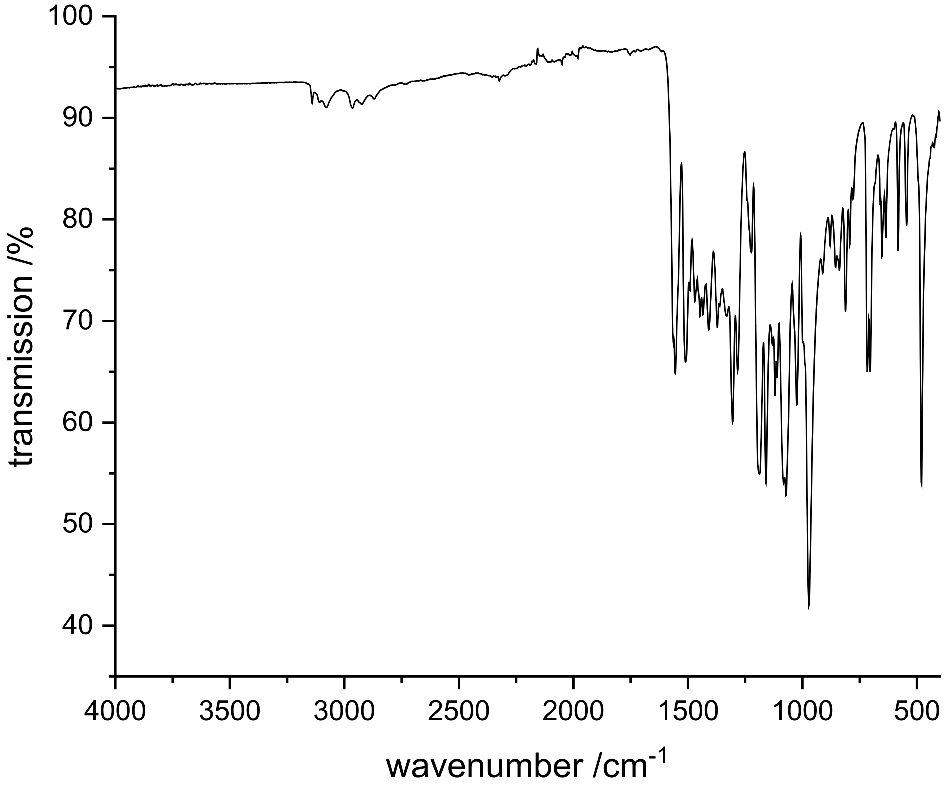

References

1. M. Huber, M. Schöbinger, J. Cirera, B. Stöger and P. Weinberger, *Eur. J. Org. Chem.*, 2024, DOI: 10.1002/ejoc.202401239, e202401239.
2. G. M. Sheldrick, *Acta Crystallogr. Sect. A: Found. Crystallogr.*, 2015, **71**, 3-8.
3. G. M. Sheldrick, *Acta Crystallogr. Sect. C: Cryst. Struct. Commun.*, 2015, **71**, 3-8.
4. T. Degen, M. Sadki, E. Bron, U. König and G. Nénert, *Powder Diffr.*, 2014, **29**, S13-S18.
